# ZBP1-mediated apoptosis and inflammation exacerbate steatotic liver ischemia/reperfusion injury

**DOI:** 10.1172/JCI180451

**Published:** 2024-05-14

**Authors:** Ran Liu, Huan Cao, Shuhua Zhang, Mao Cai, Tianhao Zou, Guoliang Wang, Di Zhang, Xueling Wang, Jianjun Xu, Shenghe Deng, Tongxi Li, Daichao Xu, Jinyang Gu

**Affiliations:** 1 Center for Liver Transplantation, Union Hospital, Tongji Medical College, Huazhong University of Science and Technology, Wuhan, Hubei, China.; 2 Interdisciplinary Research Center on Biology and Chemistry, Shanghai Institute of Organic Chemistry, Chinese Academy of Sciences, Shanghai, China.; 3 Key Laboratory of Organ Transplantation, Ministry of Education; NHC Key Laboratory of Organ Transplantation; Key Laboratory of Organ Transplantation, Chinese Academy of Medical Sciences, Wuhan, Hubei, China.

**Keywords:** Transplantation, Apoptosis

## Abstract

Steatotic donor livers are becoming more and more common in liver transplantation. However, the current use of steatotic grafts is less acceptable than normal grafts due to their higher susceptibility to ischemia/reperfusion (I/R) injury. To investigate the mechanism underlying the susceptibility of steatotic liver to I/R injury, we detected cell death markers and inflammation in clinical donor livers and animal models. We found that caspase-8–mediated hepatic apoptosis is activated in steatotic liver I/R injury. However, ablation of caspase-8 only slightly mitigated steatotic liver I/R injury without affecting inflammation. We further demonstrated that RIPK1 kinase induces both caspase-8–mediated apoptosis and cell death–independent inflammation. Inhibition of RIPK1 kinase significantly protects against steatotic liver I/R injury by alleviating both hepatic apoptosis and inflammation. Additionally, we found that RIPK1 activation is induced by Z-DNA binding protein 1 (ZBP1) but not the canonical TNF-α pathway during steatotic liver I/R injury. Deletion of ZBP1 substantially decreases the steatotic liver I/R injury. Mechanistically, ZBP1 is amplified by palmitic acid–activated JNK pathway in steatotic livers. Upon I/R injury, excessive reactive oxygen species trigger ZBP1 activation by inducing its aggregation independent of the Z-nucleic acids sensing action in steatotic livers, leading to the kinase activation of RIPK1 and the subsequent aggravation of liver injury. Thus, ZBP1-mediated RIPK1-driven apoptosis and inflammation exacerbate steatotic liver I/R injury, which could be targeted to protect steatotic donor livers during transplantation.

## Abstract 2

ZBP1 is increased by the palmitic acid–activated JNK pathway and mediates steatotic liver ischemia-reperfusion injury by triggering RIPK1–dependent apoptosis and inflammation.

## Graphical abstract

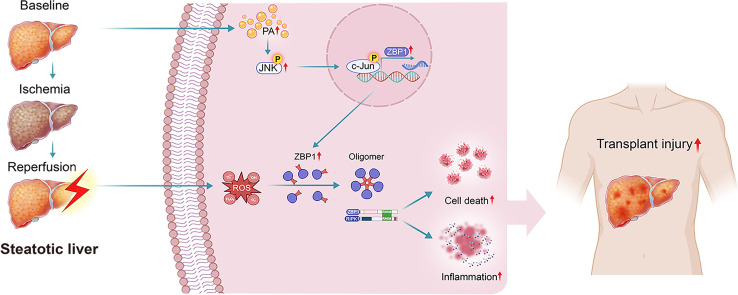

## Introduction

End-stage liver disease is the leading cause of mortality in digestive diseases, with liver transplantation as the sole curative intervention (1). The scarcity of available donor livers has led to the emergence of marginal donor livers, including older donors, donation after circulatory death grafts, livers infected with hepatitis viruses, and steatotic donor livers (2,3). The escalating prevalence of metabolic dysfunction-associated steatotic liver disease (MASLD) due to changing dietary structures underscores the urgent need to optimize the utilization of steatotic donor livers (4). The principal limitation of steatotic donor livers is their susceptibility to ischemia/reperfusion (I/R) injury, which greatly compromises liver function and potentially leads to primary organ nonfunction or early allograft dysfunction (5). Upon reperfusion, hepatocytes produce substantial amounts of reactive oxygen species (ROS) and secrete various proinflammatory cytokines. As a result, immune cells, particularly neutrophils, are recruited to the liver from the circulation and directly damage liver tissues (6). However, the mechanism underlying the susceptibility of steatotic livers to I/R injury remains unclear.

Programmed cell death, mediated by dedicated molecular machines, plays important roles in health and disease (7). The caspase family of cysteine proteases serve as key regulators of programmed cell death. Apoptosis, the first identified regulated form of cell death, is executed by caspase-3/7, which become activated by upstream caspase-8 or caspase-9 in response to extracellular cell death–inducing cytokines or intracellular stress signals, respectively (8). The same cell death-inducing cytokines also cause necroptosis mediated by receptor-interacting serine/threonine-protein kinase 1 (RIPK1), RIPK3, and MLKL when caspase-8 is inhibited (9). Inflammatory caspases, including caspase-1/4/5/11, instead induce pyroptosis by cleaving gasdermin D (10). Additionally, disturbance of peroxidation of polyunsaturated fatty acid of membrane phospholipids triggers a caspase-independent necrotic death, called ferroptosis (11). It has been shown that apoptosis participates in liver I/R injury and the pancaspase inhibitor IDN-6556 protects against liver transplantation–induced apoptosis and injury (12). Nevertheless, it was reported that necroptosis also plays a significant role in liver I/R injury (13), which was challenged by recent studies showing that RIPK3 is epigenetically silenced in mouse and human hepatocytes and rendered them unable to undergo necroptosis (14–16). In addition, pyroptosis was also implicated in liver I/R injury (17). Further, recent studies suggest that ferroptosis also mediates liver I/R injury (18-20), while an earlier study showed that lipid peroxidation is unlikely the primary mechanism of parenchymal cell injury during reperfusion (21). Thus, the mechanisms underlying hepatocyte death in liver I/R injury are still elusive. Further, whether and how these cell death modalities contribute to steatotic liver I/R injury remains to be determined.

Z-DNA binding protein 1 (ZBP1) is a newly identified innate immune sensor for the unusual left-handed Z-nucleic acids (Z-NA) that initiates cell death (22). ZBP1-activated cell death was firstly described in influenza A virus infection and further extended to infections of other pathogens (23). Interestingly, ZBP1 also activates cell death under sterile conditions when the scaffold function of RIPK1 is absent (24). Further, mutation of ADAR1, an RNA-editing enzyme, causes ZBP1-dependent immunopathology in sterile settings (25). Therefore, in addition to exogenous pathogen-associated Z-NA, ZBP1 can also be activated by specific endogenous Z-NA, which may lead to cell death and inflammatory diseases. However, it is unclear whether ZBP1 might be activated by signals other than Z-NA. Moreover, to our knowledge, the role of ZBP1-mediated cell death and inflammation has never been reported in the setting of steatotic liver I/R injury.

In this study, we present compelling evidence showing that ZBP1 specifically mediates steatotic liver I/R injury by triggering RIPK1-dependent apoptosis and inflammation. ZBP1 is specifically increased in steatotic liver by palmitic acid–activated (PA-activated)JNK pathway and is activated through a mechanism independent of the Z-NA sensing action. We show that ZBP1 is activated by ROS during I/R, which promotes the aggregation of ZBP1 and induces the kinase activation of RIPK1 that triggers caspase-8–mediated apoptosis and cell death–independent inflammation. Deletion of ZBP1 or genetic inactivation of RIPK1 in the liver decreases the steatotic liver I/R injury. Thus, ZBP1 appears to have a secondary function that orchestrates host responses to steatotic liver I/R injury and represents a potential therapeutic target for steatotic liver I/R injury during donor liver transplantation.

## Results

### Aggravation of hepatic apoptosis and inflammation in steatotic liver I/R injury.

**Figure 1:**
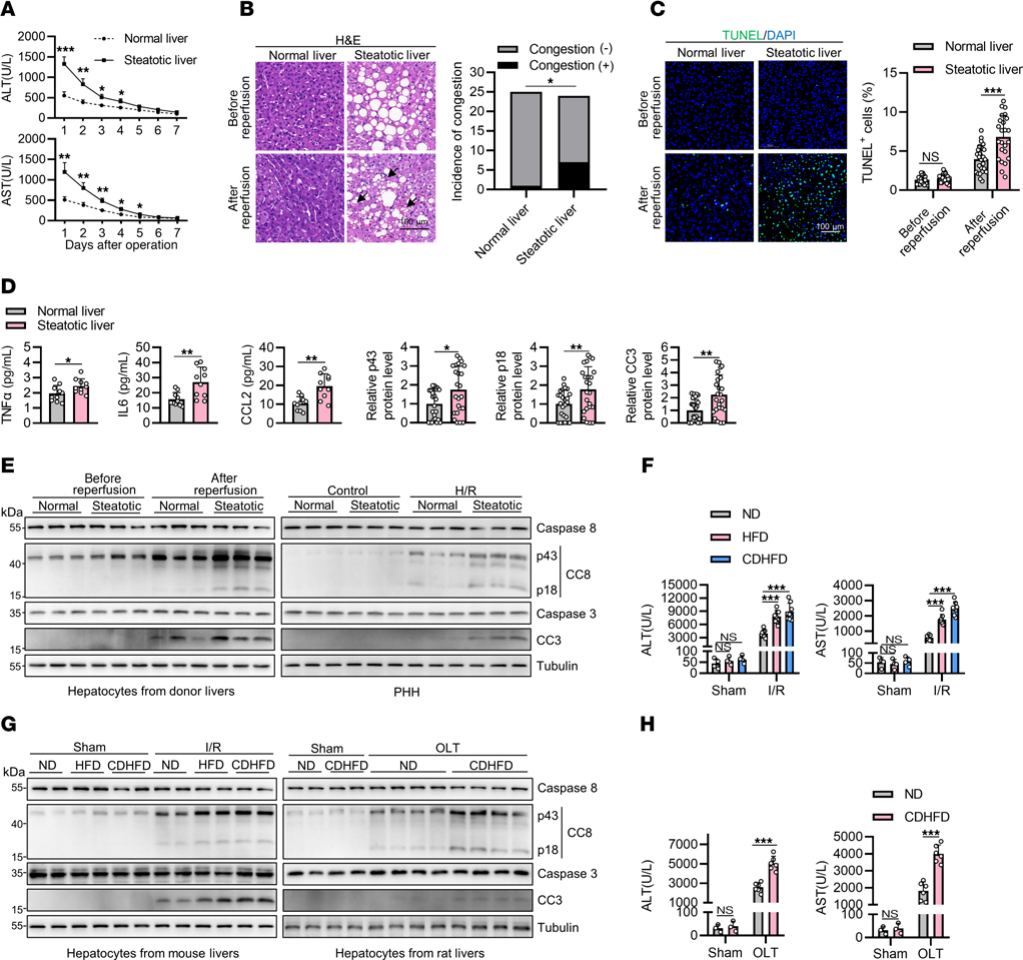
Apoptosis and inflammation are exacerbated in steatotic liver I/R injury. (**A**) Serum ALT and AST levels of normal donor liver or steatotic donor liver recipients within 7 days after transplantation. (**B** and **C**) Prereperfusion and postreperfusion donor liver specimens were analyzed with H&E staining (**B**) and TUNEL staining (**C**). Arrows indicate congestion (**B**). *n* = 25 for normal donor livers and *n* = 24 for steatotic donor livers. (**D**) Recipients’ serum levels of the proinflammatory cytokines (*n* = 10) on POD1. (**E**) Left: Representative immunoblot result of cell death markers in hepatocytes isolated from pre- and postreperfusion donor livers. Protein expression was normalized to tubulin levels and shown as relative values. *n* = 25 for normal donor livers and *n* = 24 for steatotic donor livers. Right: PHH cell were isolated and cultured in vitro. After H/R challenge, the death markers were analyzed (*n* = 6). (**F**–**H**) ND-, HFD-, and CDHFD-fed mice (*n* = 8) underwent 1 hour ischemia/6 hours reperfusion operation and donor livers from ND- or CDHFD-fed rats (*n* = 6) underwent 18 hours cold storage/6 hours reperfusion. Serum ALT/AST levels (**F** and **H**) and cell death markers in isolated hepatocytes (**G**) were detected. All data are presented as the mean ± SD. *P < 0.05, **P < 0.01, ***P < 0.001. 2-way ANOVA, post hoc Bonferroni’s test (**A**, **C**, **F**, and **H**). Fisher’s exact test (**B**). Unpaired 2-tailed Student’s *t* test (**D** and **E**). Scale bars: 100 μm

We first confirmed the susceptibility of steatotic livers to I/R injury during human liver transplantation and observed significantly elevated serum levels of alanine transaminase (ALT) and aspartate transaminase (AST), indicative of liver injury, in recipients of steatotic donor livers during the initial 4 days following transplantation, compared with those of normal donor livers (Figure 1A). As expected, when compared with normal donor livers, steatotic donor livers exhibited a higher incidence of sinusoidal congestion (Figure 1B), higher percentage of cell death as determined by terminal deoxynucleotidyl transferase dUTP nick end labeling (TUNEL) assay (Figure 1C), and higher levels of neutrophil infiltration as marked by myeloperoxidase (MPO) staining (Supplemental Figure 1A; supplemental material available online with this article; https://doi.org/10.1172/JCI180451DS1) after reperfusion but not before reperfusion. Additionally, serum levels of proinflammatory cytokines, including TNF-α, IL6, and chemokine CCL2, were markedly elevated on the first day after operation in recipients of steatotic donor livers than that of normal donor livers (Figure 1D), indicating aggravated inflammatory response in recipients of steatotic donor livers.

We then confirmed the modalities of hepatic cell death during steatotic liver I/R injury. To avoid the influence of nonparenchymal cells, we isolated hepatocytes from normal and steatotic donor livers before and after reperfusion and subjected them to immunoblotting analysis. We observed a substantial increase in the levels of apoptosis, marked by cleaved caspase-8 (CC8) and cleaved caspase-3 (CC3) (8) in steatotic donor livers after reperfusion (Figure 1E), which was confirmed by immunostaining of CC3 in the donor livers (Supplemental Figure 1B). In contrast, we did not detect activation markers of pyroptosis as marked by cleaved N-terminal GSDMD (10) or necroptosis as marked by p-RIPK3(S227) and p-MLKL(S358) (9) in hepatocytes of steatotic donor livers after reperfusion (Supplemental Figure 1C). We also isolated primary human hepatocytes (PHH) from donor livers before reperfusion, cultured them in vitro, and exposed them to hypoxia-reoxygenation (H/R), an in vitro model mimicking liver I/R injury (26). We found that steatotic liver–derived PHH exhibited higher levels of apoptosis than normal liver–derived PHH (Figure 1E). Similarly, pyroptosis and necroptosis were not activated in steatotic hepatocytes in this setting (Supplemental Figure 1D). In addition, the levels of ferroptosis-related proteins, including GPX4, FSP1, and ACSL4 (27), as well as lipid peroxidation as determined by 4-HNE staining (28), were similar between normal and steatotic livers (Supplemental Figure 1, C and E), excluding the possibility that ferroptosis contributes to the increased sensitivity of steatotic liver to I/R injury.

We next conducted animal experiments modeling steatotic liver I/R injury. To this end, we fed mice with a high-fat-diet (HFD) or a choline-deficient high-fat-diet (CDHFD) to induce moderate or severe steatosis, respectively, and then subjected these mice to I/R operation. We observed significantly elevated serum levels of ALT and AST (Figure 1F) as well as injury area marked by patchy areas of hypereosinophilia, loss of cellular morphology, and nucleus shrinkage in the livers in both HFD- and CDHFD-fed mice compared with the normal diet–fed (ND-fed) mice after I/R (Supplemental Figure 1F). The number of TUNEL-positive cells was also higher in the livers of HFD- and CDHFD-fed mice than that of ND-fed mice after I/R (Supplemental Figure 1G). Similar to human steatotic donor livers after reperfusion, CC8 and CC3 were elevated in the hepatocytes of both HFD- and CDHFD-fed mice compared with the ND-fed mice after I/R (Figure 1G and Supplemental Figure 1, H and I). MPO staining revealed higher levels of neutrophil infiltration in the livers of HFD- and CDHFD-fed mice than that of ND-fed mice after I/R (Supplemental Figure 1J), indicative of aggravated inflammation, which was further confirmed by the elevated serum levels of TNF-α, IL6, and CCL2 (Supplemental Figure 1K).

To consolidate these findings, we then conducted rat orthotopic liver transplantation (OLT) and utilized a procedure mimicking clinical liver transplantation. Consistently, we observed increased liver injury (Figure 1H and Supplemental Figure 2, A and B) and hepatic apoptosis (Figure 1G and Supplemental Figure 2, C and D) in livers from CDHFD-fed rats compared with those of ND-fed rats. We also observed increased inflammation in recipients of steatotic donor livers after OLT (Supplemental Figure 2, E and F). Finally, primary mouse hepatocytes (PMH) isolated from the livers of HFD- and CDHFD-fed mice showed higher levels of apoptosis and inflammation, but not necroptosis and pyroptosis, after H/R challenge compared with those of ND-fed mice (Supplemental Figure 2, G-I). Thus, these findings suggested that hepatic apoptosis and inflammation are exacerbated during steatotic liver injury.

### Caspase-8–mediated hepatic apoptosis partially contributes to steatotic liver I/R injury.

Given the activation of caspase-8 in steatotic liver I/R injury, we next generated hepatocyte-specific *Casp8*-knockout mice by crossing *Casp8*^fl/fl^ mice with Albumin-Cre (*Alb-Cre*) Tg mice to elucidate the function of caspase-8 in hepatocyte apoptosis and steatotic liver I/R injury. While global *Casp8* knockout leads to embryonic lethality due to the activation of RIPK3-mediated necroptosis (29), hepatocyte-specific *Casp8* knockout did not cause notable lesions or impact steatosis of steatotic liver (Supplemental Figure 3, A and B), which is consistent with the absence of RIPK3 in hepatocytes (16). We observed that hepatocyte-specific *Casp8* knockout substantially decreased hepatic apoptosis in the livers of HFD- and CDHFD-fed mice after I/R (Supplemental Figure 3, C and D). However, hepatic caspase-8 deficiency only slightly decreased the liver injury (Supplemental Figure 3, E–G) and had minor effect on the inflammation (Supplemental Figure 3, H and I) of HFD- and CDHFD-fed mice after I/R, suggesting that caspase-8–mediated apoptosis partially contributes to liver injury during steatotic liver I/R. To consolidate these observations, we then intravenously injected rats with adeno-associated virus 8 (AAV8), a liver-targeted therapeutic gene vector that has high transduction efficiency in liver cells (30), to knockdown *Casp8* in the livers of rats and assessed the effect of *Casp8* knockdown on apoptosis and liver injury during steatotic liver transplantation. We found that *Casp8* knockdown in rat livers did not affect steatosis (Supplemental Figure 4A), while knockdown did substantially reduce hepatic apoptosis in the livers of CDHFD-fed rats after OLT (Supplemental Figure 4, B and C). Similarly, *Casp8* knockdown in rat livers slightly reduced steatotic liver injury (Supplemental Figure 4, D–F) and had no effect on inflammation of recipients of steatotic donor livers after OLT (Supplemental Figure 4, G and H). Finally, PMH isolated from the liver of HFD- or CDHFD-fed *Casp8*^fl/fl^;*Alb-Cre* mice showed blockade of apoptosis after H/R challenge (Supplemental Figure 4, I and J). Interestingly, *Casp8* deficiency did not reduce but rather upregulated the expression of proinflammatory cytokines and chemokines in H/R-challenged hepatocytes (Supplemental Figure 4K).

### RIPK1 drives inflammation and caspase-8–mediated apoptosis, contributing to steatotic liver I/R injury.

**Figure 2:**
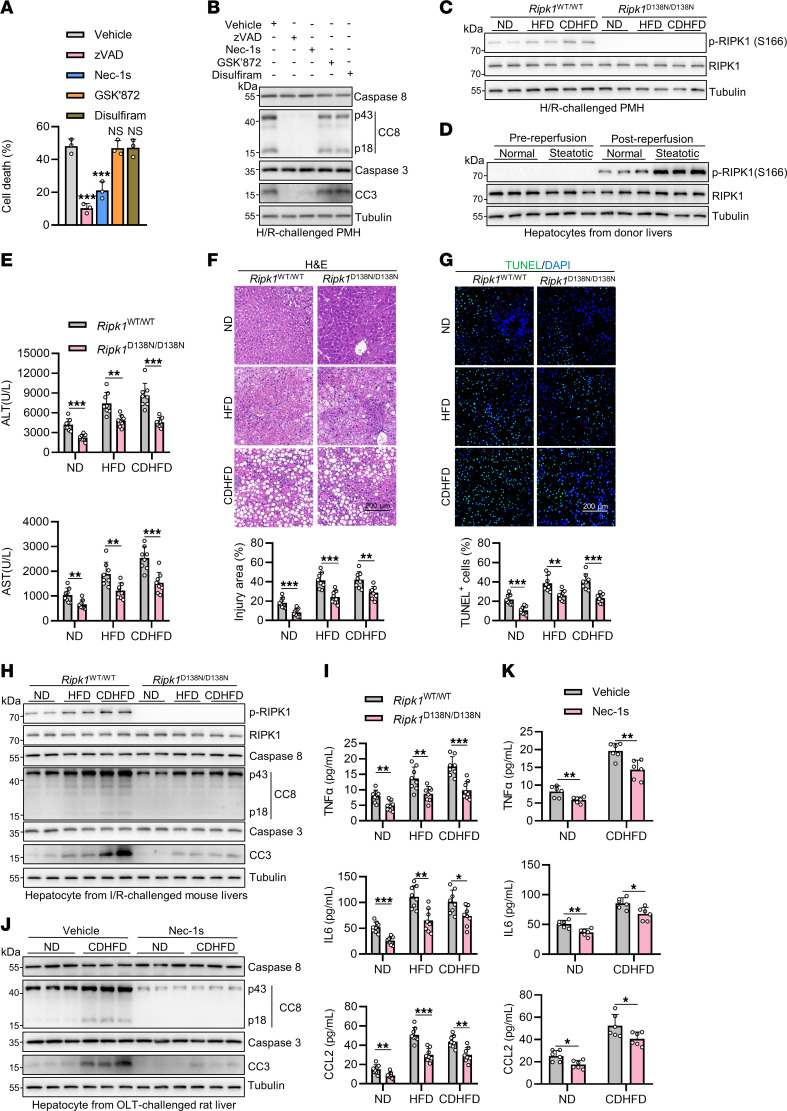
RIPK1 drives inflammation and caspase-8–mediated apoptosis, contributing to steatotic liver I/R injury. (**A** and **B**) Hepatocytes were isolated from CDHFD-fed WT mice (*n* = 3) and exposed to 10 hours hypoxia after 1 hour pretreatment of z-VAD-fmk (zVAD, 10 μM), Nec-1s (10 μM), GSK′872 (1 μM), and disulfiram (50 μM). Cell death was detected after 24 hours reoxygenation (**A**) and cell death markers were detected after 3 hours reoxygenation (**B**). (**C**) PMH isolated from ND-, HFD-, and CDHFD-fed *Ripk1*^WT/WT^ or *Ripk1*^D138N/^ ^D138N^ mice (*n* = 3) were exposed to 10 hours hypoxia. Cells were analyzed with immunoblot for p-RIPK1 (S166) after 3 hours reoxygenation. (**D**) Expression of activated RIPK1 of whole cell lysates in hepatocytes of prereperfusion or postreperfusion donor livers were detected with immunoblot (*n* = 6). (**E**–**I**) ND-, HFD-, CDHFD-fed *Ripk1*^WT/WT^ or *Ripk1*^D138N/D138N^ mice (*n* = 8) underwent 1 hour ischemia/6 hours reperfusion operation. Serum ALT/AST detection (**E**), H&E staining (**F**), TUNEL staining (**G**), cell death analysis (**H**), and detection of serum cytokine concentrations (**I**) were performed. (**J** and **K**) Livers of ND- or CDHFD-fed rats (*n* = 6) were perfused with and stored in 4°C University of Wisconsin solution supplemented with Nec-1s to a final concentration of 10 μM. After 18 hours cold storage/6 hours reperfusion, cell death analysis (**J**) and detection of serum cytokine concentrations (**K**) were performed. All data are presented as the mean ± SD. *P < 0.05, **P < 0.01, ***P < 0.001. 1-way ANOVA, post hoc Dunnett’s test (**A**). 2-way ANOVA, post hoc Bonferroni’s test (**E**–**G, I**, and **K**). Scale bars: 200 μm.

PMH isolated from the liver of CDHFD-fed mice exhibit increased sensitivity to H/R-induced apoptosis (Supplemental Figure 2, G and H), which, as expected, was blocked by the pancaspase inhibitor, z-VAD-fmk (zVAD) (31) (Figure 2, A and B). In contrast, RIPK3 inhibitor GSK′872 (32) and GSDMD inhibitor disulfiram (33), which effectively suppressed necroptosis and pyroptosis, respectively (Supplemental Figure 5A), did not affect the cell death induced by H/R (Figure 2A). Interestingly, we found that necrostain-1s (Nec-1s), a specific RIPK1 kinase inhibitor (34), substantially reduced H/R-induced apoptosis of hepatocytes isolated from steatotic liver (Figure 2, A and B), suggesting that RIPK1 kinase is activated, which may drive caspase-8–mediated apoptosis in steatotic liver I/R. Consistent with this notion, we observed elevated levels of p-RIPK1(S166), an activation biomarker of RIPK1 (35), in hepatocytes isolated from steatotic liver after H/R treatment, which was completely prevented by genetic inactivation of RIPK1 by kinase-dead D138N knockin mutation (36) (Figure 2C). Importantly, we observed increased RIPK1 activation in hepatocytes isolated from human steatotic donor livers after reperfusion and mouse steatotic livers after I/R when compared with those from respective normal control livers (Figure 2D and Supplemental Figure 5B). Further, RIPK1 activation was also higher in hepatocytes isolated from rat steatotic livers after OLT than that from normal livers after OLT, which was completely blocked by Nec-1s (Supplemental Figure 5C).

RIPK1 is a key mediator of cell death and inflammation (37). Activated RIPK1 may mediate RIPK3 and MLKL-dependent necroptosis or caspase-8–dependent apoptosis upon stimulation of tumor necrosis factor receptor 1 (TNFR1) by TNF-α (38). Given the absence of necroptosis in steatotic liver I/R, we next examined whether RIPK1 hyperactivation contributed to inflammation and caspase-8–mediated apoptosis, leading to liver injury in steatotic liver I/R. Strikingly, we observed substantial reduction of liver injury in steatotic livers of RIPK1 kinase-dead D138N knockin mice after I/R when compared with that of WT mice (Figure 2, E–G). RIPK1 inhibition had no effect on steatosis of steatotic livers (Supplemental Figure 5D) — consistent with previous studies (15) — but significantly decreased hepatic apoptosis (Figure 2H and Supplemental Figure 5E) and inflammation (Figure 2I and Supplemental Figure 5F) in both normal and steatotic livers after I/R. Moreover, in a rat model of OLT, administration of Nec-1s markedly reduced steatotic liver injury (Supplemental Figure 5, G–I). Consistently, hepatic apoptosis and inflammation are also decreased by Nec-1s administration in this setting (Figure 2, J and K and Supplemental Figure 5, J and K).

In our cellular systems, inhibition of H/R-induced apoptosis by zVAD did not decrease but rather upregulated the expression of proinflammatory cytokines and chemokines (Supplemental Figure 5L), which is consistent with the in vivo observations that *Casp8* deficiency did not prevent the inflammation in steatotic liver I/R (Supplemental Figure 3I). However, inhibition of RIPK1 by Nec-1s substantially reduced both apoptosis and inflammation in H/R-treated hepatocytes isolated from steatotic liver (Figure 2, A and B and Supplemental Figure 5L), suggesting that RIPK1 kinase-induced inflammation in steatotic liver I/R is independent of caspase-8–mediated apoptosis, which is consistent with a recent study showing that activated RIPK1 can drive inflammation in a cell death–independent manner (39). Thus, RIPK1 is hyperactivated in steatotic liver I/R, which drives both apoptosis and apoptosis-independent inflammation, contributing to steatotic liver I/R injury.

### RIPK1 activation is not mediated by TNF-α signaling in steatotic liver I/R.

The TNF-α signaling pathway is the most extensively characterized signal transduction process that regulates RIPK1 functions (37). Given the enhanced upregulation of TNF-α during steatotic liver I/R (Figure 1D, Supplemental Figure 1K, and Supplemental Figure 2, F and I), we next examined whether the ablation of *Tnf* (encoding TNF-α) could prevent RIPK1 activation and steatotic liver I/R injury. Interestingly, we found that knockout of *Tnf*, which resulted in the prevention of TNF-α production (Supplemental Figure 6A), had no impact on RIPK1 activation in hepatocytes isolated from steatotic liver after I/R (Supplemental Figure 6B). Consequently, *Tnf* deficiency did not affect liver injury or hepatic apoptosis and inflammation in steatotic liver I/R (Supplemental Figure 6, A–G). In a rat OLT model, silencing of TNFR1, the receptor of TNF-α, in hepatocytes via AAV8-mediated knockdown strategy also had no effect on liver injury, hepatic apoptosis, and inflammation of steatotic liver (Supplemental Figure 7, A–G). Additionally, primary hepatocytes isolated from steatotic liver of *Tnf*^–/–^ mice exhibited similar sensitivity to H/R-induced apoptosis and inflammatory response to that of *Tnf*^+/+^ mice (Supplemental Figure 7, H–J). Of note, TNF or TNFR1 deficiency had no impact on RIPK1 activation and liver injury in normal liver I/R (Supplemental Figure 6B and Supplemental Figure 7, D and I), which is consistent with a recent study showing that the TNF-α signaling pathway was not involved in driving RIPK1 activation in liver I/R injury (36).

### RIPK1 is activated through ZBP1 in steatotic liver I/R.

**Figure 3:**
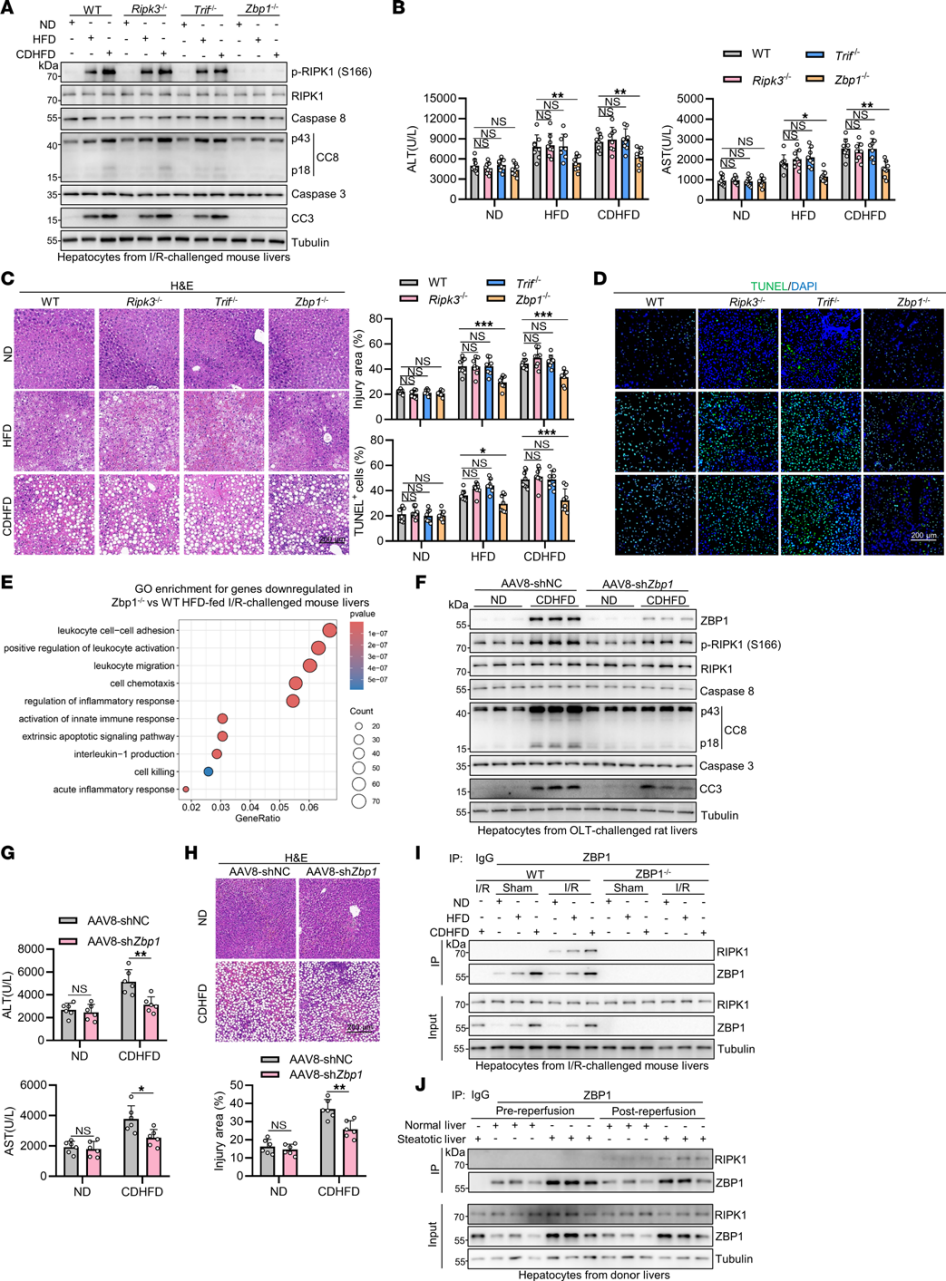
RIPK1 is activated by ZBP1 in steatotic liver I/R injury. (**A**–**E**) ND-, HFD-, CDHFD-fed WT, *Ripk3*^–/–^, *Trif*^–/–^, *Zbp1*^–/–^ mice (*n* = 8) underwent 1 h ischemia/6 h reperfusion operation. Cell death analysis (**A**), serum ALT/AST detection (**B**), H&E staining (**C**), and TUNEL staining (**D**) were performed. (**E**) Transcriptome profiling of the WT and *Zbp1*^–/–^livers (*n* = 3) was conducted. Differentially expressed genes were filtered with the criteria *P*_adj_ < 0.05 and |log_2_ FC| > 1. The genes downregulated in *Zbp1*^–/–^ livers were enriched for GO enrichment analysis. (**F**–**H**) After AAV8-mediated *Zbp1* knockdown, ND- or CDHFD-fed rat livers (*n* = 6) underwent 18 h cold storage/6 h reperfusion. Cell death analysis (**F**), serum ALT/AST detection (**G**), H&E staining (**H**) were performed. (**I** and **J**) Interaction of ZBP1 and RIPK1 was analyzed in hepatocytes of I/R-challenged mouse livers (**I**) and postreperfusion donor livers (**J**). All data are presented as the mean ± SD. *P < 0.05, **P < 0.01, ***P < 0.001. 2-way ANOVA, post hoc Bonferroni’s test. Scale bars: 200 μm.

RIPK1 contains a receptor-interacting protein homotypic interaction motif (RHIM), which is involved in mediating its interaction with other RHIM-containing proteins, including RIPK3, TRIF, and ZBP1 (40). These RHIM-mediated interactions may generate a scaffold, enabling RIPK1 activation in certain conditions (40). RIPK3 usually acts as a downstream mediator of RIPK1 in promoting necroptosis, it can also induce RIPK1 activation via RHIM-mediated interactions to promote apoptosis in certain conditions (41). TRIF has been shown to promote RIPK1 activation via RHIM-mediated interactions in lipopolysaccharide-induced (LPS-induced) necroptosis when caspase-8 is inhibited (42). ZBP1 can also act as an upstream mediator of RIPK1 via RHIM-mediated interactions to induce apoptosis (43). We next examined whether RIPK1 activation in steatotic liver I/R is mediated by these RHIM-containing proteins. We found that knockout of *Zbp1*, but not *Ripk3* or *Trif*, substantially reduced RIPK1 activation in hepatocytes isolated from the livers of I/R-challenged HFD- or CDHFD-fed mice (Figure 3A). In addition, there was diminished RIPK1 activation and reduced cell death and inflammatory response in H/R-treated steatotic *Zbp1*^–/–^ PMH compared with *Zbp1*^+/+^ PMH (Supplemental Figure 8, A–C). Thus, hepatic RIPK1 is activated through ZBP1 during steatotic liver I/R.

### ZBP1 specifically promotes steatotic liver I/R injury.

We next assessed the role of ZBP1 in steatotic liver I/R injury. Knockout of *Ripk3*, *Trif*, and *Zbp1* did not affect the degree of steatosis in the liver of HFD- or CDHFD-fed mice (Supplemental Figure 8D). Consistent with the role of ZBP1 in driving RIPK1 activation, *Zbp1* knockout significantly protected against I/R injury in HFD- or CDHFD-fed mice (Figure 3, B–D). Interestingly, *Zbp1* knockout had no markable effect on I/R injury of ND-fed mice (Figure 3, B–D). By contrast, knockout of *Ripk3* or *Trif* did not confer any protection against I/R injury in any group (Figure 3, B–D). Further, deficiency in *Zbp1*, but not *Ripk3* or *Trif*, substantially reduced steatotic liver apoptosis and inflammation (Figure 3A and Supplemental Figure 8, E–G). Transcriptome profiling of HFD-fed I/R-challenged mouse livers revealed downregulation of numerous proinflammatory genes upon *Zbp1* knockout (Supplemental Figure 9, A and B), with multiple inflammatory and cell death–related pathways significantly enriched (Figure 3E and Supplemental Figure 9C). To consolidate these observations, we further used the rat OLT model to mimic clinical liver transplantation. We found that AAV8-mediated *Zbp1* knockdown in the steatotic liver also suppressed hepatic RIPK1 activation (Figure 3F) without affecting steatosis (Supplemental Figure 9D). Consequently, steatotic liver injury after OLT was significantly reduced upon *Zbp1* knockdown (Figure 3, G and H and Supplemental Figure 9E). Additionally, hepatic apoptosis (Figure 3F and Supplemental Figure 9F) and inflammation (Supplemental Figure 9, G and H) in recipient rats were also decreased in ZBP1-knockdown steatotic livers compared with control steatotic livers. Consistently, ZBP1 knockdown did not affect the outcomes of liver injury in normal liver after OLT (Figure 3, F–H and Supplemental Figure 9, E–H). Thus, ZBP1 specifically exacerbated liver injury in steatotic liver I/R.

Next, we assessed the interaction of ZBP1 and RIPK1 during steatotic liver I/R. RIPK1 was coimmunoprecipitated with ZBP1 in *Zbp1*^+/+^ steatotic hepatocytes after I/R but not in the sham condition, which was abolished in *Zbp1*^–/–^ steatotic hepatocytes (Figure 3I), suggesting that an I/R-stimulated event triggered ZBP1 activation, leading to the interaction of ZBP1 and RIPK1 in steatotic liver I/R. In line with this notion, we detected the interaction of ZBP1 and RIPK1 only after reperfusion in human steatotic donor livers (Figure 3J). Thus, ZBP1 mediates the activation of RIPK1 in I/R to aggravate steatotic liver I/R injury.

### ZBP1 is increased in steatotic livers, underlying its specificity in promoting steatotic liver I/R injury.

**Figure 4:**
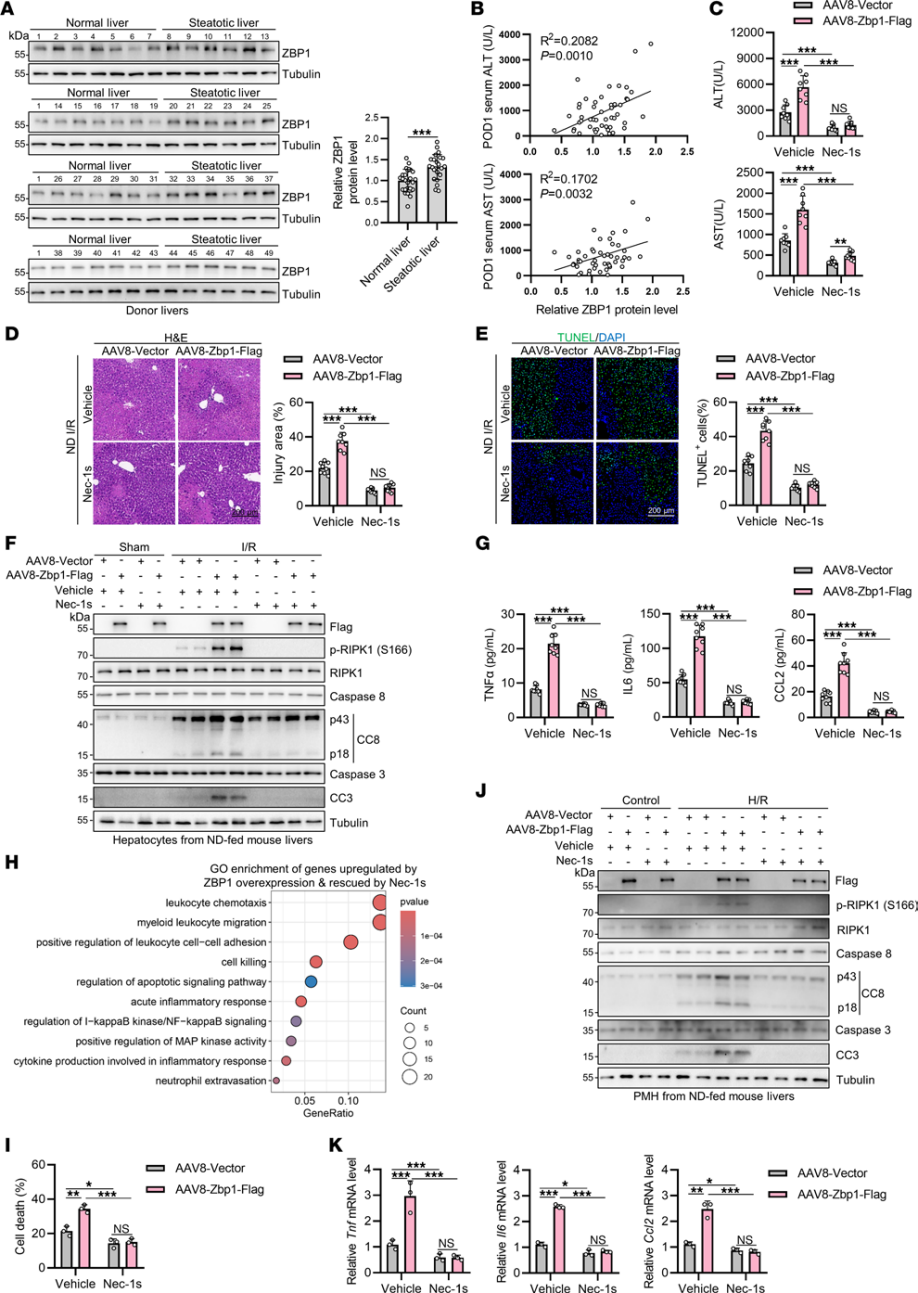
ZBP1 is increased in steatotic livers, underlying its specificity in promoting steatotic liver I/R injury. (**A**) ZBP1 protein levels were detected in normal and steatotic donor livers after reperfusion. ZBP1 protein levels relative to tubulin were compared between normal donor livers and steatotic donor livers. *n* = 25 for normal donor livers and *n* = 24 for steatotic donor livers. (**B**) The correlation between ZBP1 protein level and recipients’ serum ALT and AST levels in the POD1 was analyzed (*n* = 49). (**C**–**H**) After AAV8-mediated Zbp1 overexpression and Nec-1s pretreatment, ND-fed mice (*n* = 8) were subjected to I/R challenge. Serum ALT/AST detection (**C**), H&E staining (**D**), TUNEL staining (**E**), cell death analysis (**F**), and detection of serum cytokine concentrations (**G**) were performed. (**H**) Transcriptome profiling of the mouse livers was conducted. The expression of genes that were upregulated by ZBP1 overexpression and rescued by Nec-1s pretreatment were analyzed for GO enrichment. (**I**–**K**) PMH isolated from AAV-mediated Zbp1-overexpression normal mouse livers (*n* = 3) were pretreated with Nec-1s and exposed to H/R challenge. Cell death (**I**), apoptosis (**J**), mRNA levels of cytokines (**K**) and were analyzed. All data are presented as the mean ± SD. *P < 0.05, **P < 0.01, ***P < 0.001. Unpaired 2-tailed Student’s *t* test (**A**). Pearson’s correlation test (**B**). 2-way ANOVA, post hoc Bonferroni’s test (**C**–**E**, **G**, **I**, and **K**). Scale bars: 200 μm.

We noticed higher expression levels of ZBP1 in hepatocytes from both human and mouse steatotic livers than in normal livers (Figure 3, I and J), which might underlie the specificity of ZBP1 in contributing to RIPK1-mediated liver injury of steatotic liver I/R. Consistent with this notion, we observed positive correlations between liver ZBP1 protein levels and liver damage of patients in the first day post liver transplantation (POD1) (Figure 4, A and B and Supplemental Figure 10A). Further, liver ZBP1 protein levels were positively correlated with hepatic apoptosis and inflammation in recipients of donor livers (Supplemental Figure 10, B–F). The incidence of sinusoidal congestion was also higher in patients with donor livers expressing high levels of ZBP1 than in those expressing low ZBP1 levels (Supplemental Figure 10G).

To further assess the role of ZBP1 in liver I/R injury, we specifically overexpressed ZBP1 in normal livers of ND-fed mice using AAV8-mediated delivery of ZBP1 cDNA driven by the TBG promoter. No abnormalities were observed in livers after sham operation, excluding the possibility that excessive doses of ZBP1 may induce I/R-independent spontaneous cell death (44) (Supplemental Figure 10H). However, ZBP1 overexpression resulted in increased liver damage after I/R (Figure 4, C–E). We also observed substantial RIPK1 activation in ZBP1-overexpressing hepatocytes upon I/R (Figure 4F). Consequently, hepatic apoptosis (Figure 4F and Supplemental Figure 10I) and inflammation (Figure 4G and Supplemental Figure 10J) were upregulated by overexpressing ZBP1 in the liver after I/R. Transcriptome profiling further confirmed that ZBP1 overexpression stimulated the expression of various genes associated with inflammation and cell death (Figure 4H and Supplemental Figure 10, K and L). In all of the above experiments, inhibition of RIPK1 with Nec-1s prevented hepatic apoptosis, inflammation, and liver damage in I/R-challenged ZBP1-overexpressing livers (Figure 4, C–H and Supplemental Figure 10, I–L), suggesting that RIPK1 is indispensable for ZBP1-mediated liver injury in I/R. Consistent with the in vivo findings, PMH isolated from ZBP1-overexpressing mice showed higher levels of RIPK1 activation and higher sensitivity to H/R-induced apoptosis and inflammation than that from control mice, in which activation and sensitivity was suppressed by Nec-1s (Figure 4, I–K). Hence, ZBP1 is increased in steatotic livers, which promotes RIPK1-mediated liver injury in steatotic liver I/R.

### ZBP1 is induced through PA-activated JNK signaling in steatotic liver.

**Figure 5:**
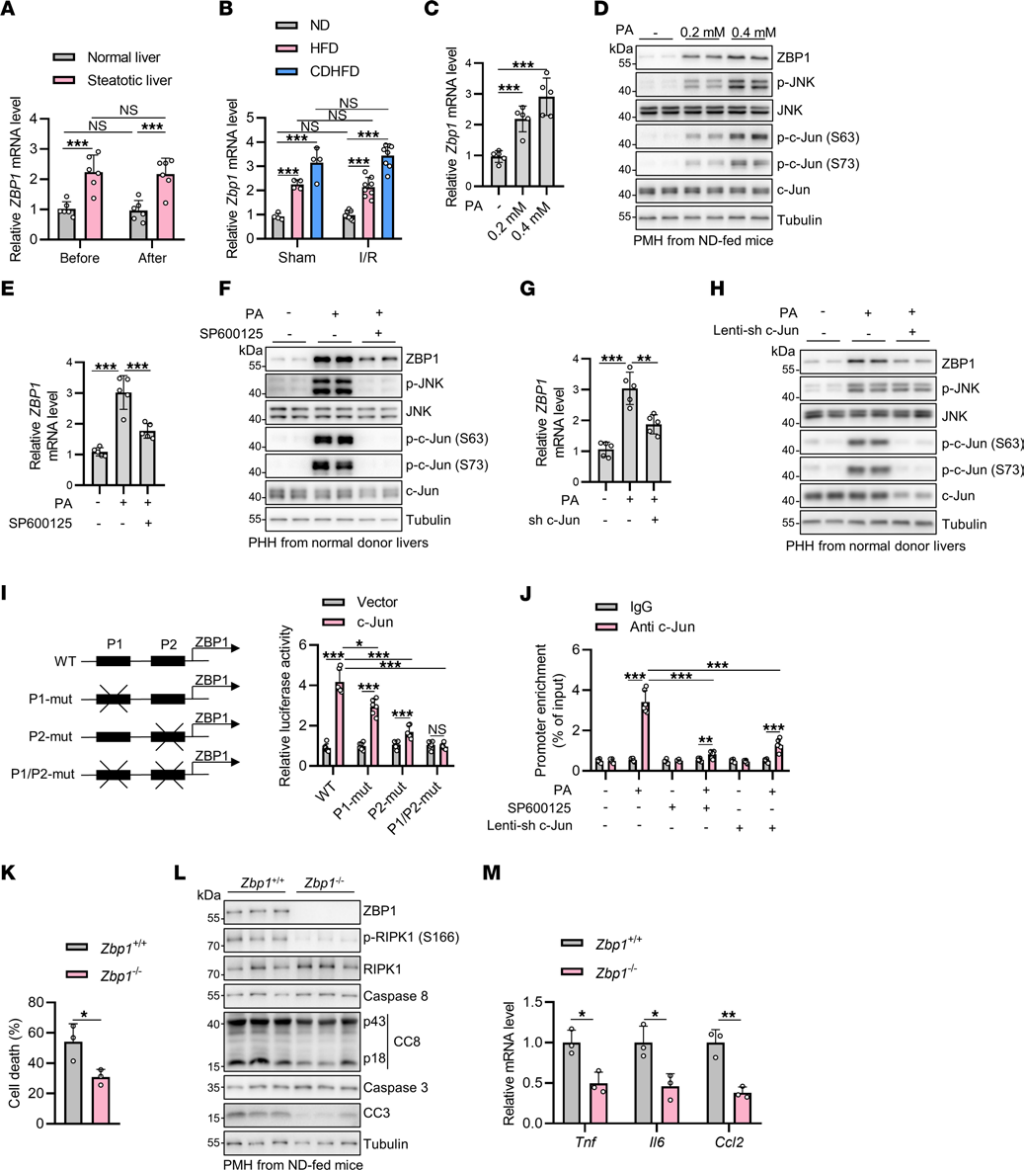
Palmitic acid upregulates hepatocyte ZBP1 expression level through JNK pathway to exacerbate I/R injury of steatotic livers. (**A** and **B**) *ZBP1* mRNA levels in donor livers (*n* = 6) or mouse I/R model (*n* = 4 for sham and *n* = 8 for I/R) were quantified. (**C** and **D**) mRNA and protein levels of ZBP1 after different concentration of PA in normal PMH for 48 hours (*n* = 5). (**E** and **F**) mRNA and protein levels of ZBP1 after stimulation of 0.4 mM PA and 10μM SP600125 in normal PHH for 24 hours (*n* = 5). (**G** and **H**) mRNA and protein levels of ZBP1 after stimulation of 0.4 mM PA for 24 hours in c-Jun knockdown normal PHH (*n* = 5). (**I**) *ZBP1* promoter mutation schema and luciferase activity after c-Jun overexpression (*n* = 6). (**J**) Binding of c-Jun to *ZBP1* promoter after stimulation of PA and SP600125 treatment or c-Jun knockdown in normal PHH (*n* = 6). (**K**–**M**) *Zbp1*^+/+^ or *Zbp1*^–/–^ PMH (*n* = 3) were pretreated with 0.2 mM PA for 24 hours and subjected to 10 h hypoxia. Cell death (**K**) was analyzed after 24 hours reoxygenation and apoptosis (**L**) and mRNA levels of cytokines (**M**) was analyzed after 3 hours reoxygenation. All data are presented as the mean ± SD. *P < 0.05, **P < 0.01, ***P < 0.001. 2-way ANOVA, post hoc Bonferroni’s test (**A**, **B**, **I**, **J**, and **M**). 1-way ANOVA, post hoc Dunnett’s test (**C**, **E**, and **G**). Unpaired, 2-tailed Student’s *t* test (**K**).

Next, we investigated the mechanism underlying the upregulation of ZBP1 in steatotic liver. The mRNA levels of ZBP1 were significantly increased in both human and mouse steatotic livers compared with respective normal control livers, which were not affected after I/R (Figure 5, A and B). The upregulated *ZBP1* transcription in steatotic livers was further validated in 2 Gene Expression Omnibus (GEO) data sets (Supplemental Figure 11, A and B), and ZBP1 mRNA levels were positively correlated with the nonalcoholic fatty liver disease (NAFLD) activity score (Supplemental Figure 11B) (45, 46). Given that the increase in ZBP1 expression was likely mediated by transcriptional activation, we employed the bioinformatics tool JASPAR to analyze the promoter region of ZBP1 and identified 2 predicted binding sites for c-Jun (47), which is activated by c-Jun N-terminal kinase (JNK) in steatotic liver and acts as a central mechanism underlying the lipotoxic effects of excessive PA in MASLD (48, 49). We thus hypothesized that PA-activated JNK signaling might activate the transcription of *ZBP1* in steatotic livers. Consistent with previous studies (50, 51), we observed PA accumulation as determined by ultra performance liquid chromatography-tandem mass spectrometry, as well as JNK pathway activation as determined by the phosphorylation of JNK and c-Jun, in steatotic livers (Supplemental Figure 11, C–E). Treatment with PA in primary hepatocytes, which mimics in vivo steatotic pathologies (52), led to a time- and dose-dependent upregulation of both ZBP1 mRNA and protein levels (Figure 5, C and D and Supplemental Figure 11, F and G). Inhibition of JNK with the specific inhibitor SP600125 (53) substantially compromised the PA-induced increase of ZBP1 levels in both PMH and PHH (Figure 5, E and F and Supplemental Figure 11, H and I). Additionally, knockdown of c-Jun through short hairpin RNA (shRNA) also decreased PA-induced upregulation of ZBP1 levels in PHH (Figure 5, G and H).

To directly associate c-Jun and *ZBP1* transcription, we mutated the 2 putative c-Jun binding sites in ZBP1 promoter and performed a luciferase reporter assay to measure the specific ability of c-Jun to bind to ZBP1 promoter. We found that overexpression of c-Jun was able to activate the transcription of ZBP1, while mutating both the putative binding sites in ZBP1 promoter completely abrogated this effect (Figure 5I). Moreover, PA stimulation significantly enhanced c-Jun binding to *ZBP1* promoter in PHH, which was compromised by SP600125 treatment or c-Jun knockdown (Figure 5J). To simulate steatotic liver I/R injury in vitro, we treated both PMH and PHH under PA challenge with H/R. PA treatment substantially increased RIPK1 activation, apoptosis, and inflammation after H/R (Supplemental Figure 12, A–F), which were mitigated by JNK inhibition or ZBP1 depletion (Figure 5, K–M and Supplemental Figure 12, A–I). Thus, ZBP1 was upregulated through PA-activated JNK signaling in steatotic livers, which specifically exacerbates liver injury of steatotic livers during I/R.

### Z-NA sensing is not required for ZBP1-induced liver I/R injury.

**Figure 6:**
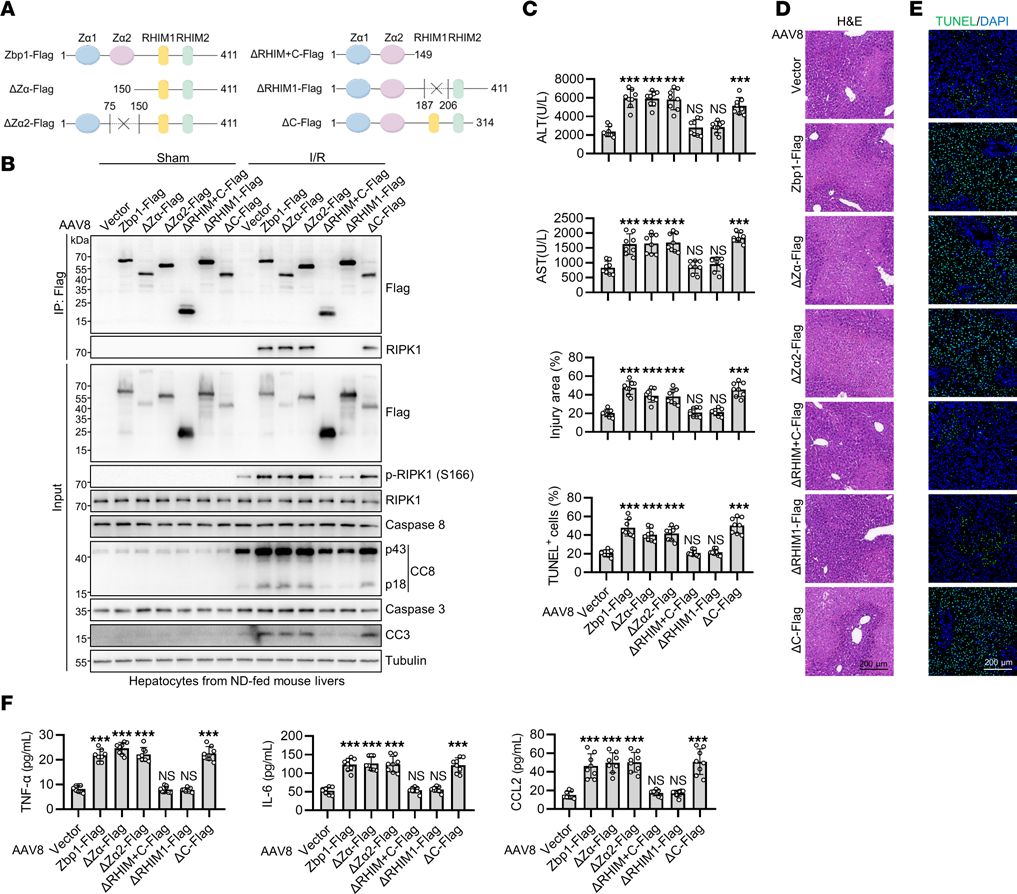
Z-NA sensing is not required for ZBP1-induced liver I/R injury. (**A**–**F**) Different truncation mutants of Zbp1 (**A**) were packaged into AAV8 and injected to ND-fed mice and I/R operation was conducted 1 month later (*n* = 8). Cell death and ZBP1-RIPK1 interaction analysis (**B**), serum ALT/AST detection (**C**), H&E staining (**D**), TUNEL staining (**E**), and detection of serum levels of the proinflammatory cytokines (**F**) were performed. All data are presented as the mean ± SD. *P < 0.05, **P < 0.01, ***P < 0.001. 1-way ANOVA, post hoc Dunnett’s test. Scale bars: 200 μm.

Expression of ZBP1 by itself is insufficient to induce cell death and liver damage (Supplemental Figure 10H), and RIPK1 only interacts with ZBP1 in steatotic hepatocytes after I/R, but not in the sham condition (Figure 3, I and J), suggesting that an I/R-stimulated event triggered ZBP1 activation. ZBP1 is activated by virus-derived or endogenous Z-NA during viral infection and disease conditions through its Zα domain (54). We generated AAV8 vectors encoding full-length ZBP1 or its truncation mutants that either lacked the Zα or Zα2 domain (ΔZα or ΔZα2) (Figure 6A) and injected them to ND-fed mice. Expressing these ZBP1 truncation mutants by itself did not induce any notable alterations in mouse livers (Supplemental Figure 13A). However, I/R still induced a ZBP1-RIPK1 interaction, RIPK1 phosphorylation, hepatic apoptosis, liver damage, and inflammation in mouse livers (Figure 6, B–F and Supplemental Figure 13, B and C). ZBP1 contains a C-terminal domain and 2 RHIM domains (40). Interestingly, RHIM1 but not the C-terminal domain was indispensable for I/R-induced ZBP1-RIPK1 interaction, RIPK1 activation, and liver damage (Figure 6, A–F and Supplemental Figure 13, B and C). These findings suggest that ZBP1 is activated through its RHIM domain independent of the canonical Z-NA sensing.

### ZBP1 is activated through ROS-induced aggregation in liver I/R.

**Figure 7:**
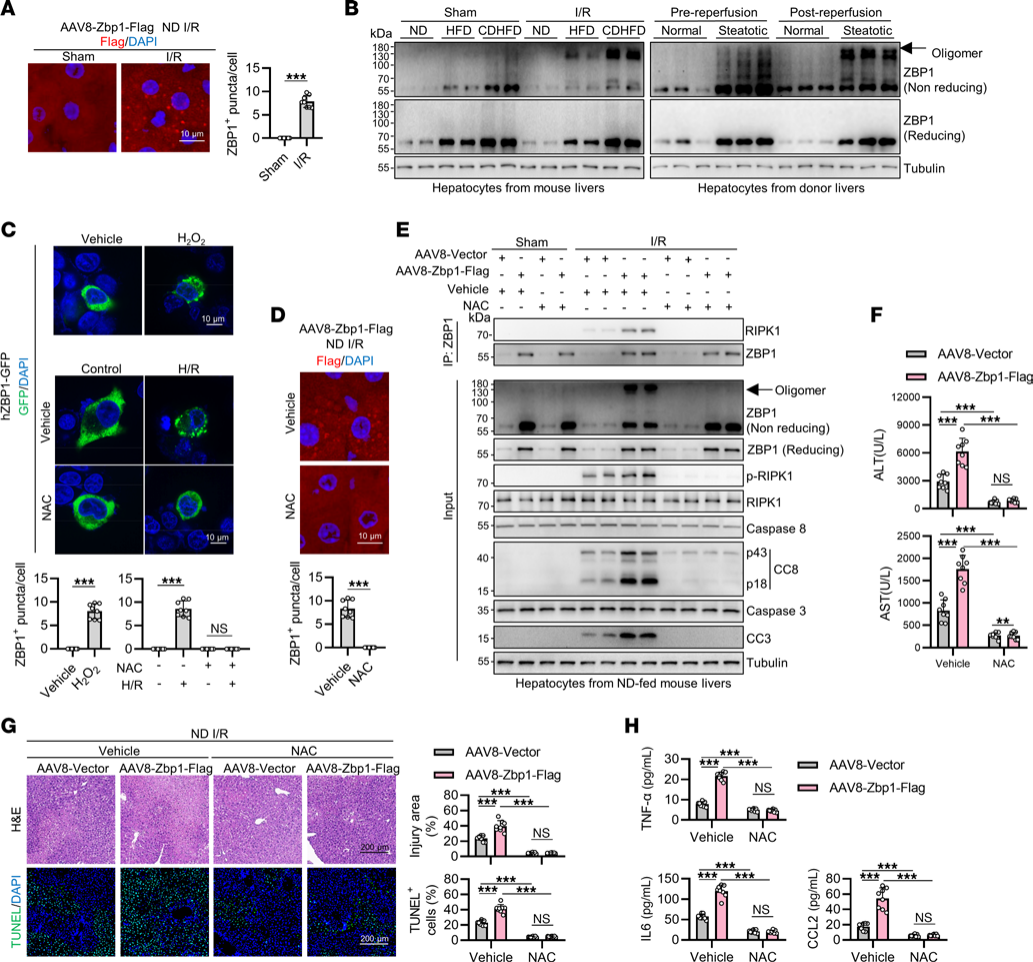
ROS triggers ZBP1 aggregation and activation in liver I/R injury. (**A**) Flag immunostaining was performed in I/R-challenged ND-fed mouse livers overexpressing Zbp1-Flag (*n* = 8). (**B**) ZBP1 aggregation was detected with immunoblot under nonreducing or reducing conditions in I/R-challenged mouse livers or transplantation-challenged donor livers (*n* = 3). (**C**) hZBP1-GFP plasmids were transfected into HEK293T cells. After 1 mM H_2_O_2_ treatment for 4 hours or H/R challenge after pretreatment with 10 mM NAC, ZBP1 oligomers were detected from green fluorescence (*n* = 8). (**D**–**H**) After AAV8-mediated Zbp1-Flag overexpression in ND-fed mice and pretreatment with NAC, I/R operation was performed (*n* = 8). Flag immunostaining (**D**), ZBP1 aggregation and ZBP1-RIPK1 interaction (**E**), serum ALT/AST levels (**F**), H&E and TUNEL staining (**G**), and detection of serum levels of the proinflammatory cytokines (**H**) were detected. All data are presented as the mean ± SD. *P < 0.05, **P < 0.01, ***P < 0.001. Unpaired, 2-tailed Student’s *t* test (**A** and **D**). 2-way ANOVA, post hoc Bonferroni’s test (**C**, **G**, and **H**). Scale bars (**A**, **C**, and **D**): 10 μm, (**G**) 200 μm.

The formation of higher-order supramolecular complexes through aggregation is usually required for the activation of cell death-inducing proteins, including ZBP1 (55). In mouse livers overexpressing ZBP1, we observed ZBP1 punctate distribution, indicative of protein aggregation, in I/R but not in sham conditions (Figure 7A). Immunoblots under nonreducing conditions further revealed the aggregation of endogenous ZBP1 in I/R-stimulated steatotic mouse livers and human steatotic donor livers after reperfusion (Figure 7B). ROS have been implicated in promoting aggregation of cell death-inducing proteins (56). Because ROS also acts as an important driving factor contributing to I/R injury (57, 58), we hypothesized that ROS may trigger ZBP1 aggregation and activation in liver I/R. Consistent with this hypothesis, in HEK293T cells expressing human ZBP1 and GFP fusion protein (hZBP1-GFP), treatment with H_2_O_2_ was sufficient to induce the aggregation of ZBP1 (Figure 7C). Further, H/R challenge also stimulated ZBP1 aggregation, which was effectively blocked by treatment with N-acetylcysteine (NAC), a ROS scavenger (Figure 7C). In mouse livers expressing ZBP1, NAC treatment also abolished ZBP1 aggregation induced by I/R (Figure 7, D and E), resulting in substantial reduction of ZBP1-RIPK1 interaction, RIPK1 activation, hepatic apoptosis, inflammation, and liver damage (Figure 7, E–H and Supplemental Figure 13D). However, ZBP1 aggregation was unaffected by α-tocopherol, a membrane-located lipid ROS scavenger (Supplemental Figure 13, E–G), suggesting that cytosolic ROS play the principal role in promoting ZBP1 aggregation. Thus, ZBP1 is activated through ROS-induced aggregation in liver I/R to promote RIPK1-mediated apoptosis and inflammation, contributing to steatotic liver I/R injury.

## Discussion

This study reveals an important mechanism underlying the susceptibility of steatotic livers to I/R injury. We found that the expression level of ZBP1 is significantly increased in steatotic livers through PA-activated JNK signaling. During liver transplantation, the inevitable I/R injury results in an excessive production of ROS, which facilitates the aggregation and activation of ZBP1. Subsequently, via RHIM domain–mediated interactions, ZBP1 recruits and facilitates RIPK1 activation, which exacerbates caspase-8–mediated apoptosis and apoptosis-independent inflammation, leading to enhanced liver damage induced by immune cell attack. Thus, ZBP1 plays an important role in contributing to the increased susceptibility of steatotic livers to I/R injury.

Hepatocyte death is a pivotal pathological process in liver I/R injury. Hepatic apoptosis is exacerbated in steatotic livers after I/R injury, leading us to speculate that blocking apoptosis may provide protection against liver I/R injury. Our genetic evidences suggest that hepatic apoptosis is mediated partially by caspase-8. However, knockout of *Casp8* in the liver substantially reduces hepatic apoptosis but provides limited effects on the outcomes of steatotic liver I/R injury. Indeed, *Casp8* deficiency led to enhanced inflammation in steatotic liver I/R, presumably due to the blockade of the inhibitory role of caspase-8 on RIPK1 activation via cleavage (39). Thus, caspase-8–mediated apoptosis only partially contributes to hepatic cell death in steatotic liver I/R injury. Notably, the pyroptosis effector N-GSDMD is not detected in hepatocytes of steatotic liver I/R injury, which is consistent with a previous study reporting that hepatocyte-specific GSDMD deficiency does not confer protection against liver I/R injury (59). The involvement of necroptosis in liver I/R injury is controversial (14, 60, 61). In our study, we did not detect necroptotic biomarkers and the essential necroptosis effector RIPK3, in hepatocytes after steatotic liver I/R injury, which is consistent with previous studies (15, 16, 36). We supposed that ferroptosis, a type of regulated necrosis mediated by iron-dependent lipid peroxidation, might account for hepatocyte death since ROS is a driving factor of both ferroptosis and I/R injury and several studies reported that ferroptosis mediates liver I/R injury (18–20). However, we found the levels of lipid peroxidation were similar between normal and steatotic livers, excluding the role of ferroptosis in contributing to the increased sensitivity of steatotic livers to I/R injury. Thus, inflammation-induced injury, such as those mediated by immune cell attack (62), may represent the main mechanism of hepatocyte death in steatotic liver I/R injury. Our results suggest that targeting the known programmed cell death pathways may not improve liver damage in steatotic liver I/R injury. A more viable strategy to alleviate steatotic I/R injury is to intervene the upstream factors that contribute to the injury, such as ZBP1 or RIPK1 found in this study, which induce both apoptosis and inflammation, contributing to steatotic liver I/R injury.

Our findings underscore the pivotal role of RIPK1 as a critical effector triggering apoptosis and apoptosis-independent inflammation in both normal and steatotic livers during I/R injury. Surprisingly, the global knockout of *Tnf* did not modulate RIPK1 activation or the severity of liver I/R injury. The observations that *Tnf* knockout or hepatocyte *Tnfr1* knockdown fails to confer protection against liver I/R injury are consistent with 2 previous studies (63, 64). In normal liver I/R, as suggested by a recent study (36), RIPK1 might be activated through death receptor 4/5 pathway. While in steatotic liver I/R, our data suggested that the increased RIPK1 activation is mediated by ZBP1. ZBP1 usually induces cell death and inflammatory response after sensing Z-NA (22). The activation mechanism of ZBP1 under conditions other than Z-NA remains a fundamental issue in the field, which may be linked to progression of certain diseases (40). Our study reveals that ROS generated during liver I/R facilitates ZBP1 aggregation and activation, presumably due to ROS-mediated disulfide bond formation, which has been observed in a previous study (65). This finding may have a significant impact on the understanding of activation mechanism of ZBP1, which might be implicated in other human diseases since ROS is frequently activated in many pathologic conditions. Our findings also suggest that ZBP1 only functions in steatotic liver I/R injury but not normal liver I/R injury, presumably due to its significantly low expression levels in normal livers. We also reveal that PA, which is linked to the pathogenesis of steatotic liver diseases (53), upregulates ZBP1 expression by activating the JNK pathway, further highlighting the importance of the JNK pathway in mediating steatotic liver I/R injury (51).

Taken together, this study reveals that ZBP1 is activated by transplantation-associated ROS to facilitate RIPK1 kinase activation specifically in steatotic donor livers, which mediates apoptosis and apoptosis-independent inflammation, leading to the exacerbation of liver injury. The findings establish a novel working model involving ZBP1-RIPK1 interaction, which could be targeted to make the best utilization of steatotic donor livers and relieve donor liver shortage.

## Methods

### Sex as a biological variable.

For clinical human donor liver samples, both sexes were examined. For animal models, only male mice or rats were examined to reduce female sexual cycle–related variation. The results were expected to be relevant for both sexes.

### Animals.

*Casp8*^fl/fl^ (S-CKO-01552), *Mlkl*^–/–^ (S-KO-14468), *Ripk3*^–/–^ (S-KO-10874), *Zbp1*^–/–^ (S-KO-11088) mice were obtained from Cyagen, China. *Trif*^–/–^ (T017208) mice were obtained from Gempharmatech. *Tnf*^–/–^ (NM-KO-00100) and *Alb-Cre* (NM-KI-220458) mice were obtained from The Shanghai Model Organisms Center. *Ripk1*^D138N/D138N^ mice were generated as previously described (36). Littermate controls were employed for these gene knockout or knockin mice. Additional WT mice were obtained from hfkbio, China. All mice were in the C57BL/6J background. WT Sprague-Dawley rats were obtained from hfkbio. For the CDHFD-induced MASLD mouse model, male mice were fed CDHFD (60% fat, 0.1% methionine, and no added choline, A06071302, Research Diet, USA) for 1 week, starting at 23 weeks of age. For the HFD-induced MASLD mouse model, male mice were fed a HFD consisting of 60% fat (D12492, Research Diet) for 16 weeks, starting at 8 weeks of age. For the CDHFD-induced MASLD rat model, male rats were fed CDHFD (A06071302, Research Diet) for 1 week, starting at 11 weeks of age. Animals were fed ND as control. All animals were housed in a specific pathogen-free environment with no more than 5 mice or 3 rats per cage. They were subjected to controlled light conditions (12 hours light and 12 hours dark cycle), temperature (24 ± 2 °C), and humidity (50 ± 10%) levels. The animals were provided with ad libitum access to food and water throughout the duration of the experiments. The study adhered to regulations concerning animal welfare. General welfare checks were conducted daily. Before each individual experiment, the animals were thoroughly evaluated for suitability based on preset criteria approved by local animal welfare authorities. Mice were euthanized by isoflurane overdose followed by cervical dislocation prior to sample harvest.

### Human liver samples.

Prereperfusion liver samples were obtained from the left lobe after cold storage before implantation, and after-reperfusion samples were obtained from the left lobe after around 2 hours of portal reperfusion before abdominal closure, respectively. Samples were fixed in formalin for pathological analysis or quickly frozen in liquid nitrogen for immunoblots and qRT-PCR analysis or stored in cold University of Wisconsin (UW) solution and transferred to laboratory for primary hepatocyte isolation. All donor livers were procured through the China Organ Transplant Response System between August, 2022 and October, 2023. Steatosis was independently diagnosed by 2 pathologists based on the criteria of macrovesicular steatosis greater-than 5%. This study enrolled 25 normal donor livers and 24 steatotic donor livers. The basic information of donors and recipients is provided in Supplemental Tables 1 and 2.

### Statistics.

All data are presented as the mean ± SD. Immunoblots were independently repeated at least 3 times, yielding consistent results. Statistical analyses utilized GraphPad Prism 8.0 (v8.4.1). The normality of samples was assessed using the Shapiro-Wilk test before statistical analysis. For normally distributed data, an unpaired, 2-tailed Student’s *t* test was employed for 2-group comparisons and a 1-way ANOVA with post hoc Dunnett’s tests for multiple group comparisons with a designated control. For data with more than 1 variable, a 2-way ANOVA with post hoc Bonferroni’s tests for multiple group comparisons was employed. In cases of nonnormal distribution, a nonparametric statistical analysis was conducted using the Mann-Whitney test for 2-group comparisons or the Kruskal-Wallis test followed by Dunnett’s test for multiple comparisons. *P* value less than 0.05 was considered significant. Statistical significance was determined at *P < 0.05, **P < 0.01, ***P < 0.001, and not significant (NS).

### Study approval.

All the animal experiments were conducted in accordance with the approved protocols by Institutional Animal Care and Use Committee of Huazhong University of Science and Technology under approval No. 2023-3548. All the procedures involving human samples were approved by the Ethics Committee of Union Hospital affiliated to Tongji Medical College, Huazhong University of Science and Technology, under approval no. UHCT-2023-0870, and adhered to the principles of Declaration of Helsinki. Written informed consent was obtained from the donors and the recipients or their families.

### Data availability.

All source data values were provided in the Supporting Data Values file. RNA-Seq data were deposited at GEO under accession number GSE254747.

All other underlying data and any supporting analytic code in this paper are available from the corresponding author upon request through email (gujinyang@hust.edu.cn).

Additional methods are provided in the Supplemental material.

## Author contributions

DX and JG designed research studies. RL, HC, SZ, MC, and TZ conducted experiments. RL, HC, SZ, MC, and TZ acquired data. RL, HC, SZ, MC, and TZ analyzed data. GW, DZ, XW, JX, SD, and TL provided reagents. RL, DX, and JG wrote the manuscript. The order of authorship was determined by overall contributions and approved by all the authors.

## Supplementary Material

Supplemental data

Unedited blot and gel images

Supporting data values

## Version 1 05/14/2024

In-Press Preview

## Version 2 07/01/2024

Electronic publication
