# Supplementary material for: ZBP1-mediated apoptosis and inflammation exacerbate steatotic liver ischemia/reperfusion injury
Source: J Clin Invest. 2024 May 14;134(13):e180451. doi: 10.1172/JCI180451 (PMC11213514; doi:10.1172/JCI180451)
Supplement: Supplemental data [file jci-134-180451-s066.pdf]

Supplemental material includes Supplemental methods, 13 Supplemental Figures and Supplemental Figure legends, 2 Supplemental Tables.

Supplemental methods

Animal metabolic dysfunction-associated steatotic liver disease (MASLD) model

For the choline-deficient high-fat-diet (CDHFD)-induced MASLD mouse model, male mice were fed CDHFD (60% fat, 0.1% methionine, and no added choline, A06071302, Research Diet) for 1 week, starting at 23 weeks of age. For the high-fat-diet (HFD)-induced MASLD mouse model, male mice were fed HFD consisting of 60% fat (D12492, Research Diet) for 16 weeks, starting at 8 weeks of age. For the CDHFD-induced MASLD rat model, male rats were fed CDHFD (A06071302, Research Diet) for 1 week, starting at 11 weeks of age. Animals were fed normal chow diet (ND) as control. All animals were housed in a specific pathogen-free environment with no more than 5 mice or 3 rats per cage and were provided with *ad libitum* access to food and water throughout the experiments. Mice were euthanized by isoflurane overdose followed by cervical dislocation prior to sample harvest.

Mouse ischemia-reperfusion (I/R) model

Mouse 70% warm I/R model was used. Male mice underwent ischemia-reperfusion (I/R) operation at 24 weeks of age. Buprenorphine was administered before surgery. Briefly, mice were anesthetized with 50 mg/kg of pentobarbital sodium and the skin was cleaned with a 70% ethanol and betadine solution. After abdomen open at midline, the left and middle branch of portal vein was ligated. After 1 h ischemia, the ligation was removed and liver reperfusion was confirmed

visually. After 6 h reperfusion, the mice were anesthetized and blood was taken from orbital vein. After liver perfusion with normal saline from portal vein, the left and middle lobe were excised. Each I/R group contains 8 mice and each sham group contains 4 mice.

Rat orthotopic liver transplantation (OLT) model

OLT model was performed on rats at 12 weeks of age. Buprenorphine was administered to donor and recipient rats before surgery. Briefly, rats were anesthetized with 50 mg/kg of pentobarbital sodium and the skin was cleaned with a 70% ethanol and betadine solution. After skeletonization of portal vein, livers were perfused with 4 °C UW solution and excised. After 18 h of cold storage, the donor livers were implanted to healthy recipient rats. After reperfusion for 6 h, the rats were anesthetized and blood was taken from inferior vena. After perfusion with normal saline from portal vein, the livers were excised. Each OLT group contains 6 rats and each sham group contains 3 rats.

Cell culture

L929, BMDM, HEK293T, primary hepatocytes were cultured in DMEM (Procell) supplemented with 10% (vol/vol) fetal bovine serum (FBS, Gibco) and 100 units/ml penicillin/streptomycin (Gibco). All cell cultures were maintained at 37 °C with 5% CO2.

Primary hepatocyte isolation

For primary mouse hepatocytes (PMH) isolation, anesthesia was induced using pentobarbital sodium at a dose of 50 mg/kg of body weight. The livers underwent complete digestion through portal vein perfusion employing Liver Perfusion Medium (17701-038, Life Technologies) followed by Liver Digest Medium (17703-034, Life Technologies), administered at a rate of 2

ml/min for 5 min for each solution. Subsequent to digestion, the liver tissue was excised, finely minced, and sieved through a 70 μm cell strainer (352350, Falcon). Primary hepatocytes were subsequently isolated through centrifugation at 50 × g for 3 min for 3 times. Primary human hepatocytes (PHH) were isolated similarly after portal vein branch perfusion.

Cell hypoxia reoxygenation (H/R) model

The cells were cultured in DMEM containing 10% fetal bovine serum (FBS) and 4500 mg/L glucose in a normal incubator for adherence. For hypoxia, the cells were rinsed with PBS and the medium was changed to glucose-free DMEM without FBS and placed to a hypoxia incubator of 5% CO_2, 94% N_2 and 1% O_2. After 10 h, the cells were rinsed with PBS and the medium was changed to DMEM with 10% FBS and 4500 mg/L glucose and placed in a normal incubator for reoxygenation. After indicated time, the cells were analyzed.

Reagent treatment

z-VAD-fmk (HY-16658B), Nec-1s (HY-14622A), GSK'872 (HY-101872), and disulfiram (HY-B0240), NAC (HY-B0215), SP600125 (HY-12041), α-tocopherol (HY-N0683) and palmitic acid (HY-N0830) were obtained from MedChemExpress. For cell experiments, the inhibitors were administered 1 h before H/R. For RIPK1 inhibition in rats, the livers were perfused and stored in cold UW solution supplemented with Nec-1s for a final concentration of 10 μM. For RIPK1 inhibition in mice, Nec-1s was injected intraperitoneally at a dose of 10 mg/kg 1h before I/R operation. For ROS inhibition in mice, NAC was injected intraperitoneally at a dose of 600 mg/kg 1h before I/R operation. For lipid ROS inhibition in mice, α- tocopherol was injected intraperitoneally at a dose of 50 mg/kg 1h before I/R operation.

Construction and injection of AAV8 or lentivirus

The AAV8 delivery system was employed to knock down *Casp8*, *Tnfr1*, *Zbp1* in the livers of rats (pAAV-U6-shRNA-WPRE) and overexpress *Zbp1* or its truncation mutants in the livers of mice (pAAV-TBGp-MCS-Flag-SV40 PolyA). AAV8 vectors were generated through the transfection of three plasmids (pAAV flanked by the AAV inverted terminal repeat sequences, pAAV8 trans-plasmid with the AAV rep and cap genes, and the pAAV helper plasmid) into HEK293T cells. The AAV titers were quantified at 1×10^{12} viral genomes per milliliter (v.g./ml). Overexpression Subsequently, mice were administered 2×10^{11} v.g. in 200 μl of virus and rats were administered 6×10^{11} v.g. in 600 μl of virus via the tail vein. Target sequences were as follows: rat *Casp8* (GGTTTCTGCCTACAGGGTT), rat *Tnfr1* (CCGGGAGAAGAGGGATAAT), rat *Zbp1* (GCCTCATCTCTATTTCCAA). Lentivirus was constructed to knock down c-Jun or ZBP1 in PHH and the target sequence were as follows: human *JUN* (CGCAAACCTCAGCAACTTCAA), human *ZBP1* (GCACAATCCAATCAACATGAT). All the viruses used in this study were procured from GeneChem.

Immunoblot

Primary antibodies against the following proteins were used for western blot analysis: mouse or rat p-RIPK1 (S166) (Cell Signaling Technology, 53286, 1:1000), human p-RIPK1 (S166) (Cell Signaling Technology, 65746, 1:1000), RIPK1 (Cell Signaling Technology, 3493, 1:1000), mouse or rat GSDMD (Abcam, ab219800, 1:1000, UK), human GSDMD (Abcam, ab215203, 1:1000), cleaved Caspase-3 (Cell Signaling Technology, 9661, 1:1000), mouse or rat cleaved caspase-8 (Cell Signaling Technology, 8592, 1:1000), human cleaved caspase-8 (Cell Signaling Technology,

9496, 1:1000), mouse or rat RIPK3 (Cell Signaling Technology, 15828, 1:1000), human RIPK3 (Abcam, ab305054, 1:1000), mouse or rat p-RIPK3 (T231/S232) (Cell Signaling Technology, 91702, 1:1000), human p-RIPK3 (S227) (Abcam, ab209384, 1:1000), MLKL (Abcam, ab243142, 1:1000), mouse or rat p-MLKL (S345) (Cell Signaling Technology, 37333, 1:1000), human p-MLKL (S358) (Cell Signaling Technology, 91689, 1:1000), Histone H3 (Cell Signaling Technology, 9715, 1:1000), Caspase-3 (Proteintech, 19677-1-AP, 1:1000), Caspase-8 (Proteintech, 13423-1-AP, 1:1000), ZBP1 (AdipoGen, AG-20B-0010-C100, 1:1000), JNK (Cell Signaling Technology, 9252, 1:1000), p-JNK (T183/Y185) (Cell Signaling Technology, 4668, 1:1000), c-Jun (Cell Signaling Technology, 9165, 1:1000), p-c-Jun (S73) (Cell Signaling Technology, 3270, 1:1000), p-c-Jun (S63) (Cell Signaling Technology, 91952, 1:1000), β-Tubulin (TransGen, HC101-01, 1:5000, China), GPX4 (Proteintech, 67763-1-Ig, 1:1000), FSP1 (Proteintech, 20886-1-AP, 1:1000), ACSL4 (Proteintech, 22401-1-AP, 1:1000). Second antibodies used in this study included: HRP-conjugated Affinipure Goat Anti-Rabbit IgG(H+L) (Proteintech, SA00001-2, 1:5000), HRP-conjugated Affinipure Goat Anti-Mouse IgG(H+L) (Proteintech, SA00001-1, 1:5000), HRP-conjugated Affinipure Goat Anti-Rat IgG(H+L) (Proteintech, SA00001-15, 1:5000). The signals were detected by Immobilon ECL Ultra Western HRP Substrate (Millipore). The membranes were reprobed after incubation in Restore Western Blot stripping buffer (Thermo). immunoblot under non-reducing conditions were performed using loading buffer without reducing agents.

Analysis of cytotoxicity and viability

For the assessment of cell death rates, primary hepatocytes were treated as described. After reoxygenation, SytoxGreen (Invitrogen) was added to the medium to the final concentration of 5

mM. The fluorescence intensity of SytoxGreen was detected through fluorescence enzyme marker detection (BioTek). Percentage of cell death was calculated as (indicated fluorescence - background fluorescence) / (max fluorescence - background fluorescence). The maximal fluorescence is induced by full permeabilization of the cells using Triton X-100 at a final concentration of 0.1%.

Immunoprecipitation

The cells underwent two washes using ice-cold PBS. Following this, the cells were lysed in 1 ml of 0.5% NP-40 lysis buffer containing 50 mM Tris-HCl pH 7.5, 150 mM NaCl, 0.5% NP-40, and 5% glycerol, supplemented with phosphatase and protease inhibitors along with. The lysates were gently overhead rotated at 4 °C for 30 min and subsequently clarified by centrifugation at 12,000 × g for 15 min at 4 °C. Immunoprecipitation of proteins was carried out from the cleared lysates using indicated antibody with overnight overhead rotation at 4 °C. The next day, protein A/G magnetic beads were added (MedChemExpress, HY-K0202) and underwent overhead rotation at 4 °C for 2 h, which was followed by three washes in 0.5% NP-40 buffer, and the elution of samples was performed by boiling in 50 μl of 1× SDS loading buffer for subsequent analysis via immunoblot.

RNA isolation and quantitative reverse-transcription PCR (qRT-PCR)

Total RNA was extracted from liver tissue or primary hepatocytes using the RNeasy Plus Mini kit (Qiagen, 74134). Subsequently, cDNA synthesis was carried out utilizing the SYBR qPCR Master Mix (Vazyme, Q311-02). The quantitative real-time polymerase chain reaction (qRT-PCR) was conducted employing SYBR green (Biorad, 1725275)-based qPCR on a Biorad CFX96 machine.

Primers used were as follows: mouse *Tnf* (forward: CCCTCACACTCAGATCATCTTCT, reverse: GCTACGACGTGGGCTACAG), human *TNF* (forward: GAGGCCAAGCCCTGGTATG, reverse: CGGGCCGATTGATCTCAGC), mouse *Il6* (forward: CCAAGAGGTGAGTGCTTCCC, reverse: CTGTTGTTCAGACTCTCTCCCT), human *IL6* (forward: CCTGAACCTTCCAAAGATGGC, reverse: TTCACCAGGCAAGTCTCCTCA), mouse *Il1b* (forward: GCAACTGTTCCTGAACTCAACT, reverse: ATCTTTTGGGGTCCGTCAACT), human *IL1B* (forward: ATGATGGCTTATTACAGTGGCAA, reverse: GTCGGAGATTCGTAGCTGGA), mouse *Ccl2* (forward: TTAAAAACCTGGATCGGAACCAA, reverse: GCATTAGCTTCAGATTTACGGGT), human *CCL2* (forward: CAGCCAGATGCAATCAATGCC, reverse: TGGAATCCTGAACCCACTTCT), mouse *Zbp1* (forward: AAGAGTCCCCTGCGATTATTTG, reverse: TCTGGATGGCGTTTGAATTGG), human *ZBP1* (forward: AACATGCAGCTACAATTCCAGA, reverse: AGTCTCGGTTCACATCTTTTGC).

ChIP-qPCR and luciferase assay

For ChIP-qPCR, SimpleChIP® Plus Sonication Chromatin IP Kit (Cell Signaling Technology, 56383) and c-Jun antibody (Cell Signaling Technology, 9165) were used according to the manufacturer's instructions. Primers used to expand *ZBP1* promoter were as follows: forward: ATCAGAGTGATGCCACGCC, reverse: TTCTGAATCAGCATCAGAGAACT. For luciferase assay, dual luciferase reporter gene detection kit (Beyotime, RG027) was applied according to the manufacturer's instructions. The c-Jun binding sites in *ZBP1* promoter were as follows: TAAGGGTGATTCAC, AGAGTGATGCCAC.

Transcriptome profiling

Tissues were homogenized in a 1.5 ml tube containing 1 ml of Trizol Reagent (Invitrogen). Total RNA extraction was performed according to the manufacturer's instructions. RNA was resuspended in DEPC-treated RNase-free water and residual DNA contamination was removed by TURBO DNA-free kit according to manufacturer's instruction (Invitrogen). The quantity and quality of RNA were determined by Nanodrop (Thermo scientific) and agarose gel electrophoresis. 1 μg of total RNA was used for sequencing library preparation. PolyA-tailed RNAs were selected by VAHTS mRNA-seq V2 Library Prep Kit for Illumina (Vazyme) according to manufacturer's instruction. The library quality was examined by Bioanalyzer 2100 (Agilent). Quantification was performed by qRT-PCR with a standard library as reference. The libraries were pooled together in equimolar amounts to a final 2 nM concentration. The normalized libraries were denatured with 0.1 M NaOH. Pooled libraries were sequenced on the Illumina X-ten platforms with PE150 (Illumina). Data was collected by Illumina X-ten platforms software version HCS 3.3.76 (Illumina). The transcriptome profiling analysis was assisted by Metware.

Ultra Performance Liquid Chromatography-Tandem mass spectrometry (UPLC-MS/MS)

For the quantification of palmitic acid in ND- or CDHFD-fed mouse livers, UPLC (ExionLC AD)-MS/MS (QTRAP) was utilized. Chromatographic columns was Waters ACQUITY UPLC HSS T3 C18 1.8 μm, 2.1 mm*100 mm. Mobile phase: phase A was ultra-pure water (0.1% formic acid), phase B was acetonitrile (0.1% formic acid). Elution gradient: 0 min water/acetonitrile (95: 5 V/V), 11.0 min 10: 9 V/V, 12.0 min 10: 9 V/V, 12.1 min 95: 5 V/V, 14.0 min 95: 5 V/V. Velocity of flow was 0.4 ml/min, Column temperature was 40 °C, sample size was 2μl. For Mass spectrum

acquisition conditions, electrospray ionization temperature was 500 °C, mass spectrum voltage was 5500V (positive), -4500 V (negative), GS I was 55 psi, GS II was 60 psi, curtain gas was 25 psi, collision-activated dissociation was set to high. Based on self-built targeted standard database, qualitative analysis was conducted according to the retention time of the detected substance, information of parent and daughter ion pairs and secondary spectrum data, quantification analysis was conducted utilizing multiple reaction monitoring of triple quadrupole mass spectrometry. The UPLC-MS/MS analysis was assisted by Metware.

TUNEL staining

The liver tissues collected were fixed in 4% paraformaldehyde and subsequently underwent processing for paraffin embedding. The detection of dead cells with DNA fragmentation was performed through the TUNEL assay, utilizing the In Situ Cell Death Detection Kit, POD (Roche), following the manufacturer's protocol. TUNEL positivity was analyzed with Fiji software.

Biochemical serum analysis

The blood serum levels of alanine aminotransferase (ALT) and aspartate transaminase (AST) were determined utilizing the Cobas C111 biochemical analyzer (Roche), following the manufacturer's instructions.

Immunofluorescence and immunohistochemistry

Liver tissues were fixed with 4% paraformaldehyde and underwent paraffin embedding. Subsequently, sections were dewaxed, and antigen retrieval was performed with 0.01 M sodium citrate. The sections were initially blocked with 3% H_2O_2, followed by 5% goat serum, and then incubated with primary antibodies at 4 °C overnight. Afterward, they were washed in phosphate-

buffered saline with Tween (PBST) before incubating with secondary antibodies at room temperature (RT) for 2 h. Images were captured using microscopy with a 20× or 40× objective.

Oil red O staining

Oil Red O staining was conducted with minor adjustments to assess neutral lipids and lipid droplet morphology. Tissues were collected from mice or rats of diverse genotypes and fixed in 4% paraformaldehyde. Subsequently, they were rinsed in PBS, and after an overnight incubation at 4 °C in 30% sucrose, tissues were frozen in a 2:1 mixture of 30% sucrose and Tissue-Tek optimal cutting temperature compound (OCT). Frozen liver sections, prepared using OCT compound, were immersed in a freshly prepared 1% Oil Red O working solution for 10 min, followed by counterstaining with hematoxylin. Afterward, they were rinsed under running tap water for 30 min. Photomicrographs were captured using a microscope. Lipid content was analyzed with Fiji software.

Enzyme Linked Immunosorbent Assay (ELISA)

Serum TNFα, IL6, CCL2 concentrations were determined with the following ELISA kit according to manufacturer's instructions: mouse TNFα (Proteintech, KE10002), rat TNFα (Proteintech, KE20018), mouse IL6 (Proteintech, KE10091), rat IL6 (Abclonal, RK00020, China), mouse CCL2 (Proteintech, KE10006), rat CCL2 (Proteintech, KE20009). Human serum TNFα, IL6, CCL2 levels were copied from the recipients' medical records.

Supplemental Figures and Supplemental Figure legends

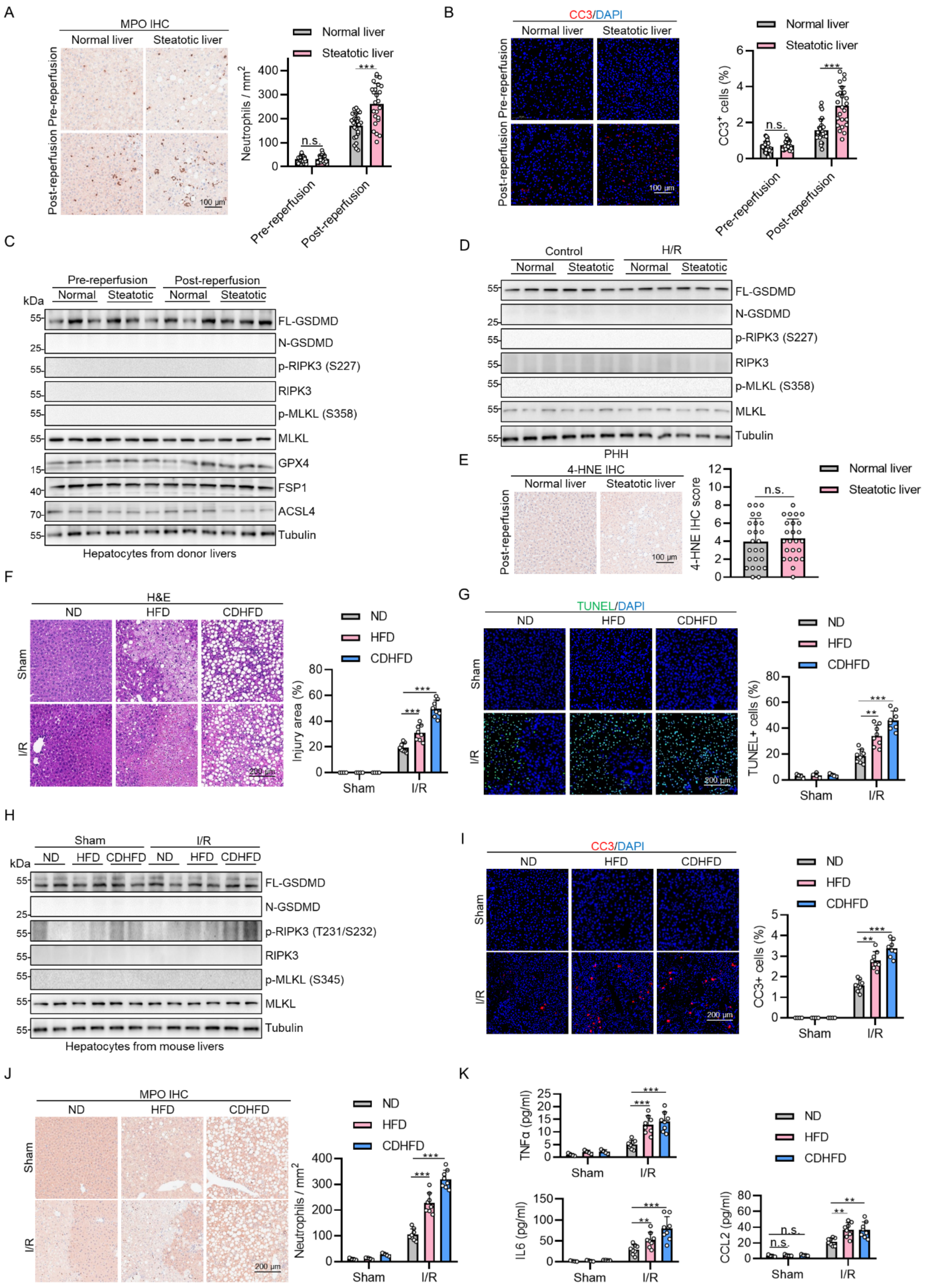

A
MPO IHC
Normal liver
Steatotic liver
Pre-reperfusion
Post-reperfusion
100 µm
Neutrophils / mm2
n.s.
***
B
CC3/DAPI
CC3+ cells (%)
C
Pre-reperfusion
Post-reperfusion
Normal
Steatotic
kDa
FL-GSDMD
N-GSDMD
p-RIPK3 (S227)
RIPK3
p-MLKL (S358)
MLKL
GPX4
FSP1
ACSL4
Tubulin
Hepatocytes from donor livers
D
Control
H/R
PHH
E
4-HNE IHC
4-HNE IHC score
F
H&E
ND
HFD
CDHFD
Sham
I/R
200 µm
Injury area (%)
G
TUNEL/DAPI
TUNEL+ cells (%)
H
p-RIPK3 (T231/S232)
p-MLKL (S345)
Hepatocytes from mouse livers
I
CC3+ cells (%)
J
K
TNFα (pg/ml)
IL6 (pg/ml)
CCL2 (pg/ml)

Supplemental Figure 1. Apoptosis and inflammation are exacerbated in steatotic liver I/R injury. Related to Figure 1.

(**A**) Steatotic donor livers show more neutrophil infiltration than normal donor livers post-reperfusion. Left: pre-reperfusion and post-reperfusion donor liver samples were analyzed with immunohistochemistry (IHC) of myeloperoxidase (MPO). Right: MPO positive rate was compared between normal and steatotic donor livers. Mean ± SD. n = 25 for normal donor livers and n = 24 for steatotic donor livers. Two-way ANOVA, post hoc Bonferroni's test.

(**B**) Steatotic donor livers show more apoptosis than normal donor livers post-reperfusion. Left: pre-reperfusion and post-reperfusion donor liver samples were analyzed with immunostaining of cleaved-caspase-3 (CC3). Right: CC3 positive rate was compared between normal and steatotic donor livers. Mean ± SD. n = 25 for normal donor livers and n = 24 for steatotic donor livers. Two-way ANOVA, post hoc Bonferroni's test.

(**C**) Necroptosis, pyroptosis and ferroptosis are similar between normal donor livers and steatotic donor livers. Hepatocytes from normal donor livers and steatotic donor livers were analyzed with immunoblot for proteins associated with necroptosis, pyroptosis and ferroptosis.

(**D**) Necroptosis and pyroptosis does not contribute to H/R induced steatotic hepatocyte death. PHH was isolated from pre-reperfusion donor livers and subjected to H/R challenge. Necroptosis and pyroptosis markers were analyzed by immunoblot.

(**E**) Lipid peroxidation is similar between normal donor livers and steatotic donor livers. Left: post-reperfusion donor liver samples were analyzed with IHC of 4-Hydroxynonenal (4-HNE). Right: 4-HNE IHC scores were compared between normal donor livers and steatotic donor livers. Mean

± SD. *n* = 25 for normal donor livers and *n* = 24 for steatotic donor livers. Unpaired Student's t test.

(F-K) Steatosis aggravates liver injury in mouse I/R model. ND-, HFD- and CDHFD-fed mice were subjected to 1 h ischemia/6 h reperfusion operation. Injury areas were detected with H&E staining (F). Cell death was detected with TUNEL staining (G). Necroptosis and pyroptosis markers were analyzed by immunoblot (H). Apoptosis was detected with CC3 immunostaining (I). Neutrophil infiltration was analyzed with MPO IHC (J). Serum levels of proinflammatory cytokines TNFα, IL6, CCL2 were detected with ELISA (K). Mean ± SD. *n* = 4 for each sham group and *n* = 8 for each I/R group. Two-way ANOVA, post hoc Bonferroni's test.

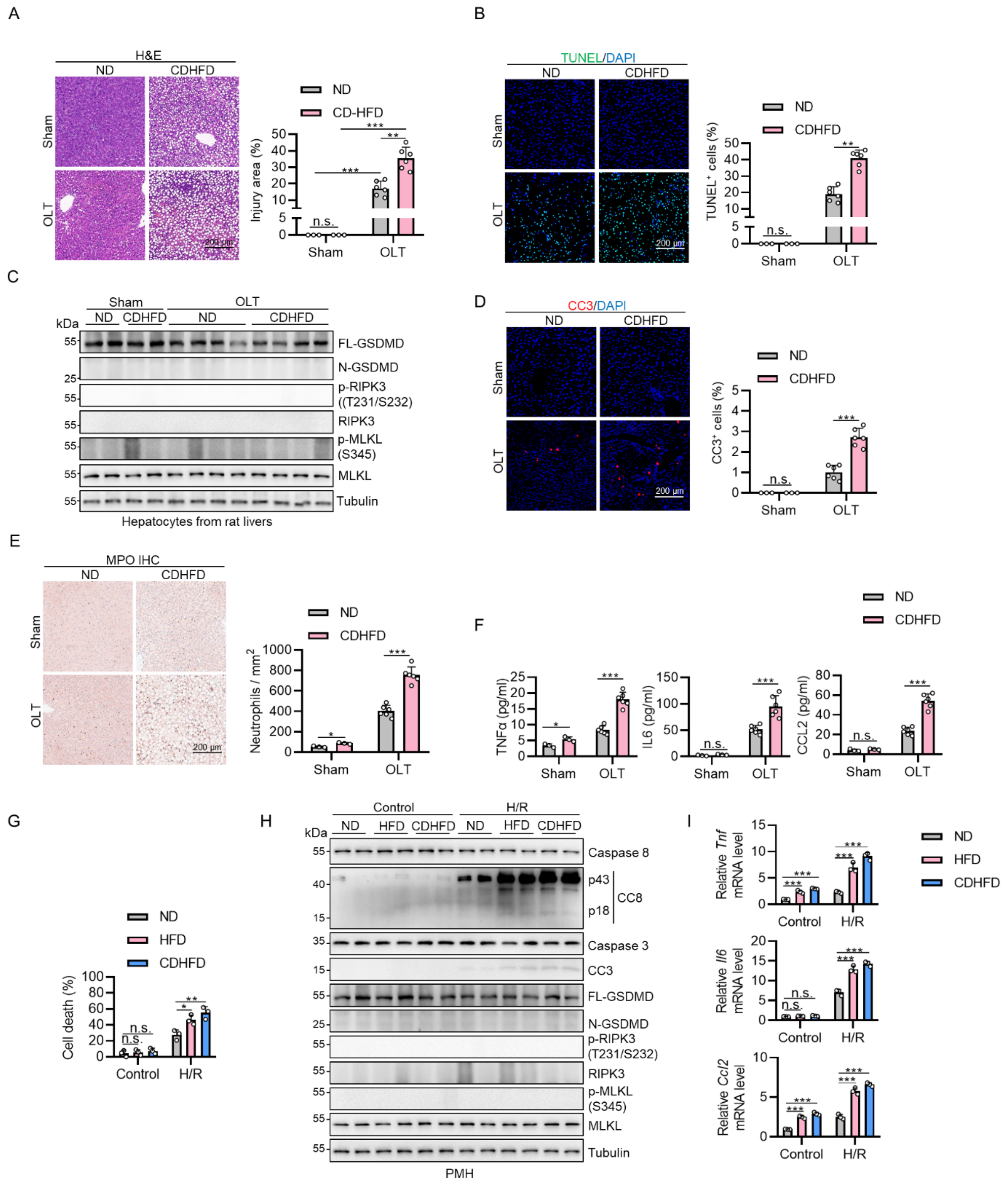

Supplemental Figure 2. Aggravation of hepatic apoptosis and inflammation in steatotic liver I/R. Related to Figure 1.

(**A-F**) Steatosis aggravates liver injury in rat OLT model. Donor livers from ND- or CDHFD-fed

rats were implanted to healthy rats after 18 h cold storage or experienced sham operation. Injury areas were detected with H&E staining (A). Cell death was detected with TUNEL staining (B). Necroptosis and pyroptosis markers were analyzed by immunoblot (C). Apoptosis was detected with CC3 immunostaining (D). Neutrophil infiltration was analyzed with MPO IHC (E). Serum levels of proinflammatory cytokines TNFα, IL6, CCL2 were detected with ELISA (F). Mean ± SD. n = 3 for each sham group and n = 6 for each OLT group. Two-way ANOVA, post hoc Bonferroni's test.

(**G-I**) Steatosis aggravates cell death and inflammation in cell H/R model. PMH isolated from ND-, HFD-, CDHFD-fed mice were exposed to 10 h hypoxia. Cell death was measured with SytoxGreen positivity after 24 h reoxygenation (G). Cells were analyzed with immunoblot for cell death markers (H). mRNA levels of proinflammatory cytokines *Tnf*, *Il6*, *Ccl2* were detected with qRT-PCR after 3 h reoxygenation (I). Representative results were shown. Mean ± SD. n = 3 for each group. Two-way ANOVA, post hoc Bonferroni's test.

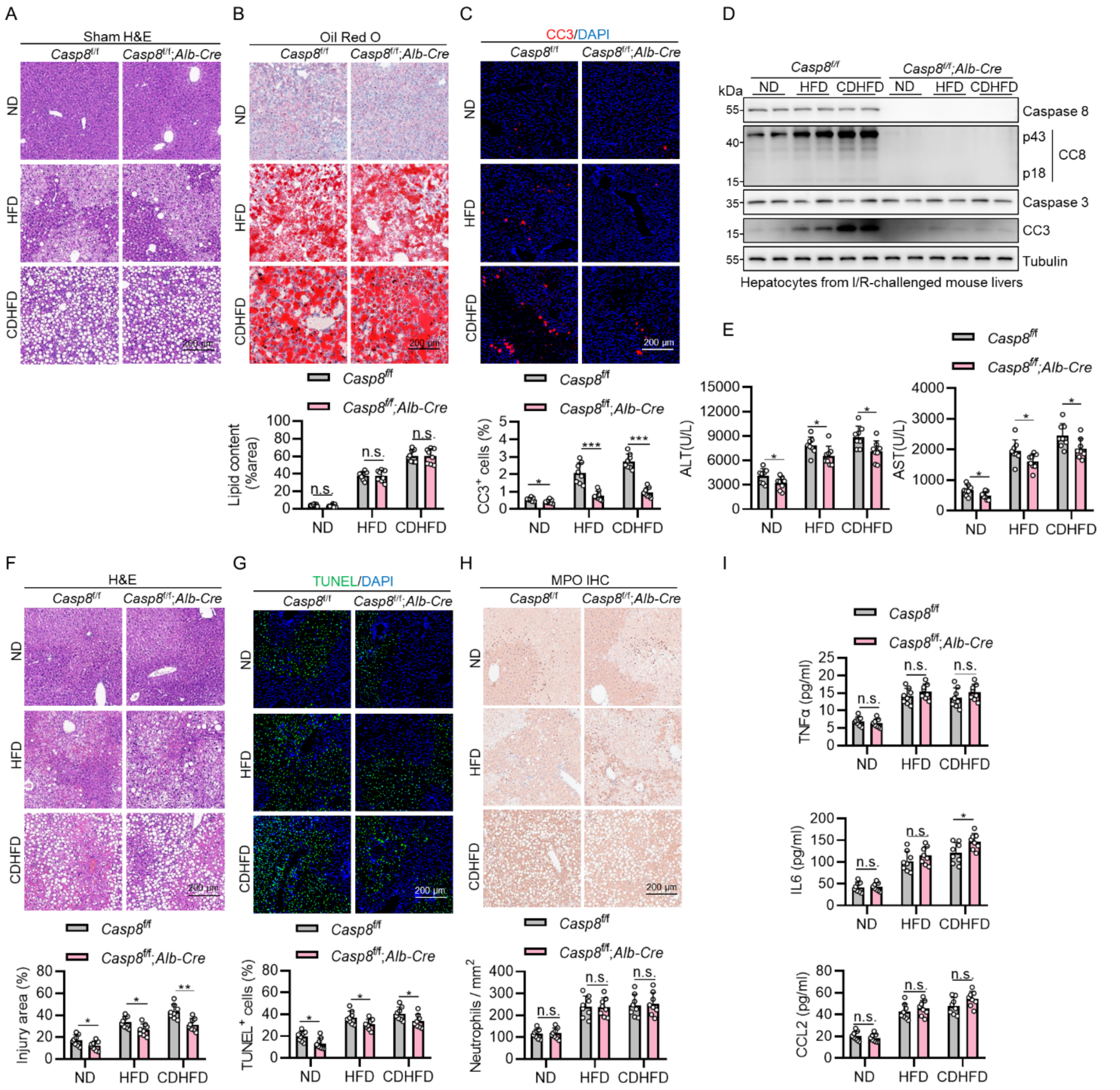

Supplemental Figure 3. Caspase-8-mediated hepatic apoptosis partially contributes to steatotic liver I/R injury.

(**A** and **B**) Hepatocyte *Casp8* knockout does not cause liver injury or regulate steatosis. Sham-operated livers were examined with H&E staining (A). Lipid distribution was analyzed with Oil Red O staining (B). Mean ± SD. $n = 8$ for each I/R group. Two-way ANOVA, post hoc Bonferroni's

test.

(**C** and **D**) Hepatocyte *Casp8* knockout significantly reduces apoptosis mouse liver I/R injury. ND-, HFD- and CDHFD-fed *Casp8*$^{f/f}$ or *Casp8*$^{f/f}$;*Alb-Cre* mice underwent 1 h ischemia/6 h reperfusion operation. Apoptosis was determined with CC3 immunostaining (C) and immunoblot (D). Mean ± SD. *n* = 8 for each I/R group. Two-way ANOVA, post hoc Bonferroni's test.

(**E**-**G**) Hepatocyte *Casp8* knockout slightly alleviates mouse liver I/R injury. ND-, HFD- and CDHFD-fed *Casp8*$^{f/f}$ or *Casp8*$^{f/f}$;*Alb-Cre* mice underwent 1 h ischemia/6 h reperfusion operation (n = 8). Serum ALT/AST detection (E), H&E staining (F), TUNEL staining (G) were performed. Mean ± SD. *n* = 8 for each I/R group. Two-way ANOVA, post hoc Bonferroni's test.

(**H** and **I**) Hepatocyte *Casp8* knockout does not alleviates inflammation in mouse liver I/R injury. ND-, HFD- and CDHFD-fed *Casp8*$^{f/f}$ or *Casp8*$^{f/f}$;*Alb-Cre* mice underwent 1 h ischemia/6 h reperfusion operation (n = 8). Neutrophil infiltration was analyzed with MPO IHC (H), serum cytokine concentrations were detected (I). Mean ± SD. *n* = 8 for each I/R group. Two-way ANOVA, post hoc Bonferroni's test.

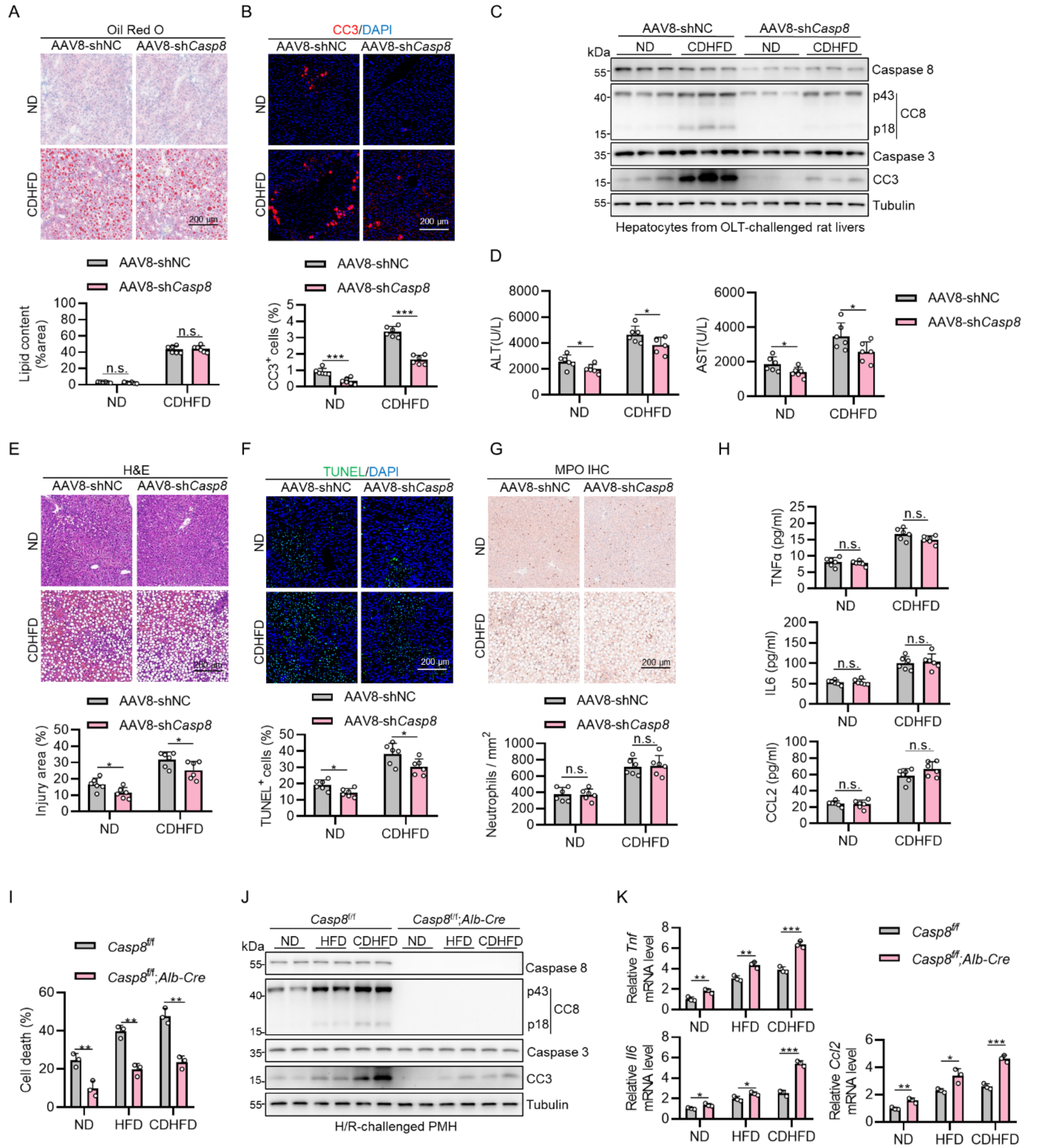

Supplemental Figure 4. Caspase-8-mediated hepatic apoptosis partially contributes to steatotic liver I/R injury in rat OLT and cell H/R model.

(**A-H**) Hepatocyte *Casp8* knockdown alleviates rat liver OLT injury. 6 × 10^{11} v.g. in 600 μl of

AAV8 were injected via the tail vein to knock down *Casp8* in hepatocytes of ND- and CDHFD-fed rats which were subjected to 18 h cold storage/6 h reperfusion operation 1 month after virus injection (n = 6). Oil Red O staining (A), CC3 staining (B), cell death analysis (C), serum ALT/AST detection (D), H&E staining (E), TUNEL staining (F), MPO IHC (G), detection of serum cytokine concentrations (H) were performed. All data are presented as the mean ± SD. Two-way ANOVA, post hoc Bonferroni's test.

(**I**-**K**) Knockout of *Casp8* reduces cell death but not inflammation. PMH isolated from ND-, HFD- and CDHFD-fed *Casp8*$^{f/f}$ or *Casp8*$^{f/f}$;*Alb-Cre* mice were exposed to 10 h hypoxia. Cell death was measured with SytoxGreen positivity after 24 h reoxygenation (I). Cells were analyzed for CC8 and CC3 with immunoblot after 3 h reoxygenation (J). Representative results were shown. mRNA levels of proinflammatory cytokines *Tnf*, *Il6*, *Ccl2* were detected with qRT-PCR after 3 h reoxygenation (K). Mean ± SD. n = 3 for each group. Two-way ANOVA, post hoc Bonferroni's test.

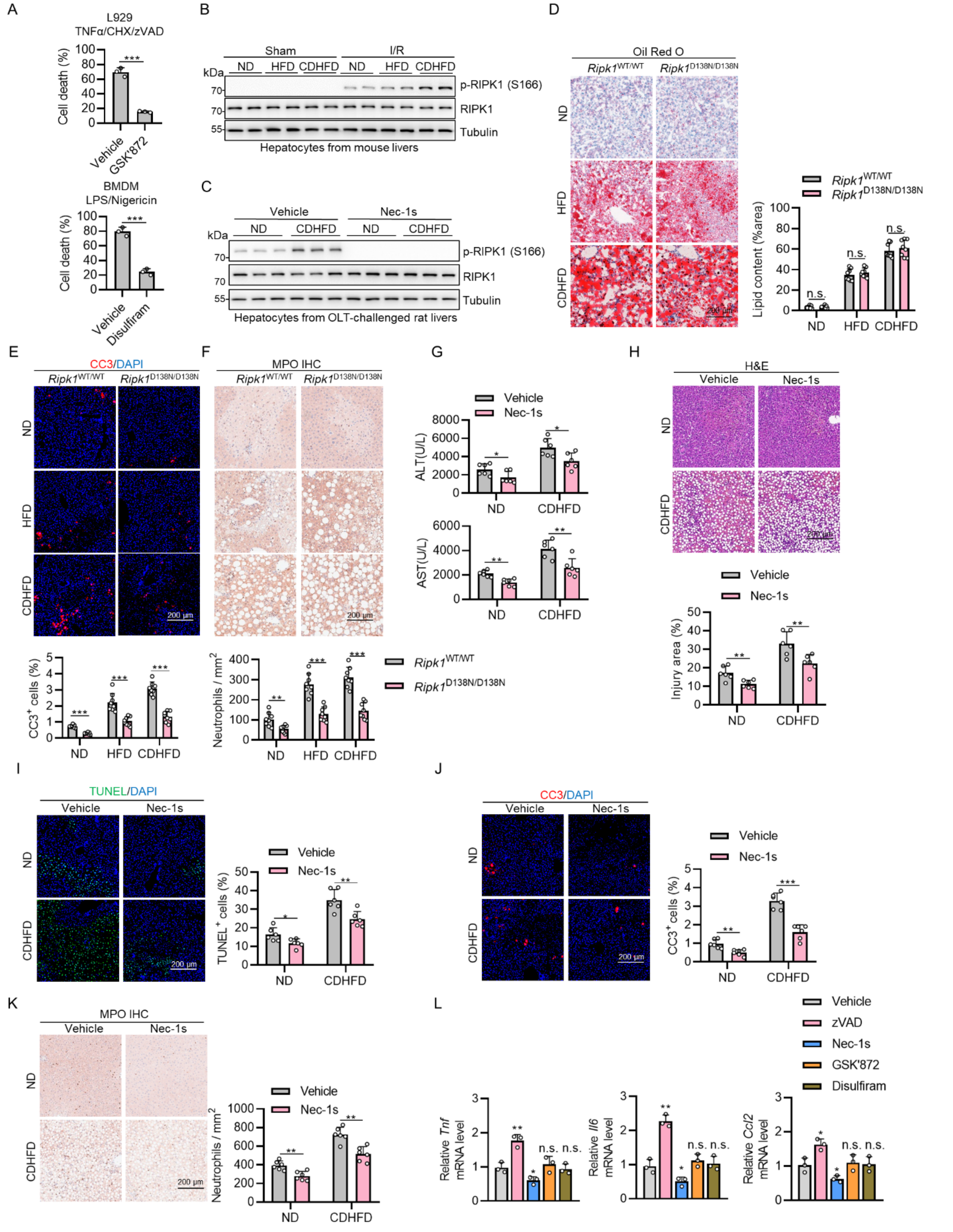

Supplemental Figure 5. Blocking RIPK1 kinase activity protects against liver I/R injury

through reducing apoptosis and inflammation. Related to Figure 2.

(**A**) The efficacy of GSK'872 and disulfiram are detected. Upper: L929 was pretreated with CHX (2 μg/ml), zVAD.fmk (10 μM) for 0.5 h followed by 1 ng/ml TNFα to induce necroptosis and cell death was determined after 24 h with SytoxGreen positivity. GSK'872 (1 μM) was pre-treated 0.5 h before TNFα to inhibit RIPK3. Lower: BMDM was primed with LPS (1 μg/ml) for 4 h and treated with nigericin (20 μM) to induce pyroptosis and cell death was determined after 24 h with SytoxGreen positivity. Disulfiram (50 μM) was pre-treated 0.5 h before nigericin to inhibit GSDMD oligomerization.

(**B**) Steatosis facilitates RIPK1 activation in liver I/R injury. Expression of activated RIPK1 in hepatocytes of ND-, HFD- or CDHFD-fed mice after I/R challenge was analyzed with immunoblot. Representative results out of n = 8 mice per group were shown.

(**C**) RIPK1 kinase activation is blocked by Nec-1s in rat OLT model. Livers of ND- or CDHFD-fed rat were perfused with and stored in cold UW solution supplemented with Nec-1s to a final concentration of 10 μM. After 18 h cold storage/6 h reperfusion, samples were obtained. Hepatocytes were analyzed with immunoblot for p-RIPK1 (S166). Representative results out of n = 6 rats per group were shown.

(**D**) RIPK1 kinase activity does not regulate liver steatosis. Oil Red O staining of ND-, HFD- and CDHFD-fed *Ripk1*$^{WT/WT}$ or *Ripk1*$^{D138N/D138N}$ mouse livers. Mean ± SD. n = 8 for each group. Two-way ANOVA, post hoc Bonferroni's test.

(**E** and **F**) Genetic inactivation of RIPK1 kinase protects against liver I/R injury. ND-, HFD-, CDHFD-fed *Ripk1*$^{WT/WT}$ or *Ripk1*$^{D138N/D138N}$ mice underwent 1 h ischemia/6 h reperfusion

operation. Apoptosis was determined with CC3 immunostaining (E). Neutrophil infiltration was analyzed with MPO IHC (F). Mean ± SD. *n* = 8 for each I/R group. Two-way ANOVA, post hoc Bonferroni's test.

(**G-K**) Blocking RIPK1 kinase activity with Nec-1s alleviates OLT-induced rat liver injury. Livers of ND- or CDHFD-fed rat were perfused with and stored in cold UW solution supplemented with Nec-1s to a final concentration of 10 μM. After 18 h cold storage/6 h reperfusion, samples were obtained. Serum ALT/AST detection (G), H&E staining (H), TUNEL staining (I), CC3 immunostaining (J), MPO IHC (K) were performed. Mean ± SD. *n* = 6 for each OLT group. Two-way ANOVA, post hoc Bonferroni's test.

(**L**) Blocking RIPK1 kinase inhibits inflammation. Hepatocytes were isolated from CDHFD-fed WT mice and exposed to 10 h hypoxia after 1 h pretreatment of zVAD (10 μM), Nec-1s (10 μM), GSK'872 (1 μM), and disulfiram (50 μM). mRNA levels of proinflammatory cytokines *Tnf*, *Il6*, *Ccl2* were detected with qRT-PCR after 3 h reoxygenation. Mean ± SD. *n* = 3 mice for each group. One-way ANOVA, post hoc Dunnett's test.

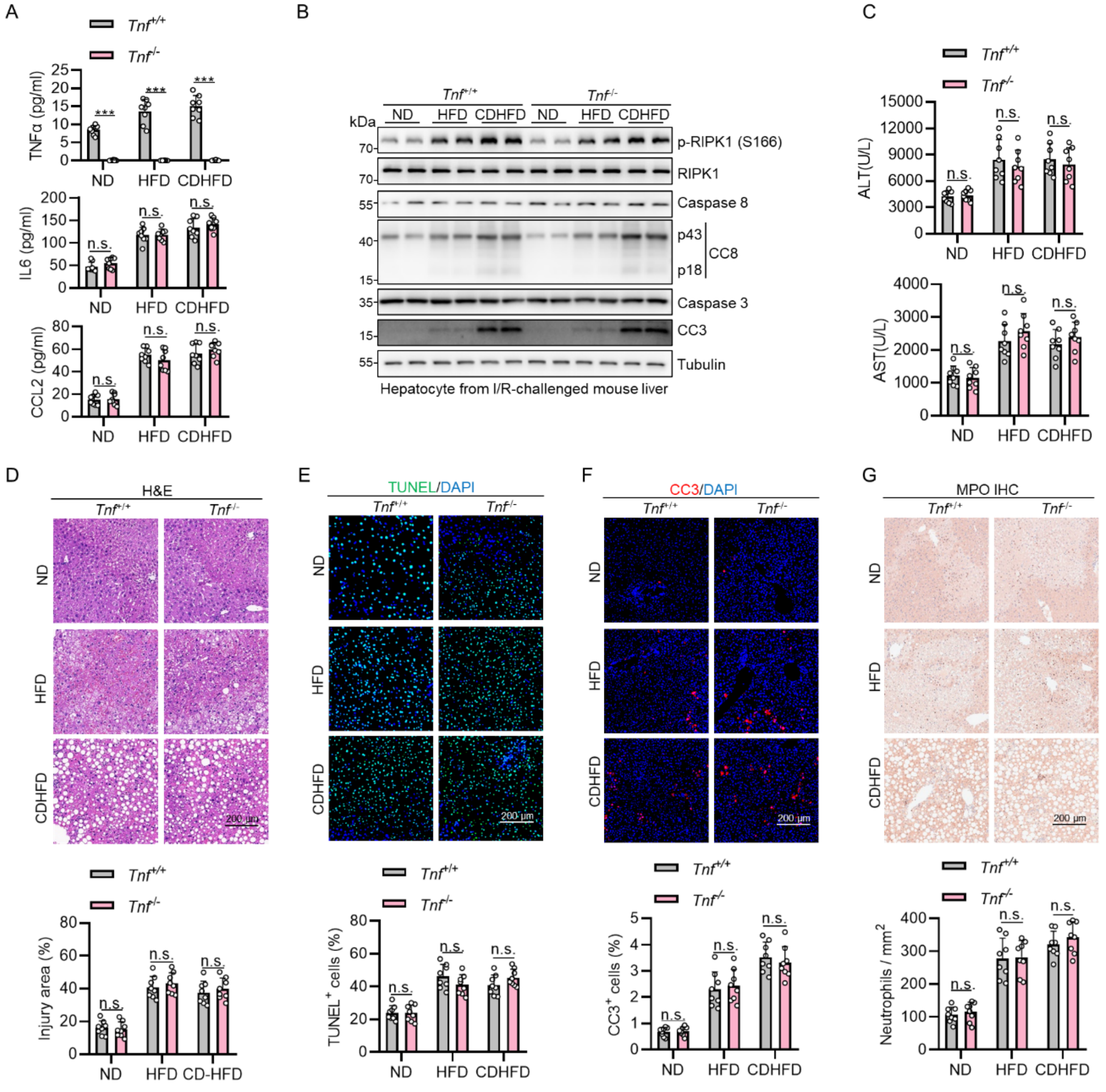

Supplemental Figure 6. Blocking TNFα pathway does not protect against liver I/R injury.

(**A**-**G**) Knockout of *Tnf* does not protect against liver I/R injury. ND-, HFD- and CDHFD-fed *Tnf*$^{+/+}$ or *Tnf*$^{-/-}$ mice underwent 1 h ischemia/6 h reperfusion operation. Serum levels of proinflammatory cytokines TNFα, IL6, CCL2 were detected with ELISA (A). p-RIPK1, CC8 and CC3 in hepatocytes were detected with immunoblot (B). Serum levels of ALT and AST were detected (C). Injury areas were detected with H&E staining (D). Cell death was detected with TUNEL staining (E). Apoptosis was detected with CC3 immunostaining (F). Neutrophil infiltration was analyzed

with MPO IHC (G). Mean ± SD. $n = 8$ for each I/R group. Two-way ANOVA, post hoc Bonferroni's test.

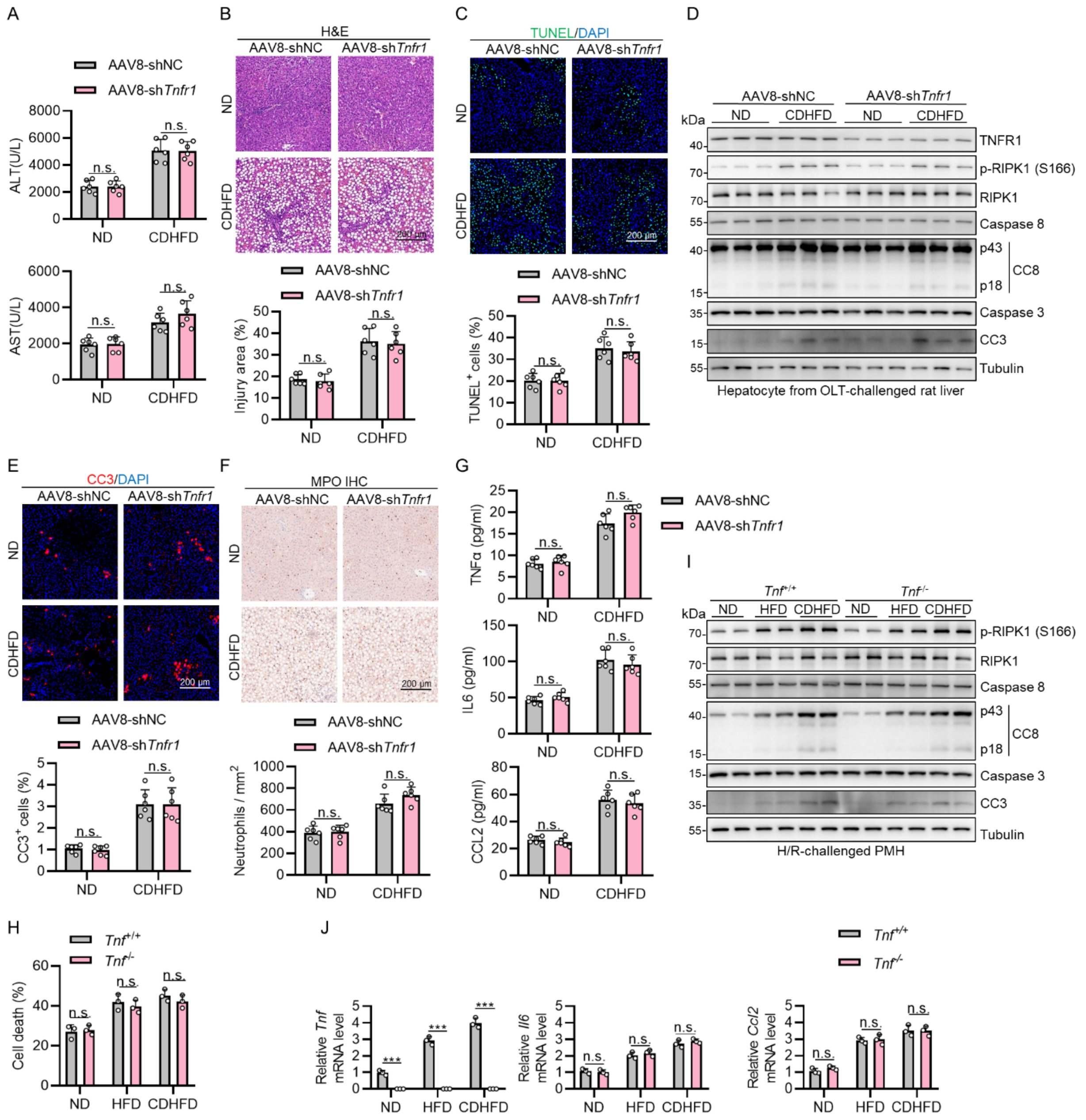

Supplemental Figure 7. Blocking TNF-α pathway does not provide protection against liver I/R injury in rat OLT and cell H/R model.

(**A-G**) Knockdown of *Tnfr1* does not protect against OLT-induced I/R injury. 6 × 10^{11} v.g. in 600 μl of AAV8 was injected to the rats via tail vein to knockdown *Tnfr1* in hepatocytes. After 1 month, ND- or CDHFD-fed rat livers underwent 18 h cold storage/6 h reperfusion. Serum levels of ALT

and AST were detected (A). Injury areas were detected with H&E staining (B). Cell death was detected with TUNEL staining (C). p-RIPK1, CC8 and CC3 in hepatocytes were detected with immunoblot (D). Apoptosis was detected with CC3 immunostaining (E). Neutrophil infiltration was analyzed with MPO IHC (F). Serum levels of proinflammatory cytokines TNFα, IL6, CCL2 were detected with ELISA (G). Mean ± SD. n = 6 for each group. Two-way ANOVA, post hoc Bonferroni's test.

(**H-J**) Knockout of *Tnf* does not regulate cell death or inflammation in cell H/R model. PMH isolated from ND-, HFD- and CDHFD-fed $Tnf^{+/+}$ or $Tnf^{-/-}$ mice were exposed to 10 h hypoxia. Cell death was measured with SytoxGreen positivity after 24 h reoxygenation (H). Cells were analyzed for p-RIPK1, CC8, CC3 with immunoblot after 3 h reoxygenation (I). Representative results were shown. mRNA levels of proinflammatory cytokines *Tnf*, *Il6*, *Ccl2* were detected with qRT-PCR after 3 h reoxygenation (J). Mean ± SD. n = 3 for each group. Two-way ANOVA, post hoc Bonferroni's test.

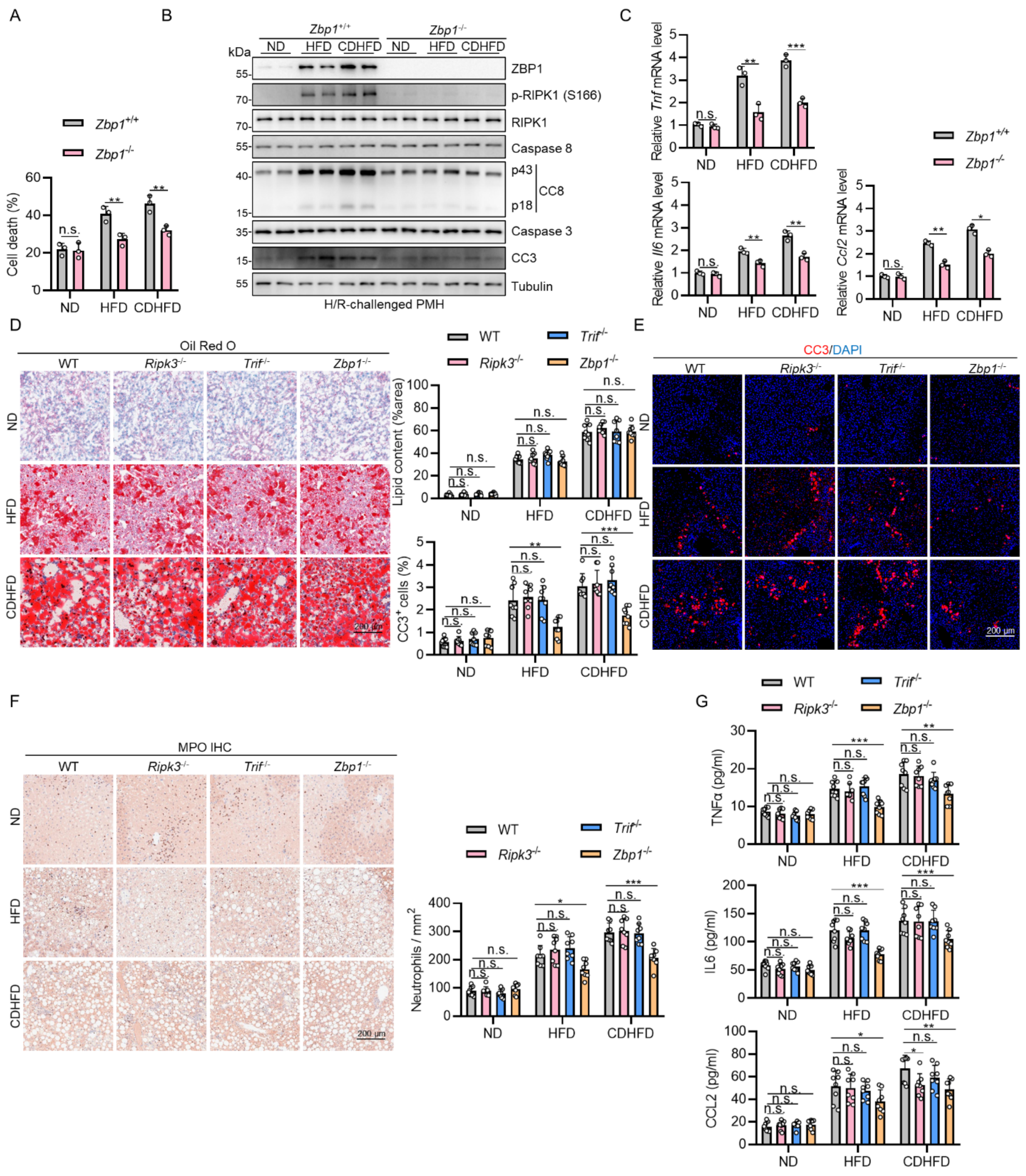

Supplemental Figure 8. RIPK1 is activated by ZBP1 in steatotic liver I/R injury. Related to Figure 3.

(**A-C**) Knockout of *Zbp1* decreases cell death and inflammation in steatotic cell H/R model. PMH isolated from ND-, HFD- and CDHFD-fed *Zbp1*$^{+/+}$ or *Zbp1*$^{-/-}$ mice were exposed to 10 h hypoxia.

Cell death was measured with SytoxGreen positivity after 24 h reoxygenation (A). Cells were analyzed for p-RIPK1, CC8, CC3 with immunoblot after 3 h reoxygenation (B). mRNA levels of proinflammatory cytokines *Tnf*, *Il6*, *Ccl2* were detected with qRT-PCR after 3 h reoxygenation (C). Representative results were shown. Mean ± SD. *n* = 3 for each group. Two-way ANOVA, post hoc Bonferroni's test.

(**D-G**) Knockout of ZBP1 protects against steatotic liver I/R injury. ND-, HFD-, CDHFD-fed WT, *Ripk3*$^{-/-}$, *Trif*$^{-/-}$, *Zbp1*$^{-/-}$ mice underwent 1 h ischemia/6 h reperfusion operation. Lipid distribution was measured with Oil Red O staining (D). Apoptosis was determined with CC3 immunostaining (E). Neutrophil infiltration was analyzed with MPO IHC (F). Serum levels of proinflammatory cytokines TNFα, IL6, CCL2 were detected with ELISA (G). Mean ± SD. *n* = 8 for each group. Two-way ANOVA, post hoc Bonferroni's test.

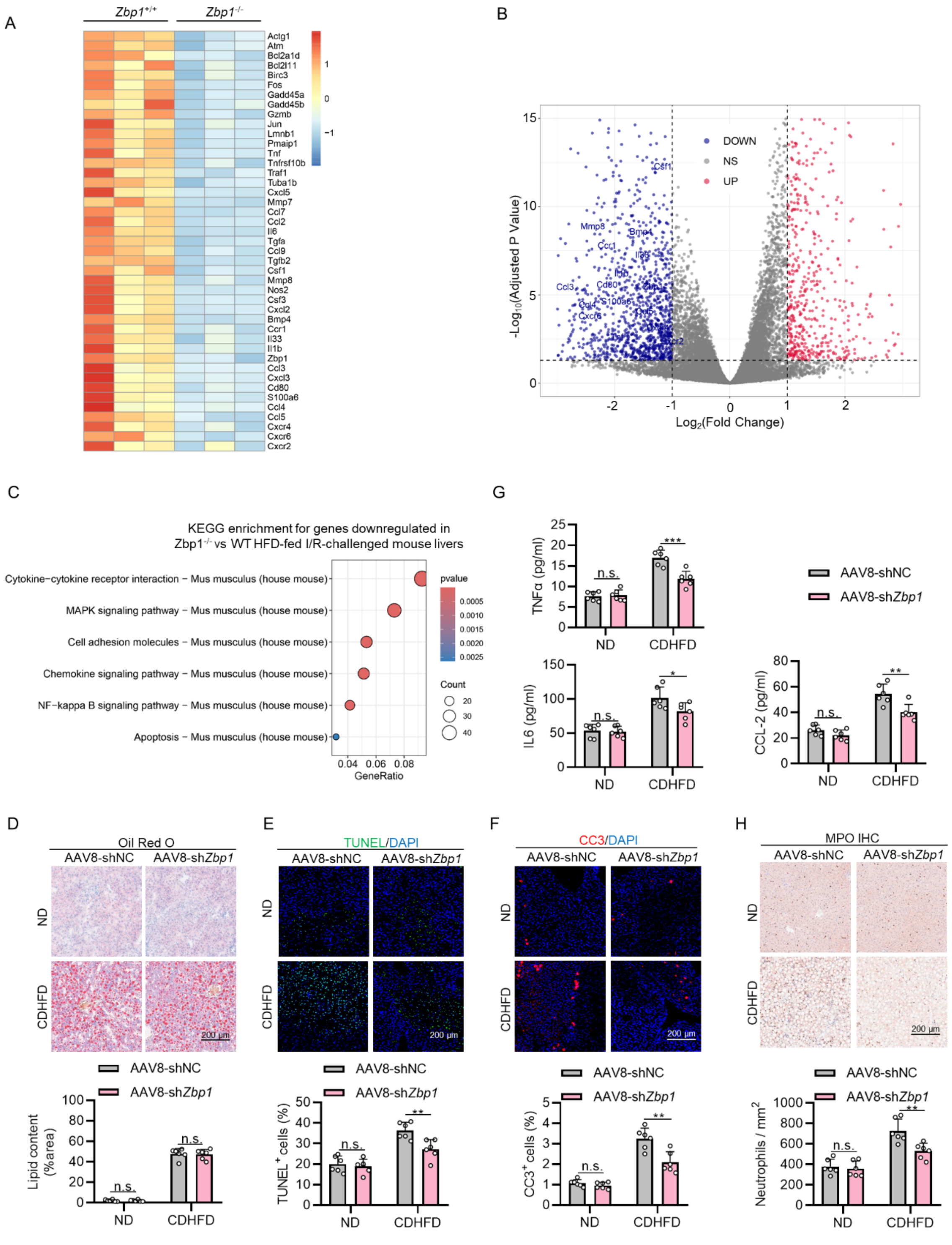

Supplemental Figure 9. Knockdown or knockout of ZBP1 protects against OLT or H/R-induced injury in steatotic hepatoctes. Related to Figure 3.

(**A-C**) Knockout of ZBP1 inhibited inflammatory response in steatotic liver I/R injury. HFD-fed

WT and *Zbp1*$^{-/-}$ mice underwent 1 h ischemia/6 h reperfusion operation. Transcriptome profiling of the livers were conducted. Differentially expressed genes were filtered with the criteria adjusted P value < 0.05 and $|\log_2 \text{foldchange}| > 1$. Differentially expressed genes of proinflammatory cytokines were shown with heatmap (A) and volcano plot (B). The genes decreased in *Zbp1*$^{-/-}$ livers were enriched for KEGG (C). *n* = 3 mice per genotype.

(**D**-**H**) Knockdown of *Zbp1* mitigates steatotic liver I/R injury in rat OLT model. 6×10^{11} v.g. in 600 μl of AAV8 was injected to the rats via the tail vein. After 1 month, ND- or CDHFD-fed rat livers underwent 18 h cold storage/6 h reperfusion. Lipid distribution was detected with Oil Red O staining (D). Cell death was measured with TUNEL staining (E). Apoptosis was determined with CC3 immunostaining (F). Serum levels of proinflammatory cytokines TNFα, IL6, CCL2 were detected with ELISA (G). Neutrophil infiltration was analyzed with MPO IHC (H). Mean ± SD. *n* = 6 rats for each group. Two-way ANOVA, post hoc Bonferroni's test.

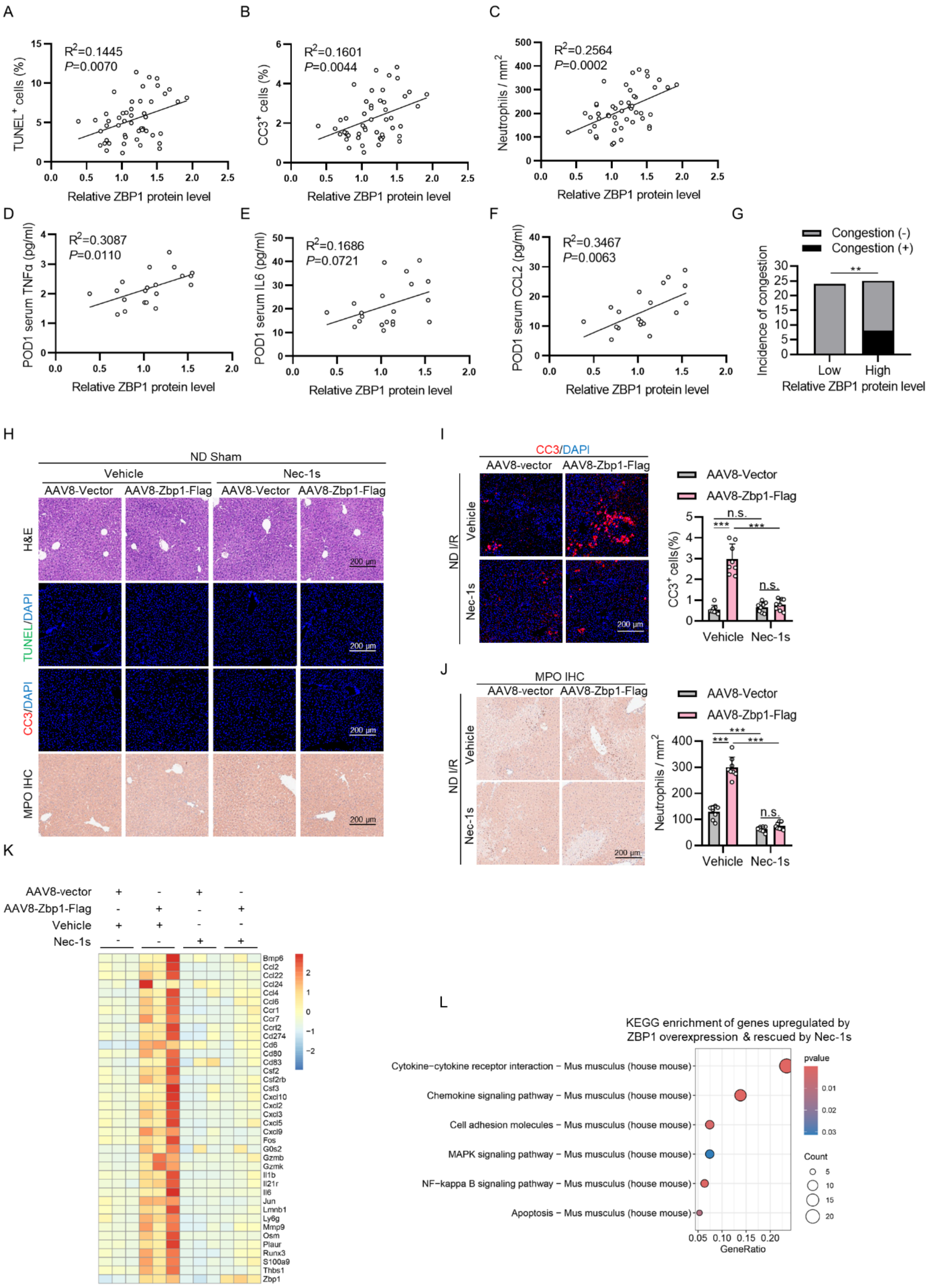

A
B
C
D
E
F
G
H
I
J
K
L
Relative ZBP1 protein level
TUNEL⁺ cells (%)
CC3⁺ cells (%)
Neutrophils / mm2
POD1 serum TNFα (pg/ml)
POD1 serum IL6 (pg/ml)
POD1 serum CCL2 (pg/ml)
Incidence of congestion
Congestion (-)
Congestion (+)
Low
High
ND Sham
Vehicle
Nec-1s
AAV8-Vector
AAV8-Zbp1-Flag
H&E
TUNEL/DAPI
CC3/DAPI
MPO IHC
200 μm
ND I/R
n.s.
Vehicle
Nec-1s
AAV8-vector
AAV8-Zbp1-Flag
KEGG enrichment of genes upregulated by ZBP1 overexpression & rescued by Nec-1s
Cytokine−cytokine receptor interaction − Mus musculus (house mouse)
Chemokine signaling pathway − Mus musculus (house mouse)
Cell adhesion molecules − Mus musculus (house mouse)
MAPK signaling pathway − Mus musculus (house mouse)
NF−kappa B signaling pathway − Mus musculus (house mouse)
Apoptosis − Mus musculus (house mouse)
GeneRatio
pvalue
Count

Supplemental Figure 10. ZBP1 is increased in steatotic liver, underlying its specificity in promoting steatotic liver I/R injury. Related to Figure 4.

(**A-G**) ZBP1 protein level positively correlates with the severity of liver injury in transplantation. Correlations between ZBP1 protein level and clinicopathological parameters regarding cell death or inflammation including TNUEL positivity (A), CC3 positivity (B), neutrophil infiltration (C) and serum levels of TNFα (D), IL6 (E), CCL2 (F) on POD1 and congestion incidence (G) were analyzed. n = 49 for TNUEL positivity, CC3 positivity, neutrophil infiltration, congestion incidence. n = 20 for serum levels of TNFα, IL6, CCL2. Fisher's exact test (G). Pearson's correlation test (A-F).

(**H**) Overexpression of ZBP1 does not trigger spontaneous cell death. 2×10^{11} v.g. in 200 μl of AAV8 were injected to the ND-fed WT mice via the tail vein to overexpress ZBP1. After 1 month, 10 mg/kg Nec-1s was injected intraperitoneally. After 1 h, the mice were subjected to sham operation. Liver injury was analyzed with H&E staining, TUNEL staining, CC3 immunostaining, MPO IHC. Representative images out of n = 4 mice for each group were shown.

(**I** and **J**) Overexpression of ZBP1 aggravates RIPK1-dependent liver injury in mouse I/R model. 2×10^{11} v.g. in 200 μl of AAV8 was injected to the ND-fed WT mice via the tail vein to overexpress ZBP1. After 1 month, 10 mg/kg Nec-1s was injected intraperitoneally. After 1 h, the mice were subjected to 1 h ischemia/6 h reperfusion challenge. Apoptosis was determined with CC3 immunostaining (I). Neutrophil infiltration was analyzed with MPO IHC (J). Mean ± SD. n = 8 mice for each group. Two-way ANOVA, post hoc Bonferroni's test.

(**K** and **L**) ZBP1 induces RIPK1 kinase-dependent inflammatory response. 2×10^{11} v.g. in 200 μl

of AAV8 were injected to the ND-fed WT mice via the tail vein to overexpress ZBP1. After 1 month, 10 mg/kg Nec-1s was injected intraperitoneally. After 1 h, the mice were subjected to 1 h ischemia/6 h reperfusion challenge. Transcriptome profiling of the mouse livers were conducted. Differentially expressed genes were filtered with the criteria adjusted P value < 0.05 and $|\log_2$ foldchange$| > 1$. The expression of genes that were upregulated by ZBP1 overexpression and downregulated by Nec-1s pretreatment were shown with heatmap (K) and were analyzed for KEGG enrichment (L). *n* = 3 mice for each group.

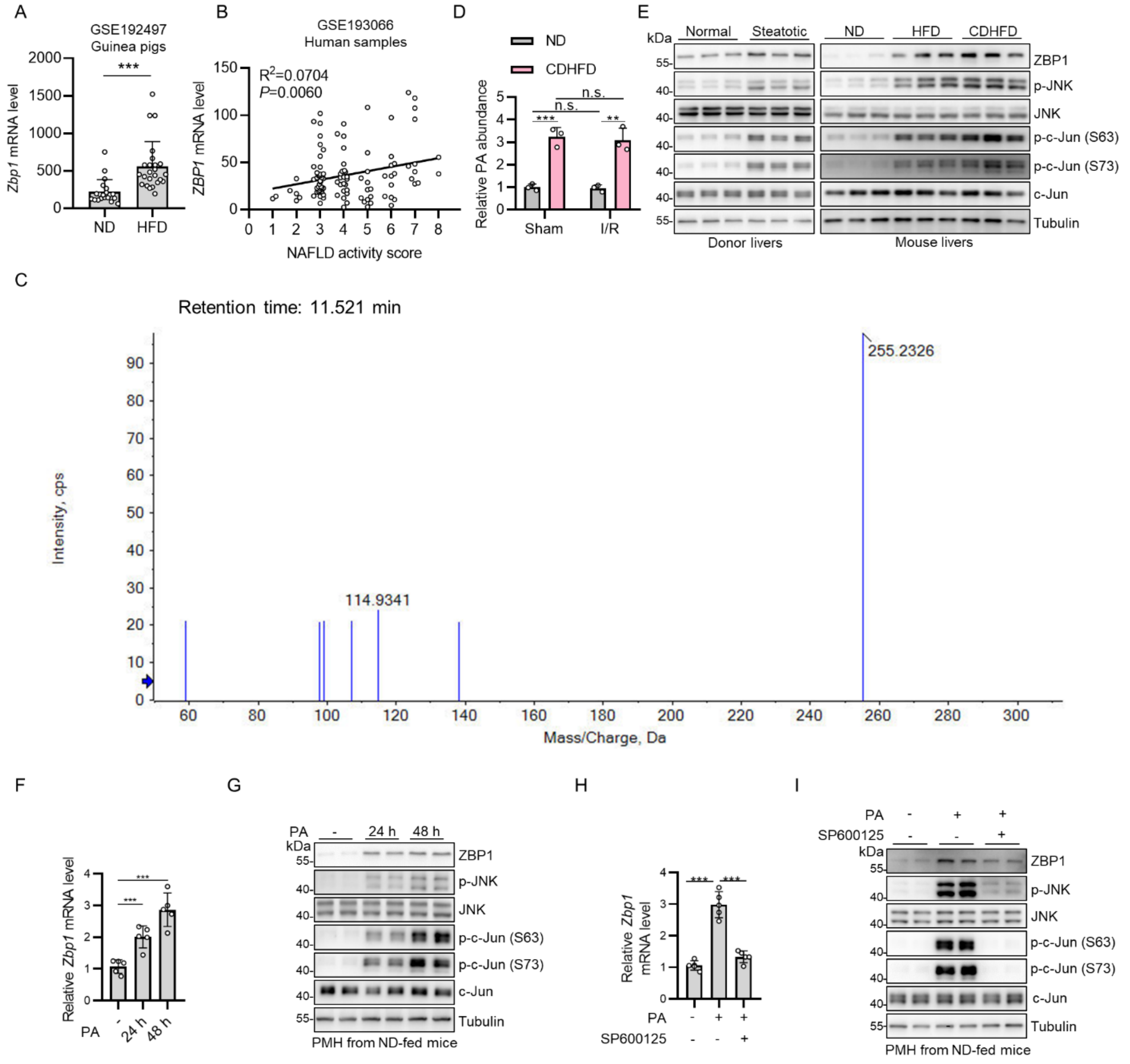

Supplemental Figure 11. Palmitic acid upregulates hepatocyte ZBP1 expression level through JNK pathway to exacerbate I/R injury of steatotic livers. Related to Figure 5.

(**A**) *ZBP1* mRNA expression level upregulation in steatotic livers is validated in GSE192497. The guinea pigs were fed HFD for 32 weeks and transcriptome profiling was conducted. *Zbp1* mRNA levels were compared between ND and HFD groups. Mean ± SD. n = 20 for ND group and n = 24 for HFD group. Unpaired two-tailed Student's t-test.

(**B**) *ZBP1* mRNA level positively correlates with the severity of steatosis in GSE193066. Liver samples of 106 patients of different non-alcoholic fatty liver disease (NAFLD) activity scores were harvested and conducted transcriptome profiling. The correlation between *ZBP1* mRNA level and NAFLD score was analyzed. Mean ± SD. Pearson's correlation test.

(**C** and **D**) Palmitic acid abundance is significantly increased in steatotic livers. Retention time and spectrum data of palmitic acid in UPLC-MS/MS analysis (C). Relative palmitic acid abundance in the livers of ND- or CDHFD-fed mice was compared (D). Mean ± SD. $n = 3$ for each group. Two-way ANOVA, post hoc Bonferroni's test.

(**E**) JNK pathway is activated in steatotic livers. p-JNK, p-c-Jun (S63) and p-c-Jun (S73) levels were detected in normal or steatotic donor livers (Left) and ND-, HFD- and CDHFD-fed mice livers (Right). Representative results were shown.

(**F** and **G**) PA induces ZBP1 expression in time-dependent manner. mRNA and protein levels of ZBP1 after different stimulation time of 0.4 mM PA in normal PMH (n = 5). Mean ± SD. One-way ANOVA, post hoc Dunnett's test.

(**H** and **I**) SP600125 abrogates PA-induced ZBP1 expression. mRNA and protein levels of ZBP1 after stimulation of 0.4 mM PA and 10μM SP600125 in normal PMH for 24 h (n = 5). Mean ± SD. One-way ANOVA, post hoc Dunnett's test.

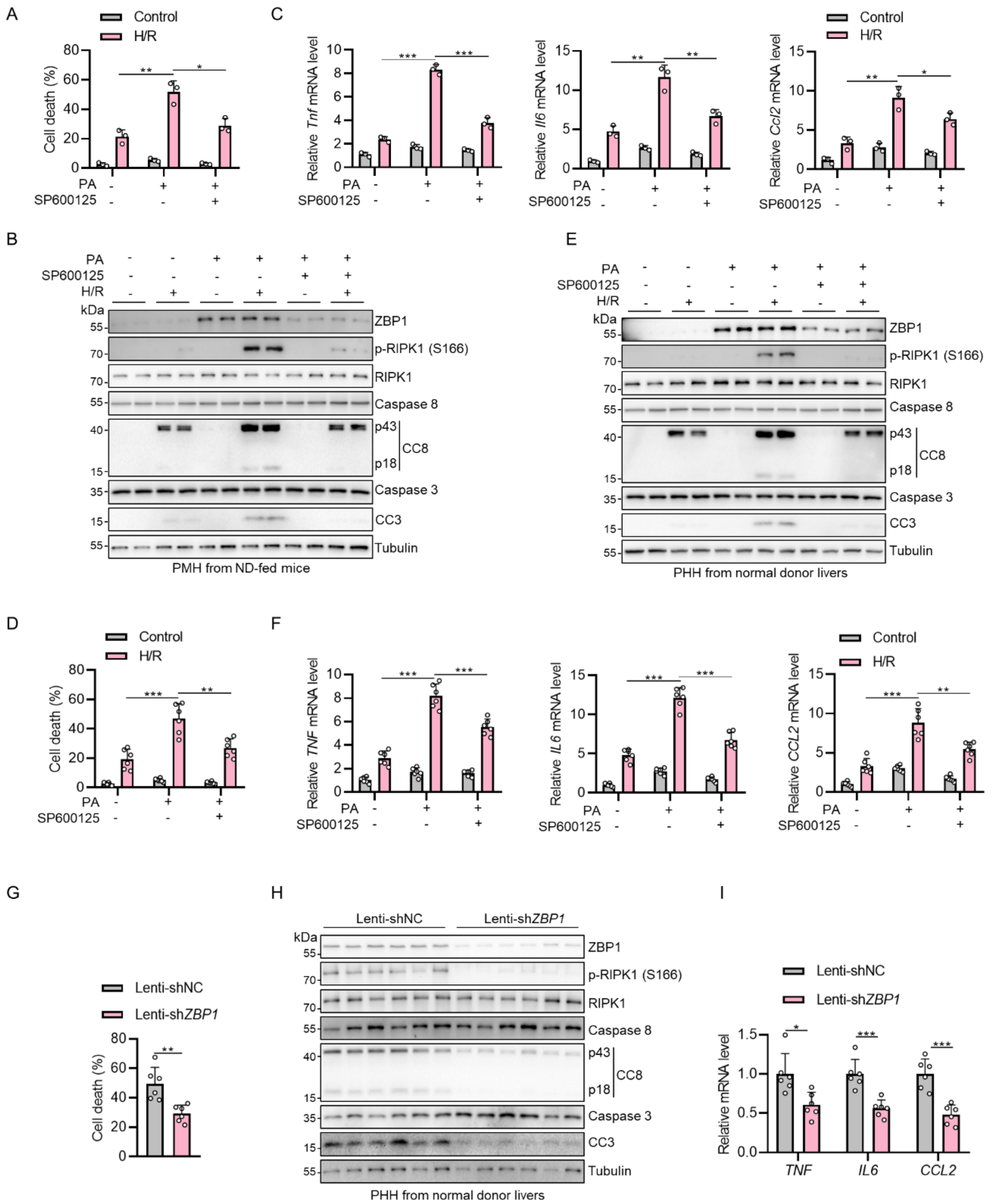

Supplemental Figure 12. Palmitic acid upregulates hepatocyte ZBP1 expression level through JNK pathway to exacerbate I/R injury of steatotic livers. Related to Figure 5.

(**A-C**) Pretreatment of PA aggravates cell death and inflammation in PMH H/R model which is reversed by SP600125. PMH from ND-fed mice were pretreated with 0.2 mM PA and 10 μM

SP600125 for 24 h and subjected to 10 h hypoxia. Cell death was measured with SytoxGreen positivity after 24 h reoxgenation (A). Cells were analyzed for p-RIPK1 (S166), CC8, CC3 with immunoblot after 3 h reoxygenation (B). Representative results were shown. mRNA levels of proinflammatory cytokines *Tnf*, *Il6*, *Ccl2* were detected with qRT-PCR after 3 h reoxygenation (C). Mean ± SD. n = 3 for each group. Two-way ANOVA, post hoc Bonferroni's test.

(**D-F**) Pretreatment of PA aggravates cell death and inflammation in PHH H/R model which is reversed by SP600125. PHH from normal donor livers were pretreated with 0.2 mM PA and 10 μM SP600125 for 24 h and subjected to 10 h hypoxia. Cell death was measured with SytoxGreen positivity after 24 h reoxgenation (D). Cells were analyzed for p-RIPK1 (S166), CC8, CC3 with immunoblot after 3 h reoxygenation (E). Representative results were shown. mRNA levels of proinflammatory cytokines *TNF*, *IL6*, *CCL2* were detected with qRT-PCR after 3 h reoxygenation (F). Mean ± SD. n = 3 for each group. Two-way ANOVA, post hoc Bonferroni's test.

(**G-I**) ZBP1 exacerbates cell death and inflammation in cell H/R model after PA stimulation. PHH were isolated from normal donor livers. *ZBP1* was knocked down with lentivirus 48 h before PA treatment. After 24 h treatment with 0.2 mM PA, the cells were exposed to 10 h hypoxia. Cell death was measured with SytoxGreen positivity after 24 h reoxygenation (G). ZBP1, p-RIPK1, CC8, CC3 protein levels were analyzed after 3 h reoxgenation (H). mRNA levels of proinflammatory cytokines *TNF*, *IL6*, *CCL2* were detected after 3 h reoxgenation (I). Mean ± SD. n = 6 for each group. Unpaired two-tailed Student's t-test (G). Two-way ANOVA, post hoc Bonferroni's test (I).

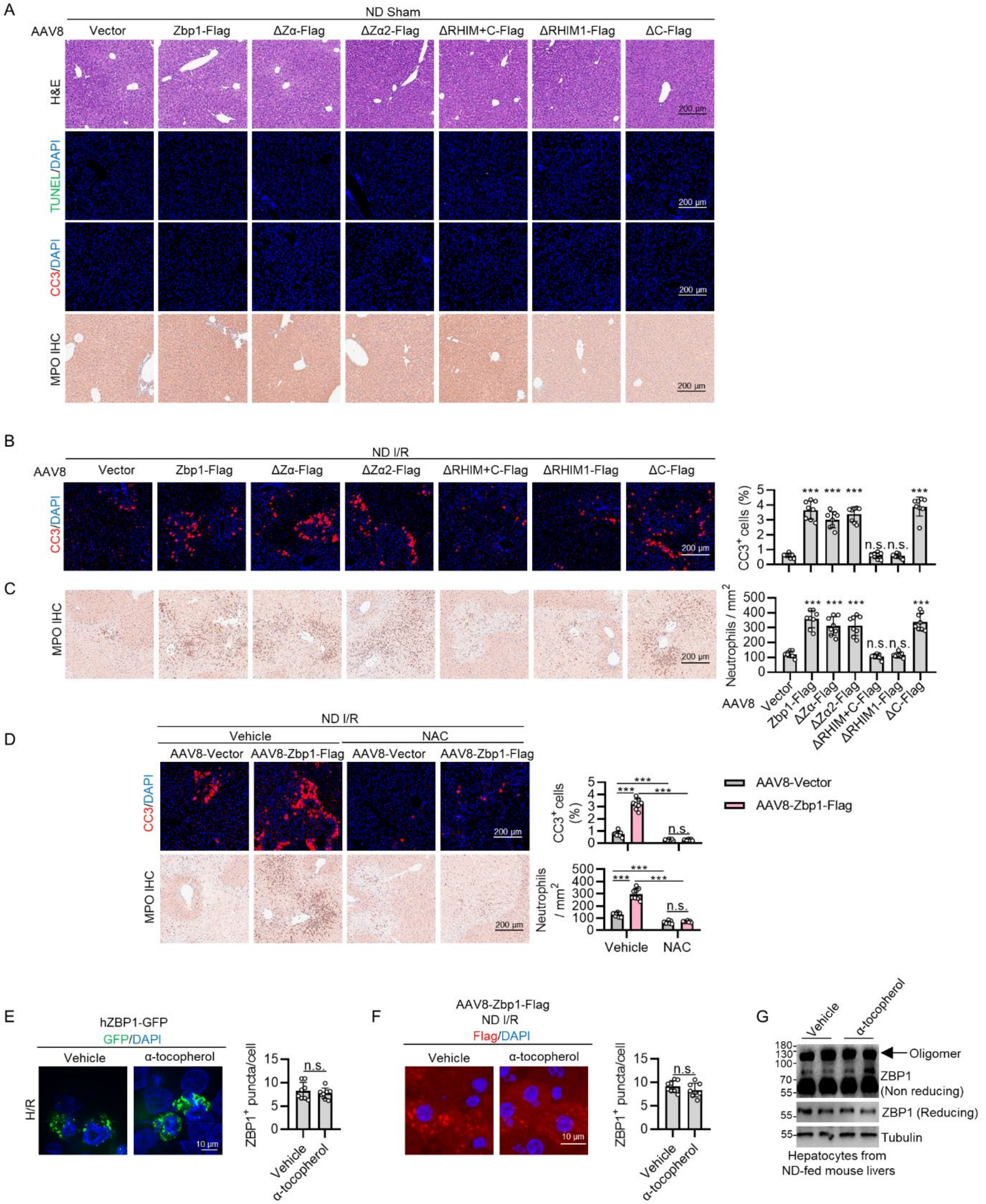

Supplemental Figure 13. ROS promotes ZBP1 aggregation and activation in liver I/R injury. Related to Figure 7.

(**A**) Overexpression of truncation mutants of ZBP1 does not trigger spontaneous cell death.

Different truncation mutants of ZBP1 were packaged into AAV8. 2×10^{11} v.g. in 200 µl of AAV8 were injected to ND-fed WT mice via the tail vein to overexpress ZBP1. After 1 month, the mice were subjected to sham operation. Liver injury was analyzed with H&E staining, TUNEL staining, CC3 immunostaining, MPO IHC. Representative images out of $n = 4$ mice for each group were shown.

(**B** and **C**) RHIM1 domain is indispensible for ZBP1-mediated apoptosis and inflammation. Different truncation mutants of ZBP1 were packaged into AAV8. 2×10^{11} v.g. in 200 µl of AAV8 were injected to the ND-fed WT mice via the tail vein. 1 month after AAV8 injection, the mice were subjected to 1 h ischemia/6 h reperfusion operation. Apoptosis was detected with CC3 immunostaining (B). Neutrophil infiltration was analyzed with MPO IHC (C). Mean ± SD. $n = 8$ mice for each group. One-way ANOVA, post hoc Dunnett's test.

(**D**) ROS triggers ZBP1 aggregation and activation in liver I/R injury. 2×10^{11} v.g. in 200 µl of AAV8 overexpressing ZBP1 was injected to ND-fed WT mice. After 1 month, the mice were intraperitoneally injected with 600 mg/kg NAC. After 1 h, the mice were exposed to 1 h ischemia/6 h reperfusion operation. Liver injury was analyzed with CC3 immunostaining and MPO IHC. Mean ± SD. $n = 8$ mice for each group. Two-way ANOVA, post hoc Bonferroni's test.

(**E**) Lipid ROS does not significantly contribute to hZBP1 aggregation in H/R model. hZBP1-GFP palasmids were transfected into HEK293T cells. After pretreatment with 20 µM α-tocopherol for 1 h, the cells were subjected to H/R challenge and ZBP1 oligomers were detected from green fluorescence. Mean ± SD. $n = 8$ mice for each group. Unpaired two-tailed Student's t-test.

(**F** and **G**) Lipid ROS does not significantly contribute to ZBP1 aggregation in liver I/R injury.

ZBP1-Flag was packaged into AAV8 and 2×10^{11} v.g. in 200 μl of AAV8 were injected to the ND-fed WT mice via the tail vein. 1 month after AAV8 injection, 50 mg/kg α-tocopherol was intraperitoneally injected into the mice. After 1 h, the mice were exposed to 1 h ischemia/6 h reperfusion operation. ZBP1 aggregation was analyzed from Flag immunostaining (F) or immunoblot under non reducing conditions (G). Mean ± SD. $n = 8$ mice for each group. Unpaired two-tailed Student's t-test.

Supplemental Table 1. Basic characteristics of the donors.

Characteristics	Steatotic liver		P value
	No (n=25)	Yes (n=24)	
**Gender**			0.906
Male	15 (60.0%)	14 (58.3%)	
Female	10 (40.0%)	10 (41.7%)	
**Age, *y***	47.2 (27.3-69.5)	45.5 (23.7-73.4)	0.179
**Height, *cm***	168.6 (157.2-183.0)	169.4 (158.8-178.2)	0.208
**Weight, *kg***	67.5 (32.0-92.0)	78.1 (57.2-94.5)	<0.001
**Macrosteatosis**	0	21.7% (0%-60.0%)	<0.001
**Pretransplant laboratory values**			0.225
White blood cell	11.9 (3.5-23.3)	11.5 (0.2-23.0)	0.466
Red blood cell	3.9 (2.5-5.3)	3.7 (1.7-5.7)	0.298
Hemoglobin	116.0 (85.0-168.0)	116.0 (74.0-174.0)	0.779
Platelets	187.8 (53.0-456.0)	175.4 (24.0-266.0)	0.624
Lymphocyte	1.2 (0.2-8.9)	1.1 (0.1-2.3)	0.582
Mononuclear cell	0.7 (0.3-1.5)	0.8 (0.0-1.7)	0.231
Neutrophil	10.1 (2.8-20.0)	9.7 (0.0-20.7)	0.265
Eosinophilic cell	0.1 (0.0-0.5)	0.1 (0.0-0.3)	0.870
Basophil cell	0.1 (0.0-0.2)	0.1 (0.0-0.1)	0.631
Lymphocyte%	8.5 (1.9-16.8)	9.9 (2.2-36.7)	0.645
Monocyte%	6.5 (2.9-12.7)	6.5 (3.1-11.3)	0.575
Neutrophil%	83.7 (71.2-95.2)	80.6 (6.6-94.3)	0.124
Eosinophil%	1.1 (0.0-5.0)	0.8 (0.0-4.3)	0.375
Basophil%	0.2 (0.0-1.0)	0.2 (0.0-0.8)	0.473
C-reactive protein	65.3 (0.5-444.5)	67.4 (0.9-239.0)	0.541
PCT	9.2 (0.0-47.4)	19.0 (0.0-386.5)	0.103
alkaline phosphatase	72.2 (41.0-161.9)	85.3 (40.3-210.8)	0.608
Total protein	65.7 (45.8-83.0)	63.8 (37.5-87.8)	0.204
Albumen	38.1 (25.4-53.5)	37.9 (21.1-51.6)	0.213
Globulin	28.1 (16.9-44.1)	25.9 (16.4-36.2)	0.775
Albumin/Globulin	1.5 (0.7-2.6)	1.5 (1.0-2.1)	0.478
γ-glutamyl transferase	56.9 (7.2-491.0)	53.4 (8.0-179.0)	0.124
AST	46.7 (7.0-229.6)	54.6 (11.0-300.5)	0.283
ALT	55.1 (4.0-362.0)	59.6 (8.0-554.8)	0.529
Total bilirubin	14.4 (5.0-24.7)	14.2 (6.5-28.3)	0.232
Direct bilirubin	5.6 (0.1-12.5)	4.4 (0.0-17.9)	0.660
Total bile acid	3.9 (0.1-22.0)	3.0 (0.1-10.8)	0.369
Potassium	4.3 (2.9-5.3)	3.8 (2.5-5.3)	0.772
Urea nitrogen	11.1 (3.5-31.4)	9.5 (3.1-34.2)	0.246

Creatinine	114.8 (26.0-300.4)	115.8 (37.8-487.0)	0.207
Uric acid	256.0 (76.0-491.0)	274.4 (8.6-793.0)	0.501
PT	13.6 (9.4-20.4)	13.9 (9.4-18.3)	0.575
APTT	37.5 (24.8-99.6)	33.8 (22.0-47.8)	0.196
D-dimer	327.9 (0.0-6515.9)	360.7 (0.1-7670.0)	0.447
TT	18.1 (9.1-90.0)	16.0 (10.3-20.5)	0.833
INR	1.2 (0.9-1.9)	1.2 (0.9-1.6)	NA
**Hepatitis B surface antigen**			
No	25 (100%)	24 (100%)	
Yes	0 (0)	0 (0)	

Supplemental Table 2. Basic characteristics of the recipients.

Characteristics	Steatotic liver		P value
	No (n=25)	Yes (n=24)	
**Gender**			0.897
Male	14 (60.0%)	13 (58.3%)	
Female	11 (40.0%)	11 (41.7%)	0.168
**Age, *y***	46.4 (25.5-72.2)	47.5 (24.5-75.7)	0.223
**Height, *cm***	169.4 (158.2-182.3)	167.5 (157.8-181.2)	0.746
**Weight, *kg***	66.2 (37.8-85.0)	77.1 (36.4-88.7)	0.952
**Disease etiology**			
Hepatitis B virus	6 (24.0%)	5 (20.8%)	
Hepatitis C virus	2 (8.0%)	2 (8.3%)	
Alcohol	2 (8.0%)	1 (4.2%)	
Malignant tumor	10 (40.0%)	12 (50.0%)	
Others	5 (20.0 %)	4 (16.7%)	0.680
**Hepatocellular carcinoma**			
With	10 (40.0%)	11 (45.8%)	
Without	15 (60.0%)	13 (54.2%)	
**Pretransplant laboratory values**			0.253
White blood cell	12.3 (3.7-24.8)	11.7 (1.3-21.9)	0.426
Red blood cell	4.0 (2.3-5.5)	3.5 (1.9-4.6)	0.289
Hemoglobin	107.0 (86.1-174.2)	112.5 (73.2-181.6)	0.742
Platelets	165.7 (54.6-473.3)	154.7 (20.6-255.4)	0.631
Lymphocyte	1.3 (0.4-7.5)	1.2 (0.1-2.5)	0.565
Mononuclear cell	0.6 (0.4-1.3)	0.9 (0.1-1.6)	0.287
Neutrophil	11.2 (3.7-23.4)	10.6 (0.0-19.3)	0.303
Eosinophilic cell	0.1 (0.0-0.4)	0.1 (0.0-0.2)	0.805
Basophil cell	0.1 (0.0-0.1)	0.1 (0.0-0.3)	0.675
Lymphocyte%	7.9 (2.2-17.3)	10.3 (3.1-34.5)	0.624
Monocyte%	2.7 (3.5-12.5)	7.2 (4.4-13.1)	0.537
Neutrophil%	82.5 (75.1-92.6)	82.7 (5.3-97.2)	0.141
Eosinophil%	1.4 (0.3-4.8)	0.9 (0.3-5.2)	0.304
Basophil%	0.4 (0.1-1.4)	0.3 (0.0-0.7)	0.096
C-reactive protein	63.6 (0.7-457.3)	65.7 (1.7-251.9)	0.508
PCT	9.4 (0.0-44.7)	17.6 (0.0-375.4)	0.178
alkaline phosphatase	72.2 (41.0-161.9)	85.3 (40.3-210.8)	0.624
Total protein	67.4 (43.0-86.2)	66.9 (34.9-85.3)	0.283
Albumen	33.1 (26.2-52.0)	36.8 (21.1-53.7)	0.253
Globulin	27.4 (16.9-46.2)	26.9 (18.1-34.1)	0.142

Albumin/Globulin	1.6 (0.9-2.8)	1.7 (1.2-2.5)	0.478
γ-glutamyl transferase	59.6 (8.1-485.9)	55.8 (8.6-216.0)	0.698
AST	47.4 (7.2-236.4)	57.1 (13.1-310.3)	0.284
ALT	57.3 (3.8-357.4)	62.5 (8.9-538.4)	0.557
Total bilirubin	15.1 (5.3-26.3)	14.7 (8.5-29.7)	0.260
Direct bilirubin	6.4 (0.1-13.4)	4.8 (0.0-15.8)	0.634
Total bile acid	4.2 (0.2-21.3)	3.5 (0.1-12.6)	0.397
Potassium	4.3 (2.9-7.2)	3.8 (2.4-6.5)	0.725
Urea nitrogen	14.1 (4.7-34.2)	8.3 (5.3-36.1)	0.259
Creatinine	128.4 (25.6-304.0)	125.4 (33.7-464.8)	0.214
Uric acid	263.9 (72.3-482.2)	327.3 (47.5-673.3)	0.147
PT	15.7 (8.9-23.5)	13.2 (8.1-17.4)	0.559
APTT	41.3 (27.4-68.9)	37.4 (19.3-57.8)	0.272
D-dimer	572.8 (0.1-5645.9)	486.3 (0.1-6345.0)	0.483
TT	20.5 (13.1-37.6)	18.6 (11.3-24.6)	0.827
INR	1.3 (0.8-1.8)	1.2 (1.0-1.6)	
