# Supplementary material for: ZBP1-mediated apoptosis and inflammation exacerbate steatotic liver ischemia/reperfusion injury
Source: J Clin Invest. 2024 May 14;134(13):e180451. doi: 10.1172/JCI180451 (PMC11213514; doi:10.1172/JCI180451)
Supplement: Unedited blot and gel images [file jci-134-180451-s067.pdf]

Figure 1

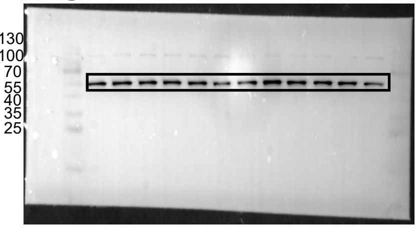

Figure 1E left Caspase 8

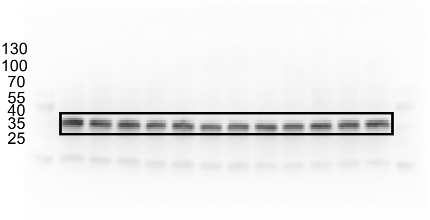

Figure 1E right Caspase 3

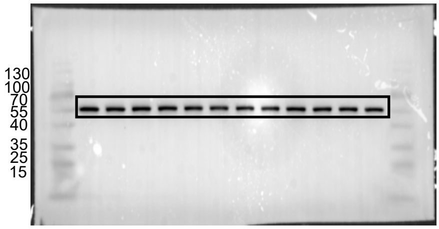

Figure 1G left Tubulin

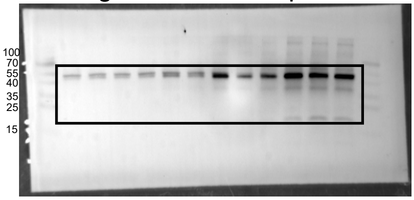

Figure 1E left CC8

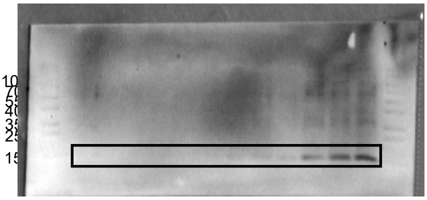
Figure 1E right CC3

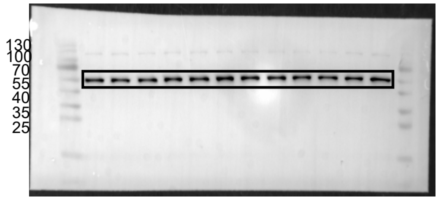

Figure 1G right Caspase 8

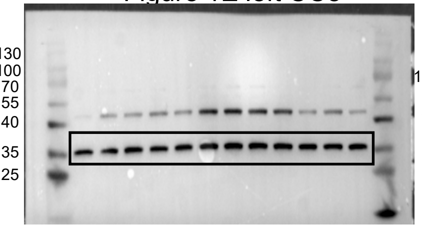

Figure 1E left Caspase 3

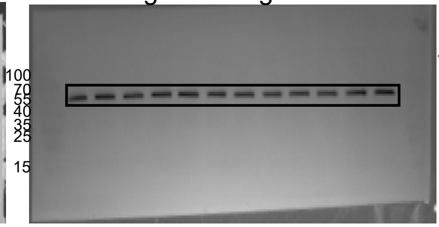

Figure 1E right Tubulin

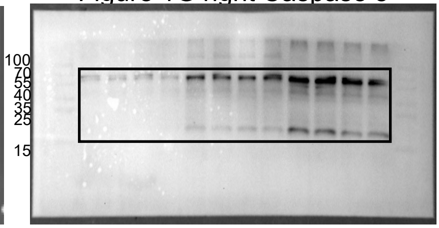

Figure 1G right CC8

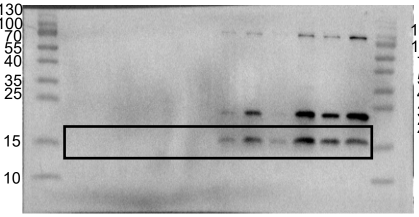

Figure 1E left CC3

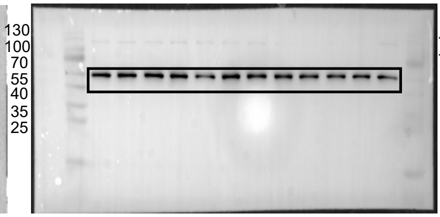

Figure 1G left Caspase 8

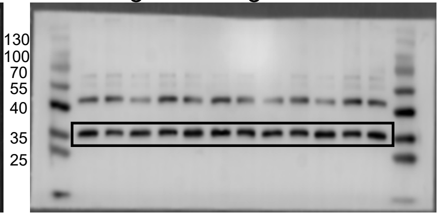

Figure 1G right Caspase 3

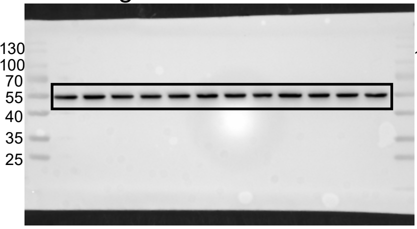

Figure 1E left Tubulin

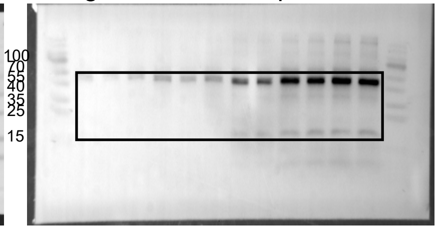

Figure 1G left CC8

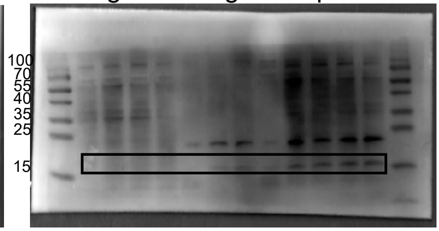

Figure 1G right CC3

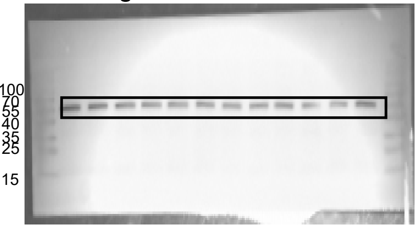

Figure 1E right Caspase 8

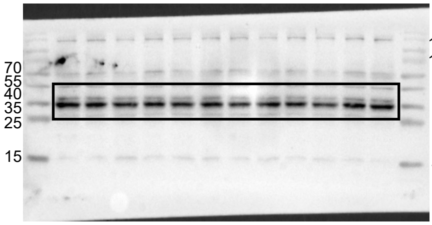

Figure 1G left Caspase 3

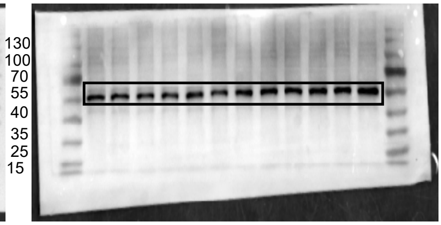

Figure 1G right Tubulin

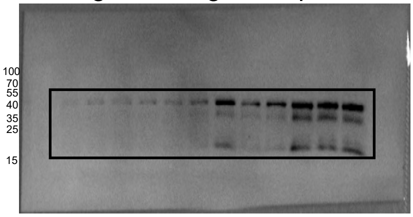

Figure 1E right CC8

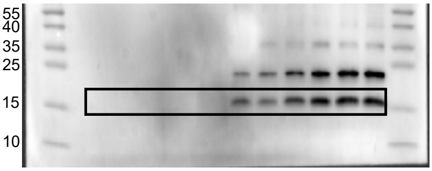

Figure 1G left CC3

Figure 2

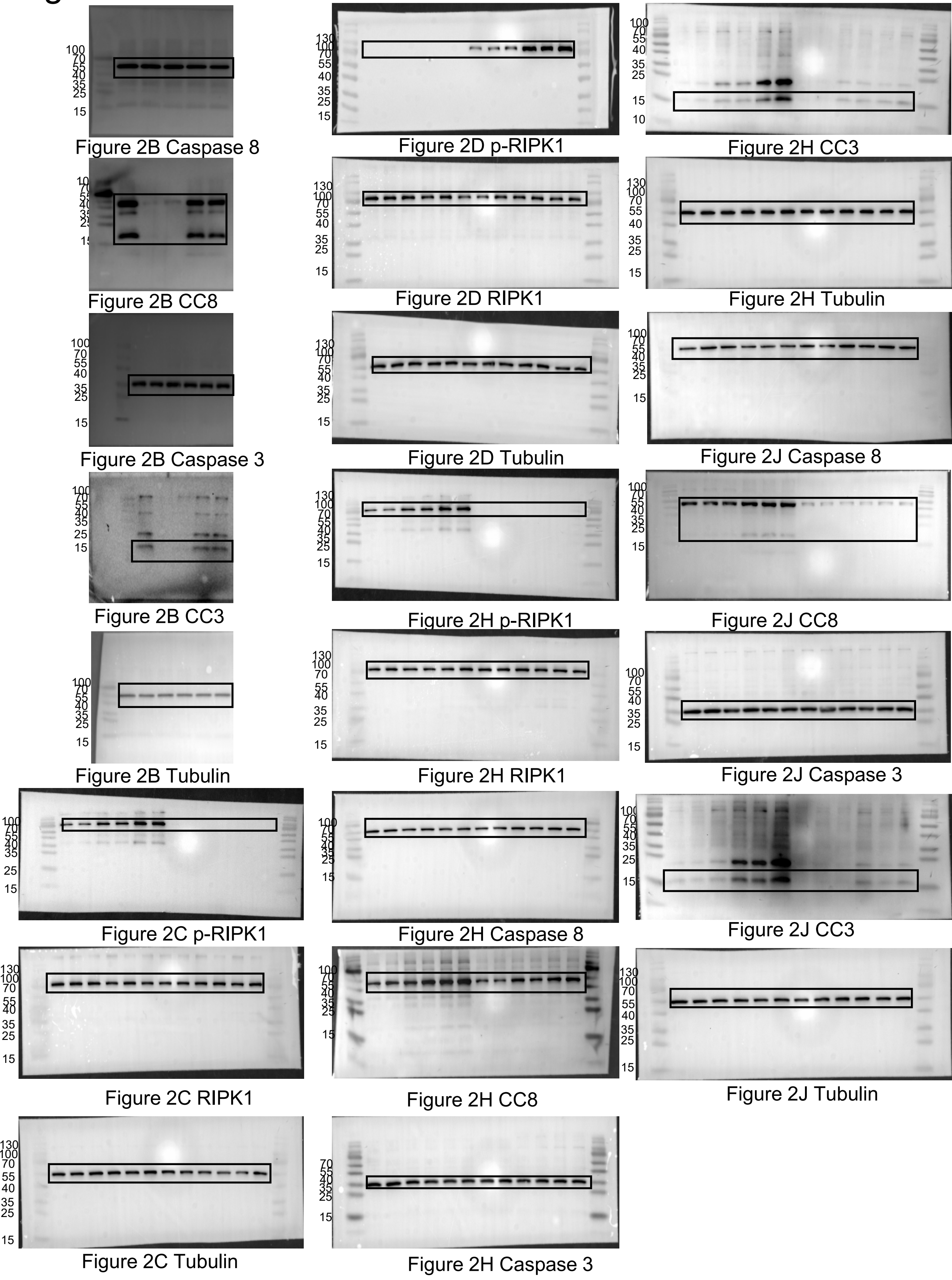
Figure 2B Caspase 8
Figure 2D p-RIPK1
Figure 2H CC3
Figure 2B CC8
Figure 2D RIPK1
Figure 2H Tubulin
Figure 2B Caspase 3
Figure 2D Tubulin
Figure 2J Caspase 8
Figure 2B CC3
Figure 2H p-RIPK1
Figure 2J CC8
Figure 2B Tubulin
Figure 2H RIPK1
Figure 2J Caspase 3
Figure 2C p-RIPK1
Figure 2H Caspase 8
Figure 2J CC3
Figure 2C RIPK1
Figure 2H CC8
Figure 2J Tubulin
Figure 2C Tubulin
Figure 2H Caspase 3

Figure 3

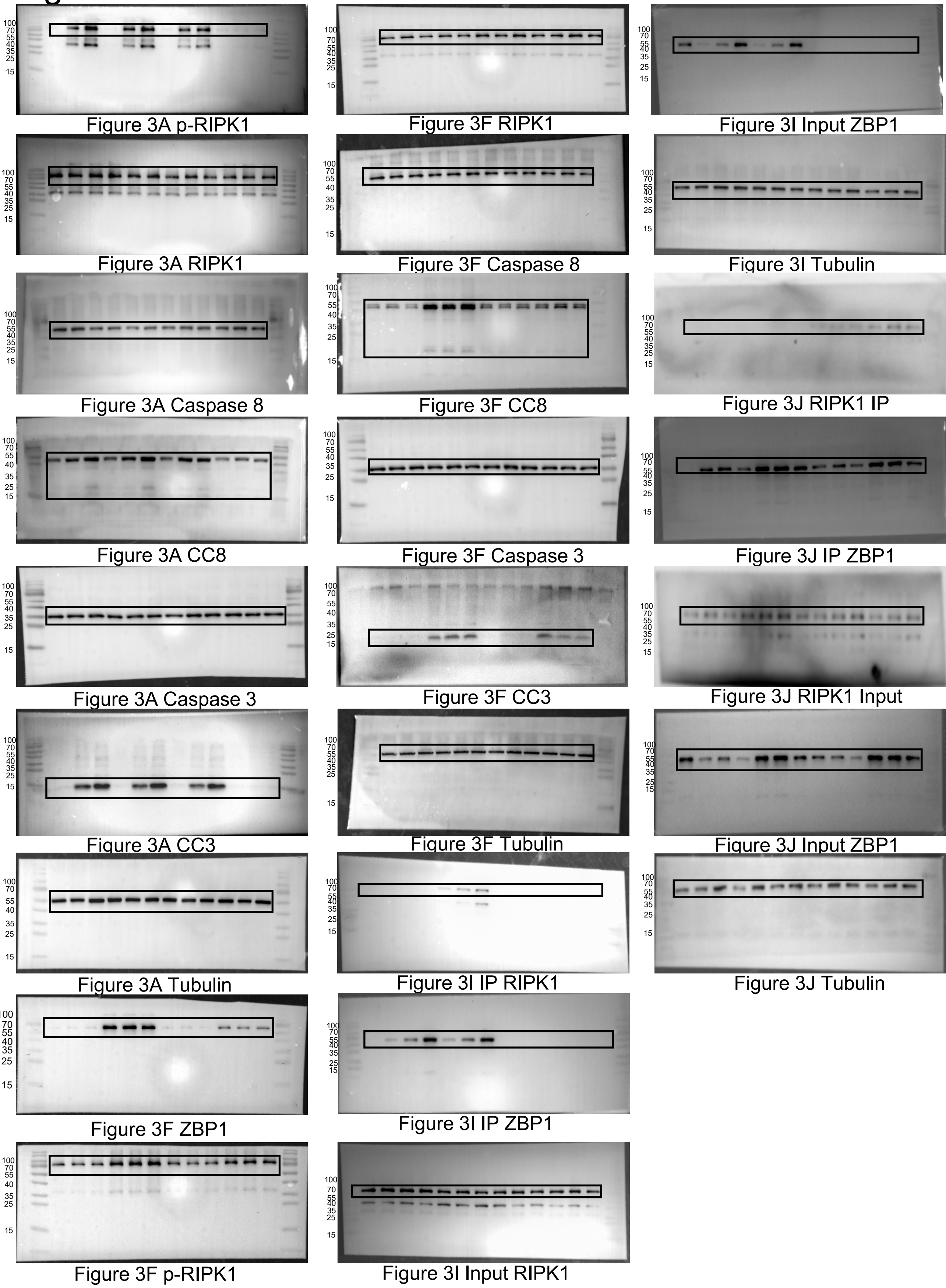
Figure 3A p-RIPK1
Figure 3F RIPK1
Figure 3I Input ZBP1
Figure 3A RIPK1
Figure 3F Caspase 8
Figure 3I Tubulin
Figure 3A Caspase 8
Figure 3F CC8
Figure 3J RIPK1 IP
Figure 3A CC8
Figure 3F Caspase 3
Figure 3J IP ZBP1
Figure 3A Caspase 3
Figure 3F CC3
Figure 3J RIPK1 Input
Figure 3A CC3
Figure 3F Tubulin
Figure 3J Input ZBP1
Figure 3A Tubulin
Figure 3I IP RIPK1
Figure 3J Tubulin
Figure 3F ZBP1
Figure 3I IP ZBP1
Figure 3F p-RIPK1
Figure 3I Input RIPK1

Figure 4

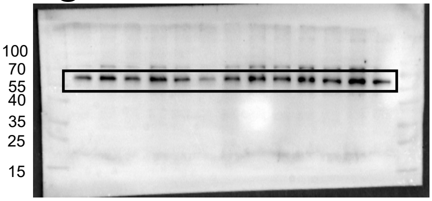

Figure 4A ZBP1 1

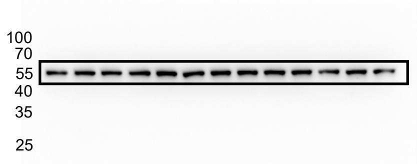

Figure 4A Tubulin 1

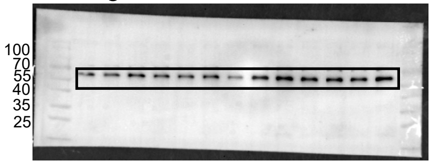

Figure 4A ZBP1 2

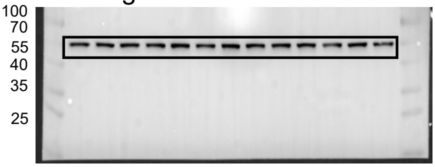

Figure 4A Tubulin 2

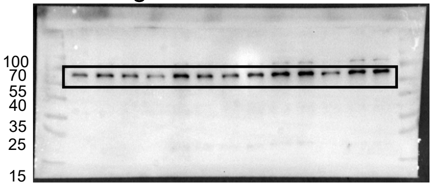

Figure 4A ZBP1 3

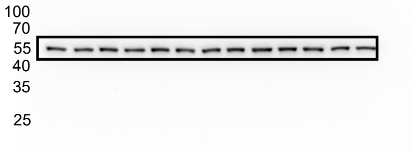

Figure 4A Tubulin 3

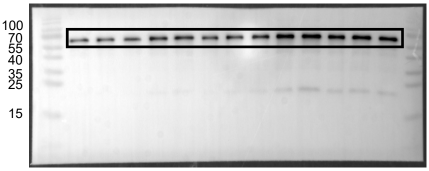

Figure 4A ZBP1 4

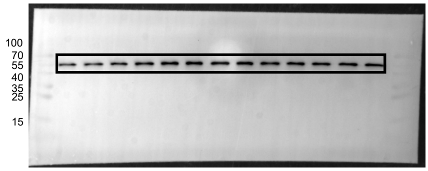

Figure 4A Tubulin 4

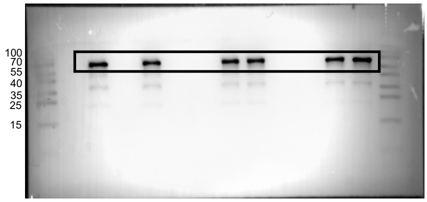

Figure 4F Flag

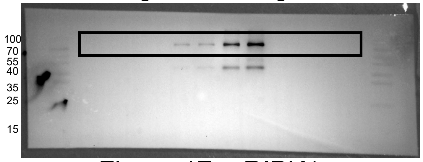

Figure 4F p-RIPK1

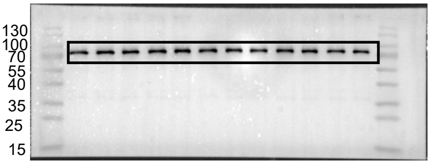

Figure 4F RIPK1

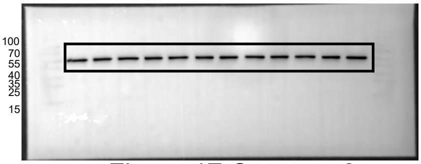

Figure 4F Caspase 8

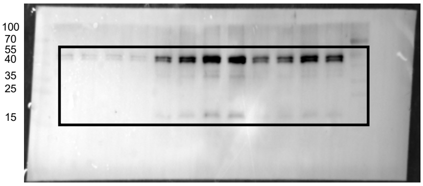

Figure 4F CC8

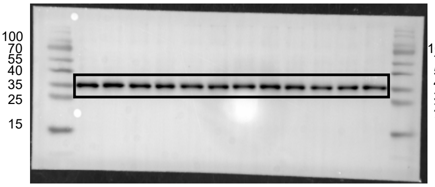

Figure 4F Caspase 3

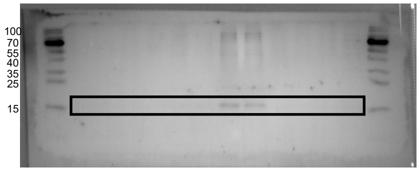

Figure 4F CC3

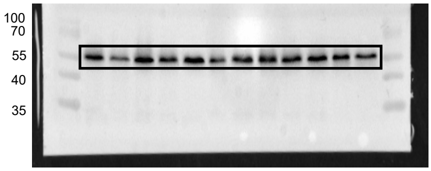

Figure 4F Tubulin

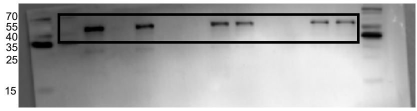

Figure 4J Flag

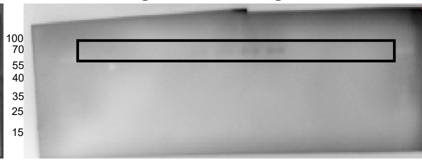

Figure 4J p-RIPK1

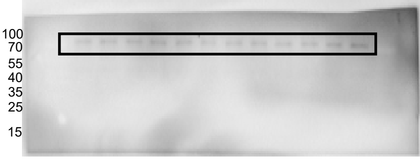

Figure 4J RIPK1

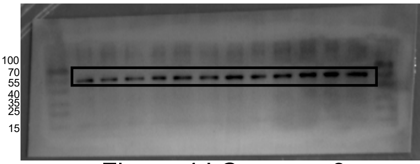

Figure 4J Caspase 8

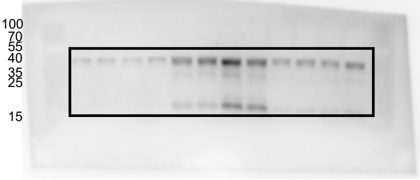

Figure 4J CC8

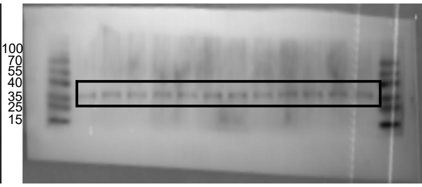

Figure 4J Caspase 3

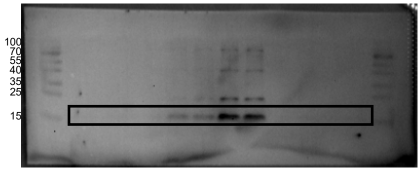

Figure 4J CC3

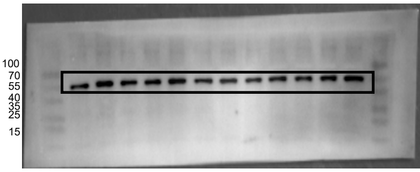

Figure 4J Tubulin

Figure 5

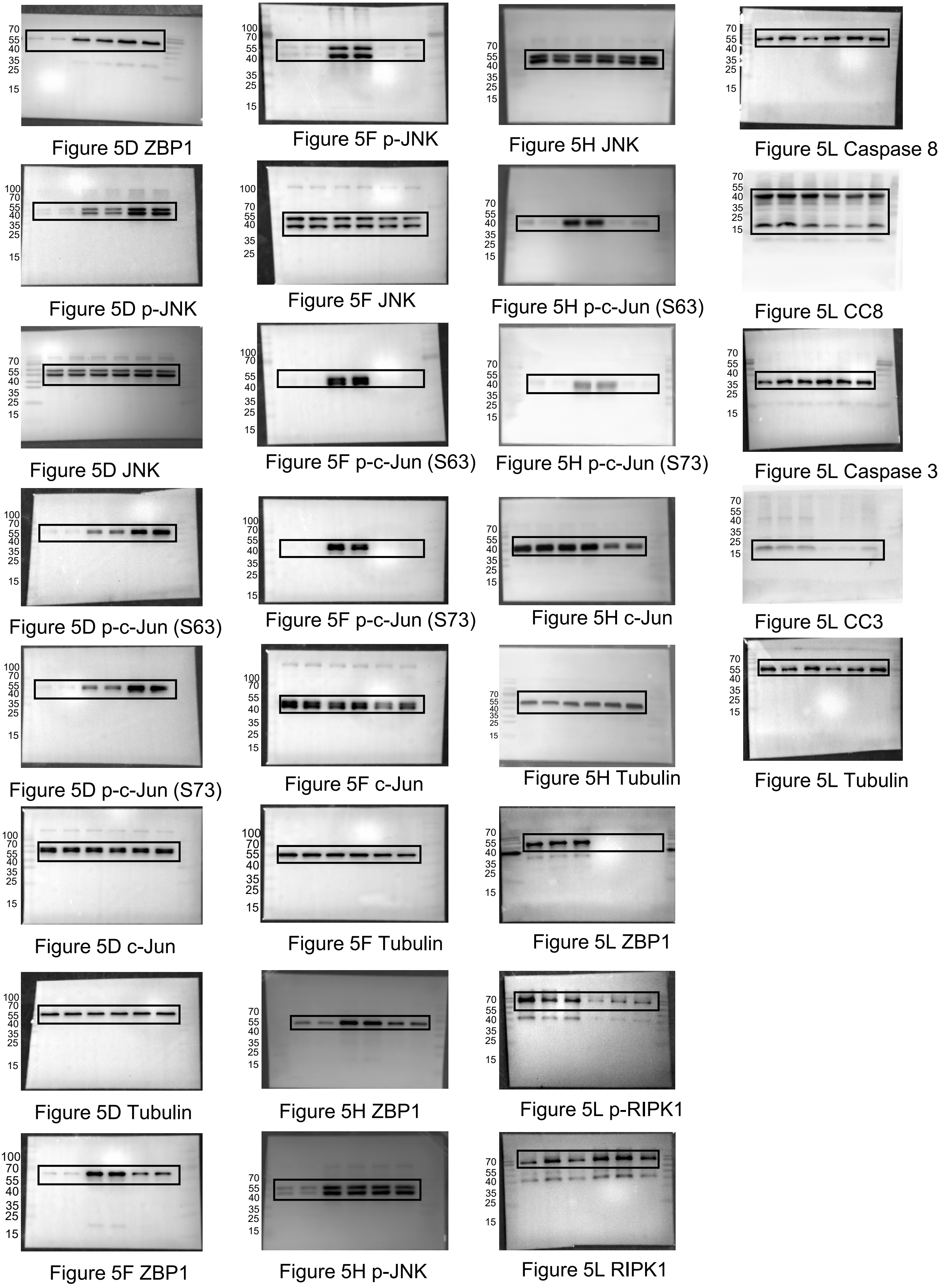

Figure 5D ZBP1
Figure 5F p-JNK
Figure 5H JNK
Figure 5L Caspase 8
Figure 5D p-JNK
Figure 5F JNK
Figure 5H p-c-Jun (S63)
Figure 5L CC8
Figure 5D JNK
Figure 5F p-c-Jun (S63)
Figure 5H p-c-Jun (S73)
Figure 5L Caspase 3
Figure 5D p-c-Jun (S63)
Figure 5F p-c-Jun (S73)
Figure 5H c-Jun
Figure 5L CC3
Figure 5D p-c-Jun (S73)
Figure 5F c-Jun
Figure 5H Tubulin
Figure 5L Tubulin
Figure 5D c-Jun
Figure 5F Tubulin
Figure 5L ZBP1
Figure 5D Tubulin
Figure 5H ZBP1
Figure 5L p-RIPK1
Figure 5F ZBP1
Figure 5H p-JNK
Figure 5L RIPK1

Figure 6

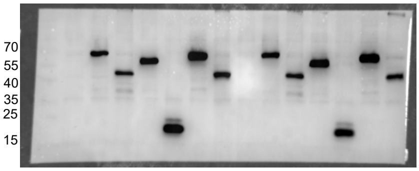

Figure 6B IP Flag

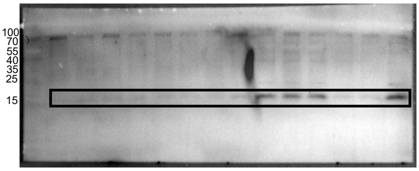

Figure 6B CC3

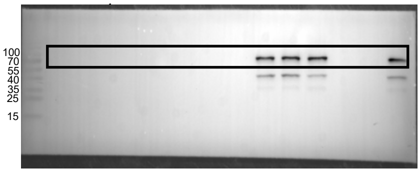

Figure 6B IP RIPK1

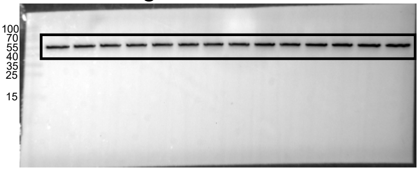

Figure 6B Tubulin

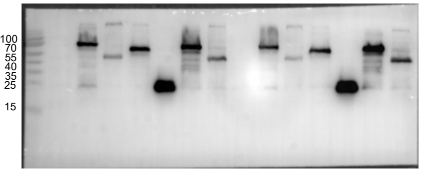

Figure 6B Input Flag

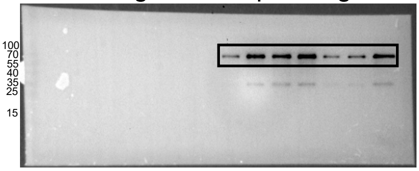

Figure 6B p-RIPK1

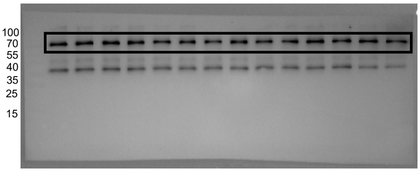

Figure 6B Input RIPK1

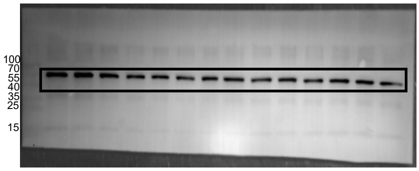

Figure 6B Caspase 8

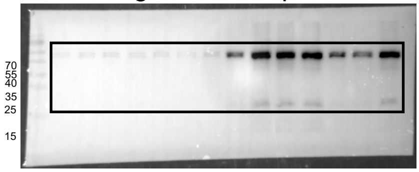

Figure 6B CC8

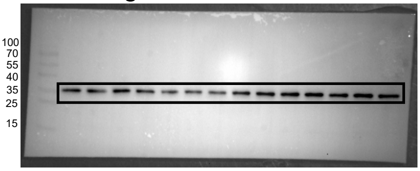

Figure 6B Caspase 3

Figure 7

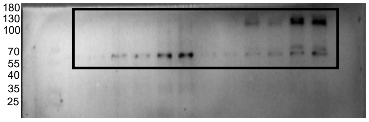

Figure 7B left ZBP1 (Non reducing)

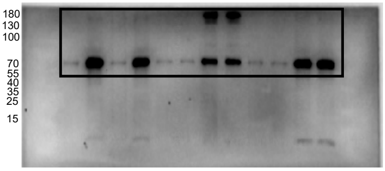

Figure 7E ZBP1(Non reducing)

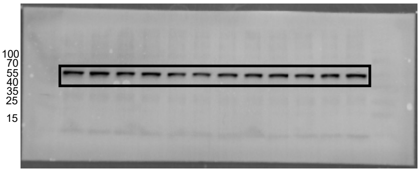

Figure 7E Tubulin

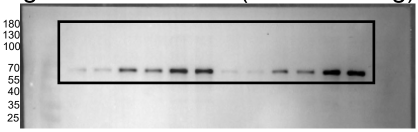

Figure 7B left ZBP1 (Reducing)

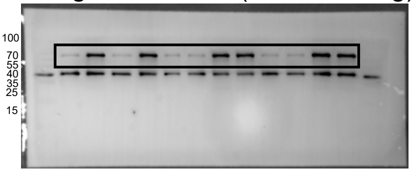

Figure 7E ZBP1(Reducing)

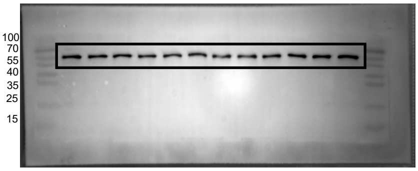

Figure 7B left Tubulin

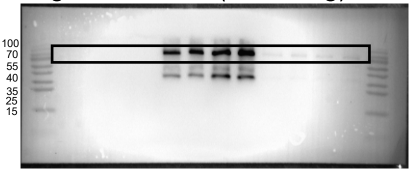

Figure 7E p-RIPK1

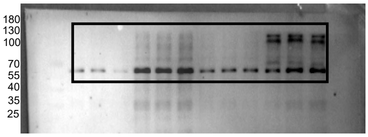

Figure 7B right ZBP1 (Non reducing)

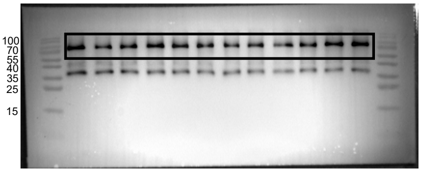

Figure 7E RIPK1 Input

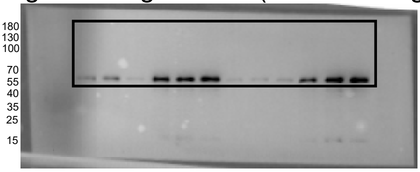

Figure 7B right ZBP1 (Reducing)

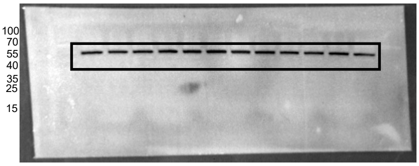

Figure 7E Caspase 8

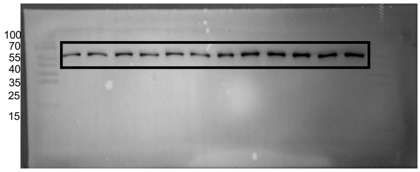

Figure 7B right Tubulin

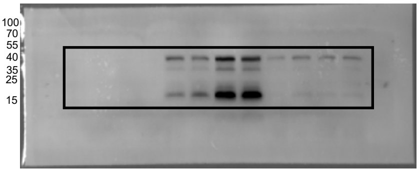

Figure 7E CC8

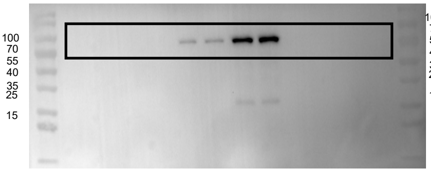

Figure 7E RIPK1 IP

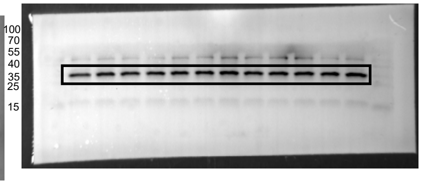

Figure 7E Caspase 3

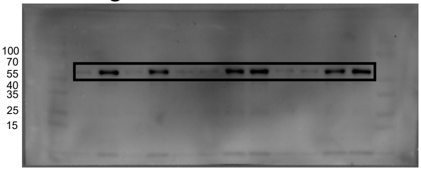

Figure 7E ZBP1 IP

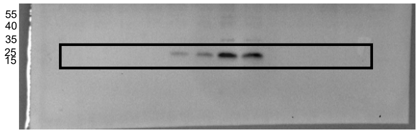

Figure 7E CC3

Supplemental Figure 1

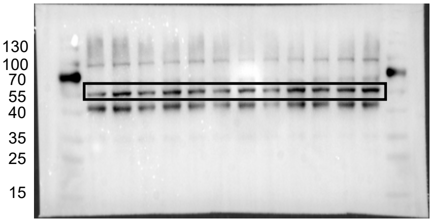

Supplemental Figure 1C FL-GSDMD

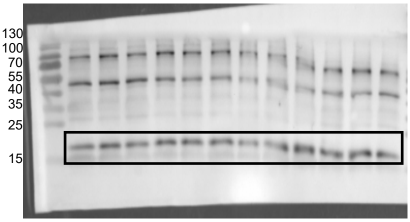

Supplemental Figure 1C GPX4

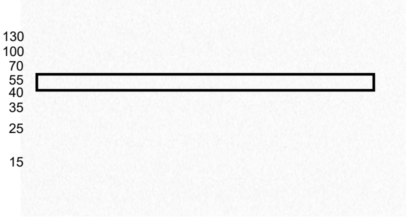

Supplemental Figure 1D p-RIPK3

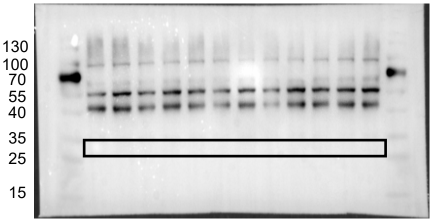

Supplemental Figure 1C N-GSDMD

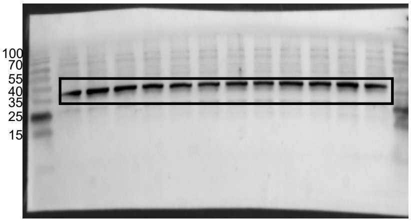

Supplemental Figure 1C FSP1

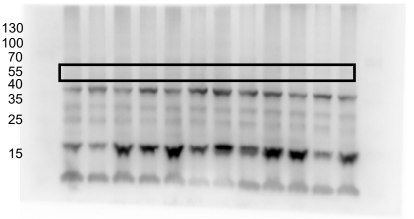

Supplemental Figure 1D RIPK3

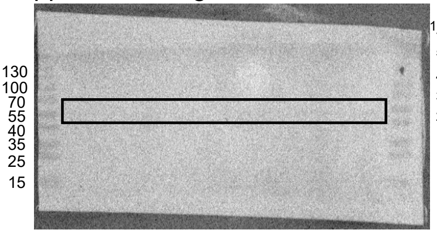

Supplemental Figure 1C p-RIPK3

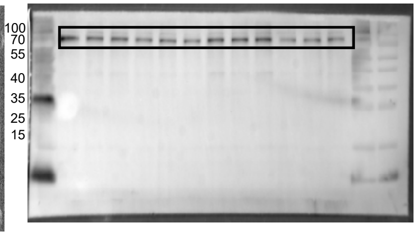

Supplemental Figure 1C ACSL4

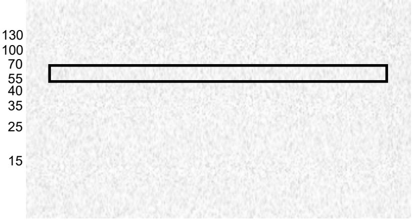

Supplemental Figure 1D p-MLKL

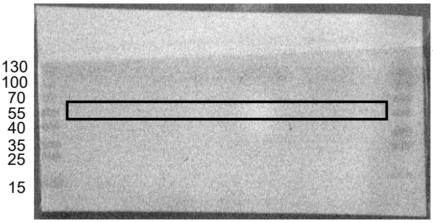

Supplemental Figure 1C RIPK3

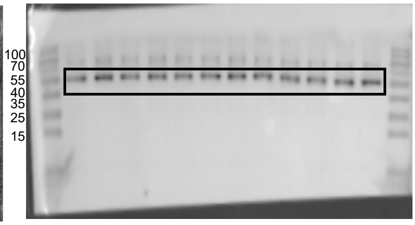

Supplemental Figure 1C Tubulin

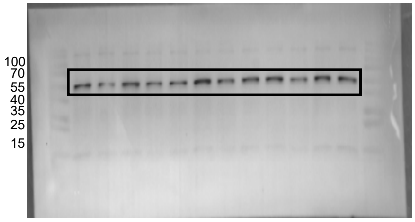

Supplemental Figure 1D MLKL

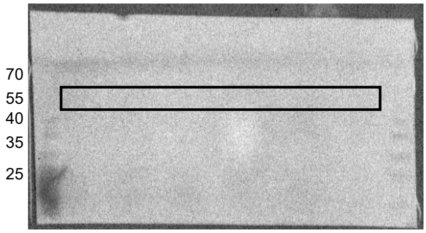

Supplemental Figure 1C p-MLKL

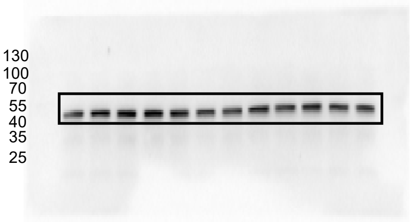

Supplemental Figure 1D FL-GSDMD

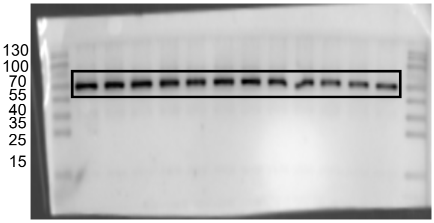

Supplemental Figure 1D Tubulin

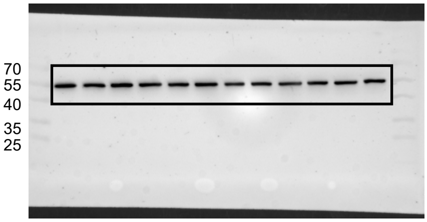

Supplemental Figure 1C MLKL

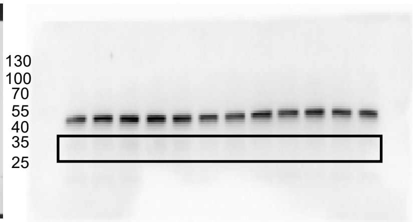

Supplemental Figure 1D N-GSDMD

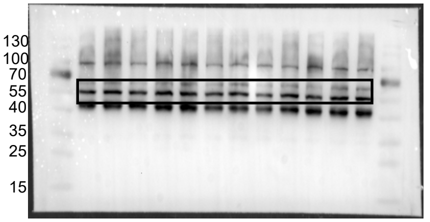

Supplemental Figure 1H FL-GSDMD

Supplemental Figure 1

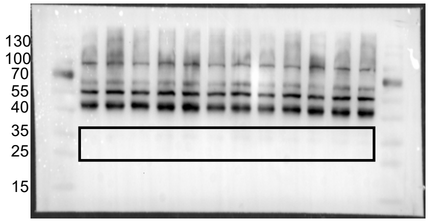

Supplemental Figure 1H N-GSDMD

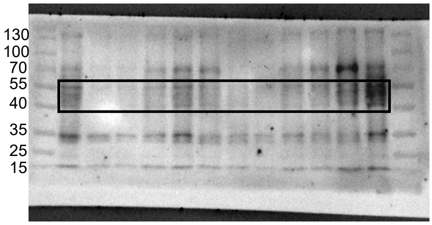

Supplemental Figure 1H p-RIPK3

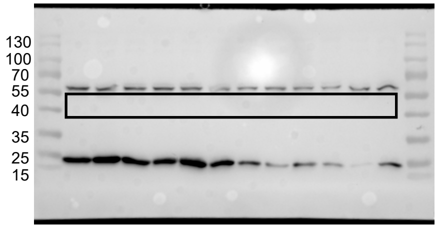

Supplemental Figure 1H RIPK3

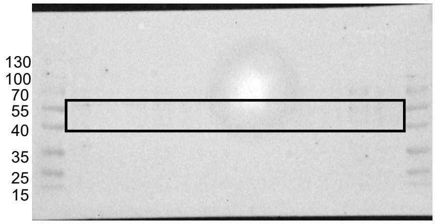

Supplemental Figure 1H p-MLKL

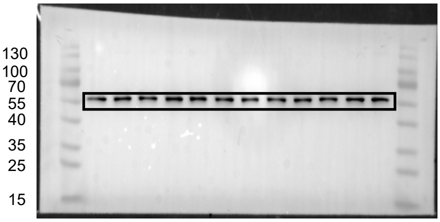

Supplemental Figure 1H MLKL

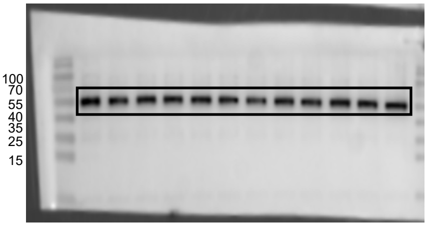

Supplemental Figure 1H Tubulin

Supplemental Figure 2

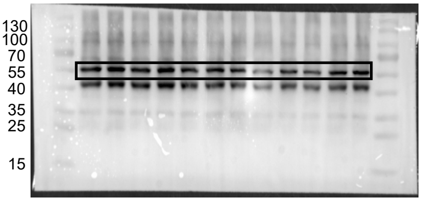

Supplemental Figure 2C FL-GSDMD

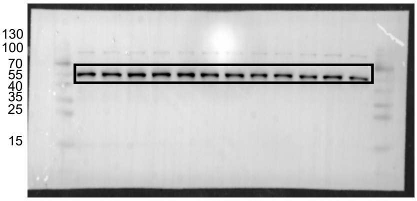

Supplemental Figure 2H Caspase 8

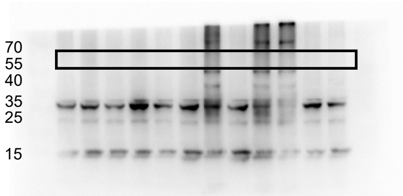

Supplemental Figure 2H RIPK3

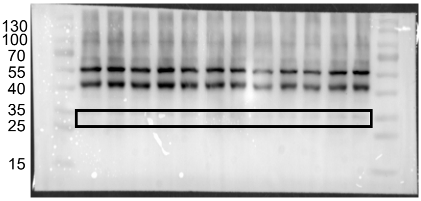

Supplemental Figure 2C N-GSDMD

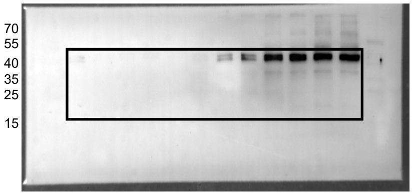

Supplemental Figure 2H CC8

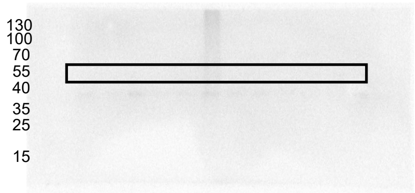

Supplemental Figure 2H p-MLKL

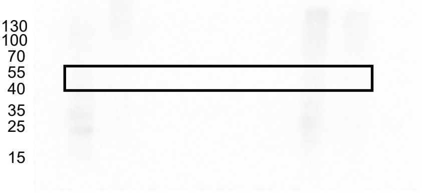

Supplemental Figure 2C p-RIPK3

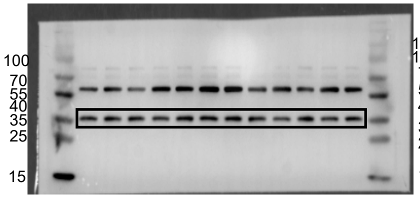

Supplemental Figure 2H Caspase 3

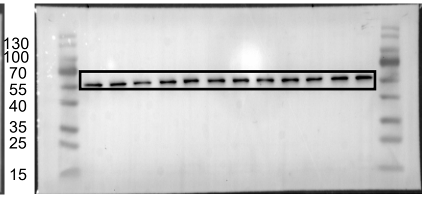

Supplemental Figure 2H MLKL

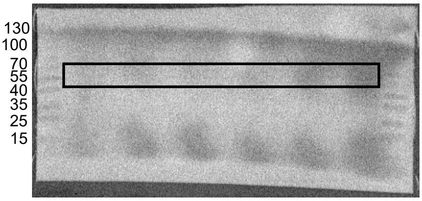

Supplemental Figure 2C RIPK3

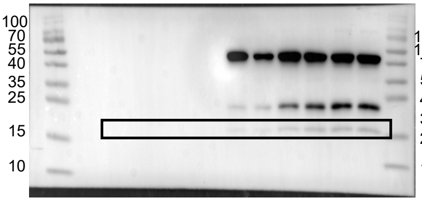

Supplemental Figure 2H CC3

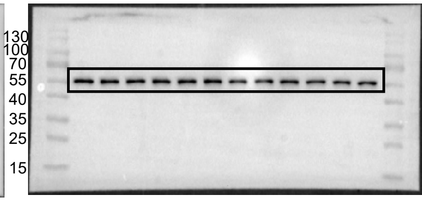

Supplemental Figure 2H Tubulin

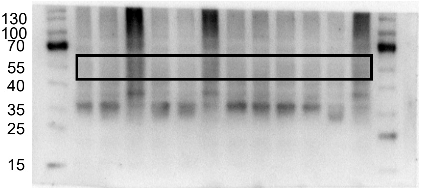

Supplemental Figure 2C p-MLKL

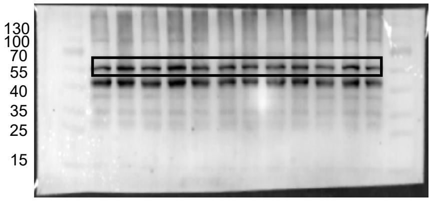

Supplemental Figure 2H FL-GSDMD

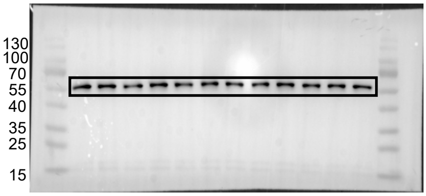

Supplemental Figure 2C MLKL

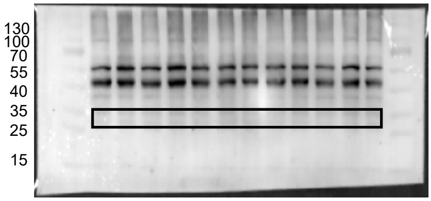

Supplemental Figure 2H N-GSDMD

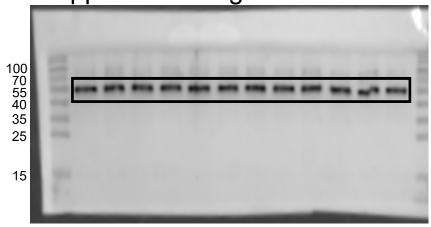

Supplemental Figure 2C Tubulin

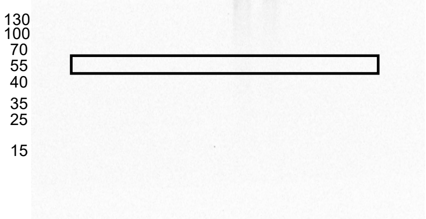

Supplemental Figure 2H p-RIPK3

Supplemental Figure 3

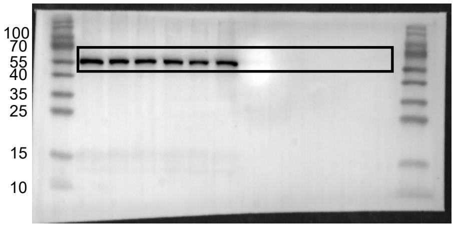

Supplemental Figure 3D Caspase 8

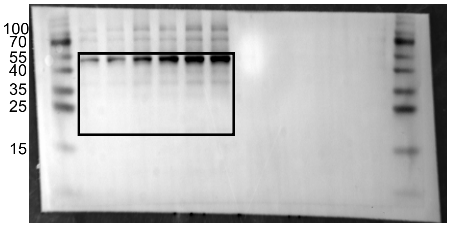

Supplemental Figure 3D CC8

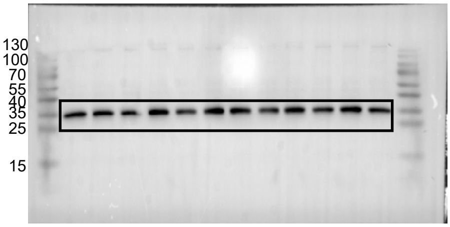

Supplemental Figure 3D Caspase 3

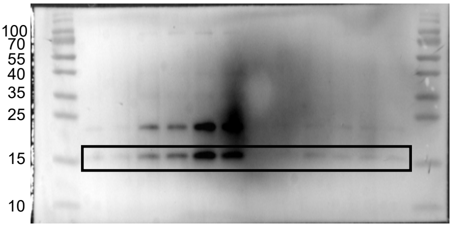

Supplemental Figure 3D CC3

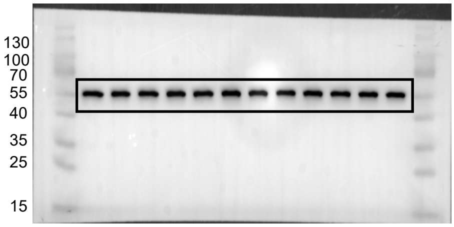

Supplemental Figure 3D Tubulin

Supplemental Figure 4

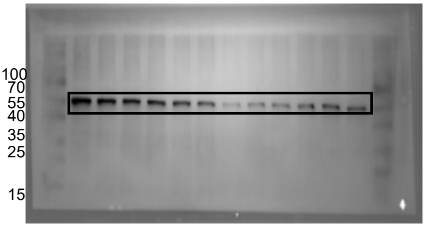

Supplemental Figure 4C Caspase 8

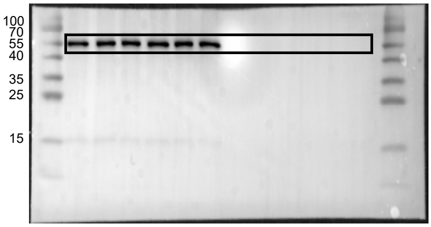

Supplemental Figure 4J Caspase 8

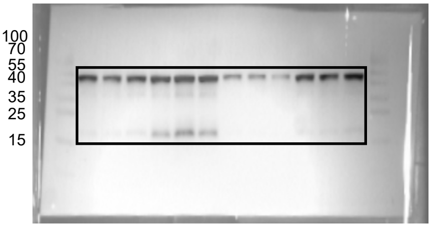

Supplemental Figure 4C CC8

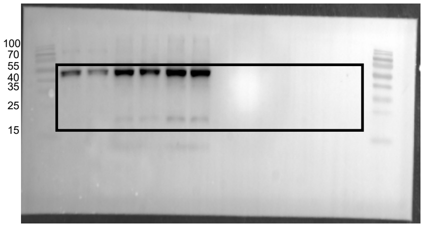

Supplemental Figure 4J CC8

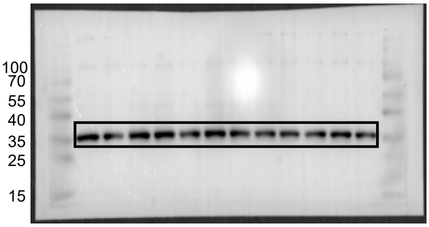

Supplemental Figure 4C Caspase 3

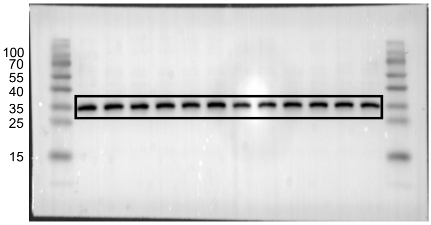

Supplemental Figure 4J Caspase 3

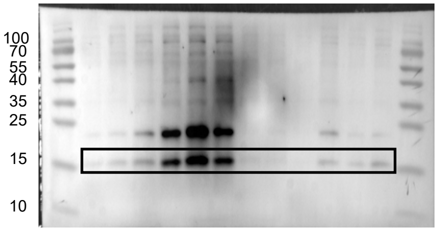

Supplemental Figure 4C CC3

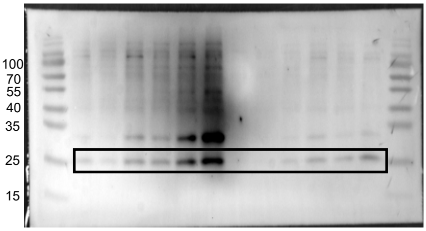

Supplemental Figure 4J CC3

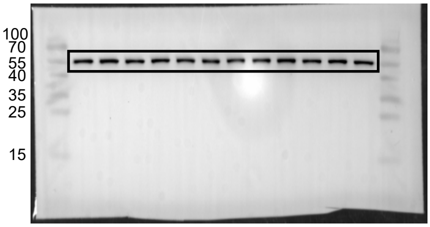

Supplemental Figure 4C Tubulin

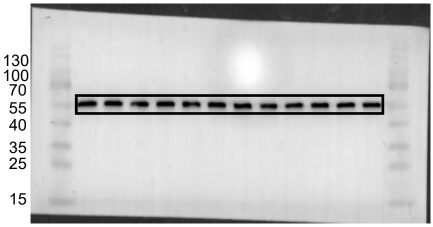

Supplemental Figure 4J Tubulin

Supplemental Figure 5

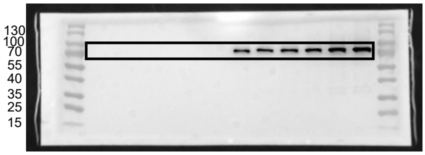

Supplemental Figure 5B p-RIPK1

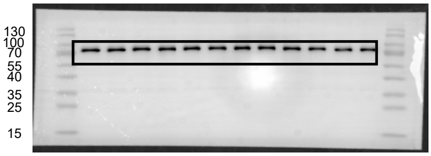

Supplemental Figure 5B RIPK1

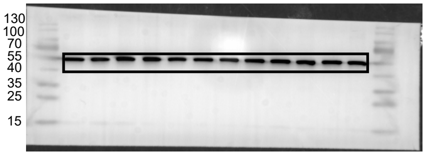

Supplemental Figure 5B Tubulin

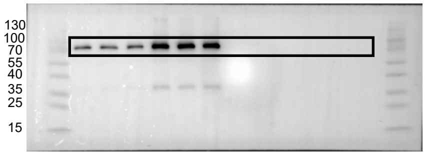

Supplemental Figure 5C p-RIPK1

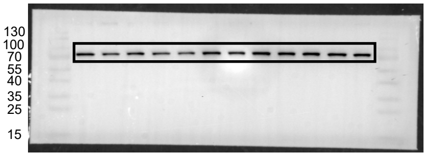

Supplemental Figure 5C RIPK1

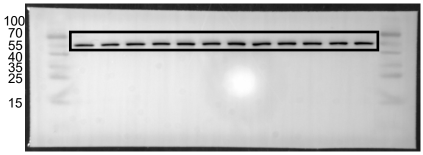

Supplemental Figure 5C Tubulin

Supplemental Figure 6

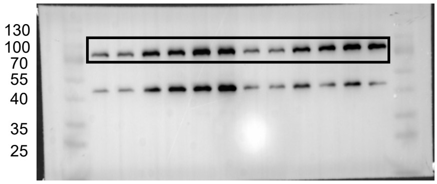

Supplemental Figure 6B p-RIPK1

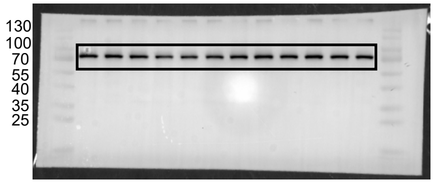

Supplemental Figure 6B RIPK1

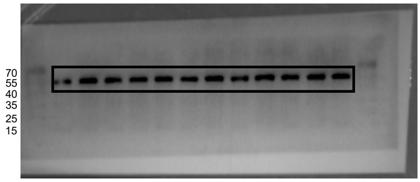

Supplemental Figure 6B Caspase 8

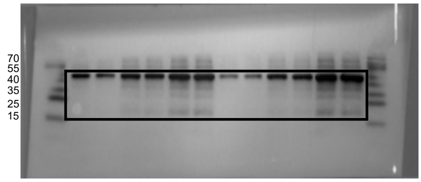

Supplemental Figure 6B CC8

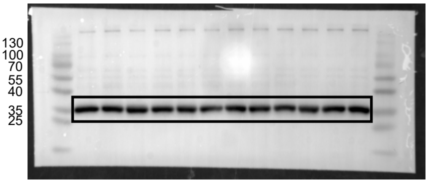

Supplemental Figure 6B Caspase 3

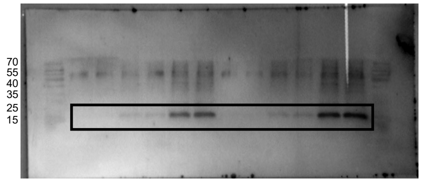

Supplemental Figure 6B CC3

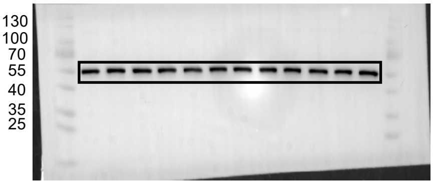

Supplemental Figure 6B Tubulin

Supplemental Figure 7

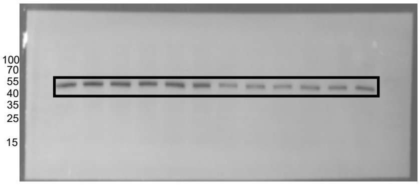

Supplemental Figure 7D TNFR1

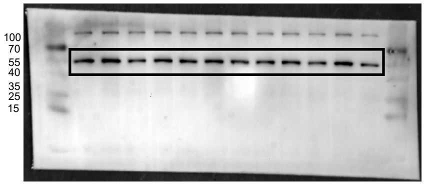

Supplemental Figure 7D Tubulin

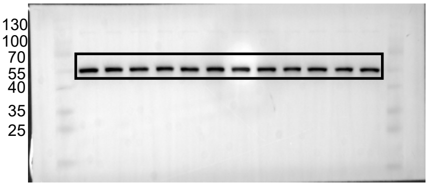

Supplemental Figure 7I Tubulin

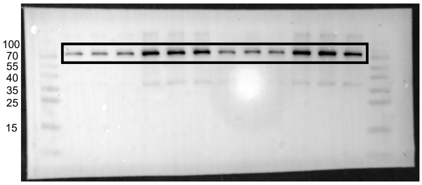

Supplemental Figure 7D p-RIPK1

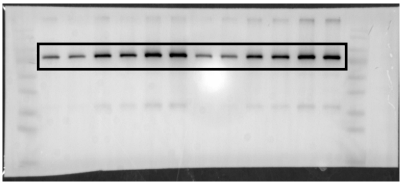

Supplemental Figure 7I p-RIPK1

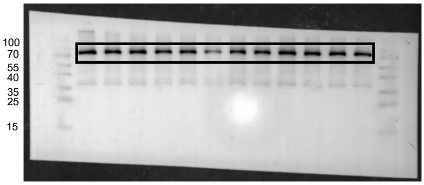

Supplemental Figure 7D RIPK1

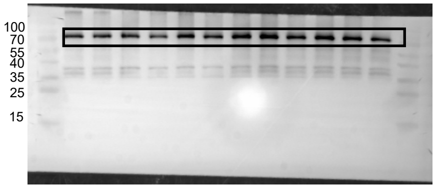

Supplemental Figure 7I RIPK1

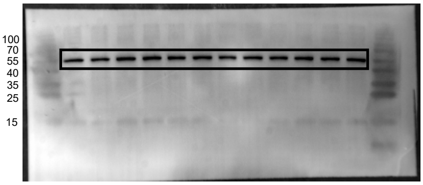

Supplemental Figure 7D Caspase 8

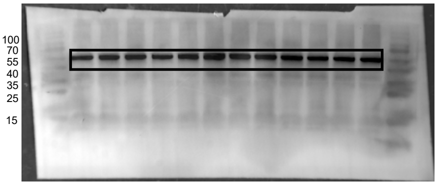

Supplemental Figure 7I Caspase 8

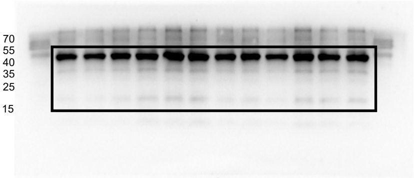

Supplemental Figure 7D CC8

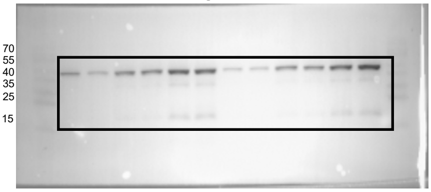

Supplemental Figure 7I CC8

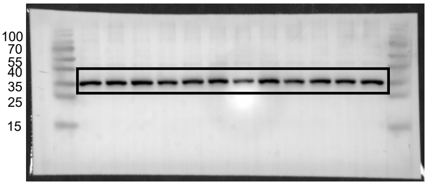

Supplemental Figure 7D Caspase 3

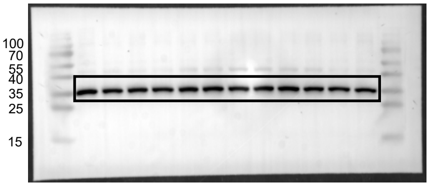

Supplemental Figure 7I Caspase 3

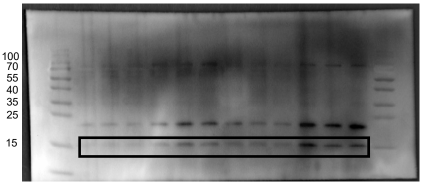

Supplemental Figure 7D CC3

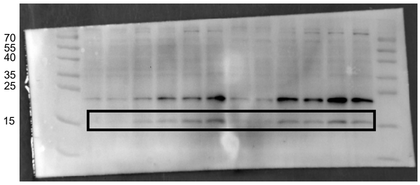

Supplemental Figure 7I CC3

Supplemental Figure 8

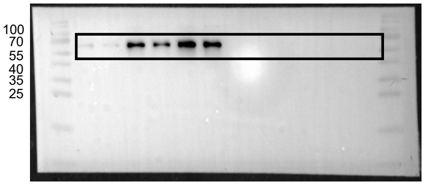

Supplemental Figure 8B ZBP1

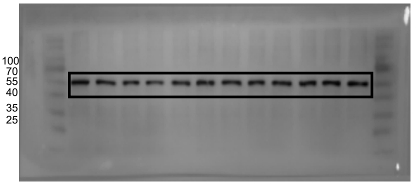

Supplemental Figure 8B Tubulin

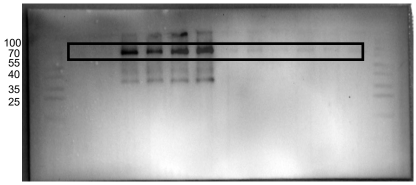

Supplemental Figure 8B p-RIPK1

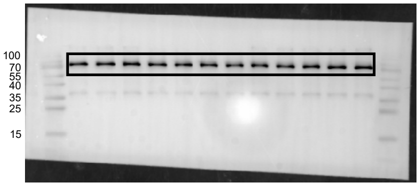

Supplemental Figure 8B RIPK1

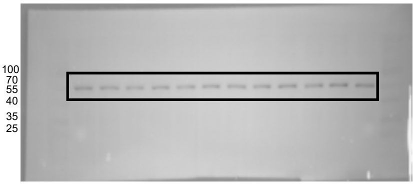

Supplemental Figure 8B Caspase 8

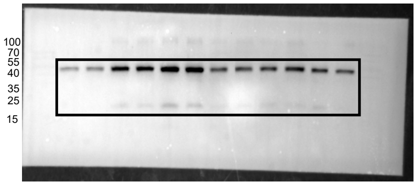

Supplemental Figure 8B CC8

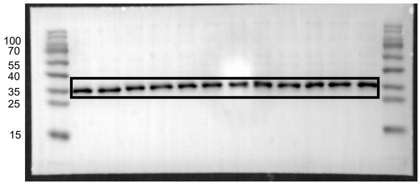

Supplemental Figure 8B Caspase 3

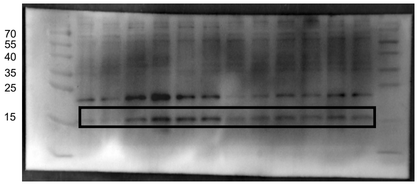

Supplemental Figure 8B CC3

Supplemental Figure 11

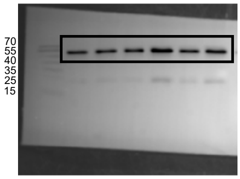

Supplemental Figure 11E left ZBP1

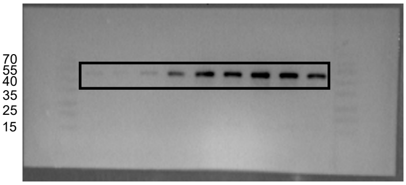

Supplemental Figure 11E right ZBP1

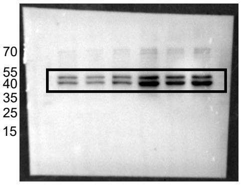

Supplemental Figure 11E left p-JNK

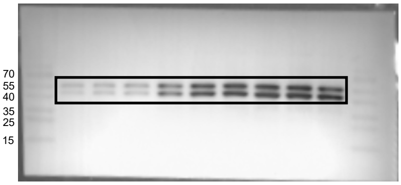

Supplemental Figure 11E right p-JNK

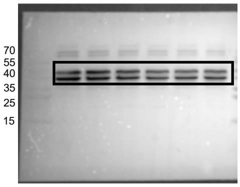

Supplemental Figure 11E left JNK

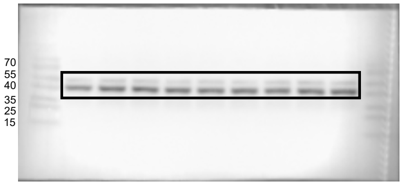

Supplemental Figure 11E right JNK

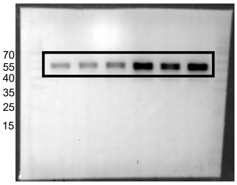

Supplemental Figure 11E left p-c-Jun(S63)

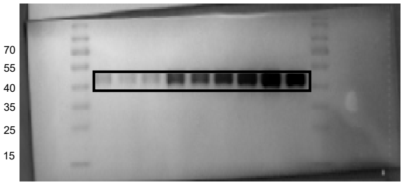

Supplemental Figure 11E right p-c-Jun(S63)

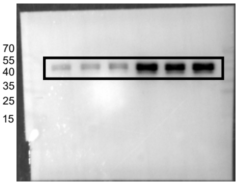

Supplemental Figure 11E left p-c-Jun(S73)

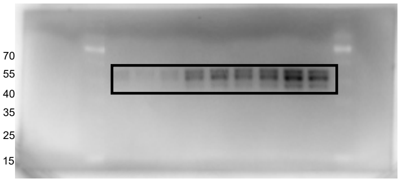

Supplemental Figure 11E right p-c-Jun(S73)

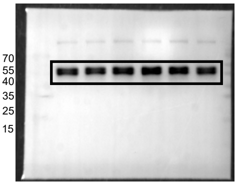

Supplemental Figure 11E left c-Jun

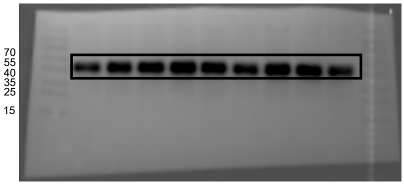

Supplemental Figure 11E right c-Jun

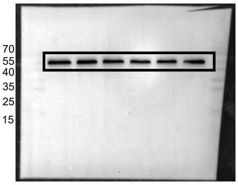

Supplemental Figure 11E left Tubulin

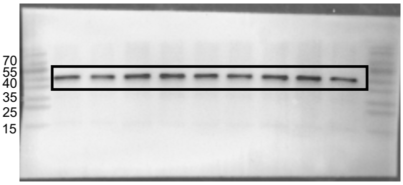

Supplemental Figure 11E right Tubulin

Supplemental Figure 11

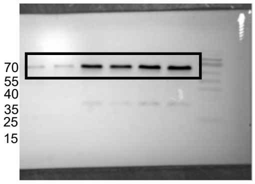

Supplemental Figure 11F ZBP1

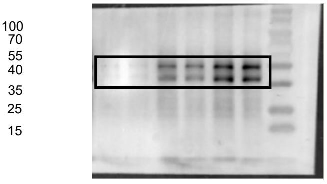

Supplemental Figure 11F p-JNK

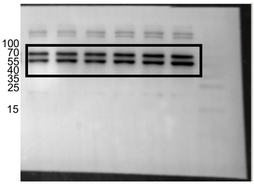

Supplemental Figure 11F JNK

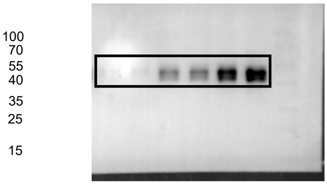

Supplemental Figure 11F p-c-Jun (S63)

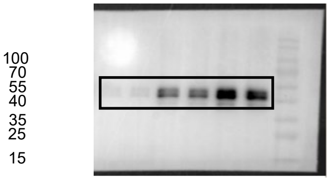

Supplemental Figure 11F p-c-Jun (S73)

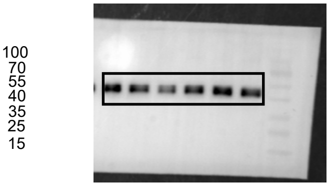

Supplemental Figure 11F c-Jun

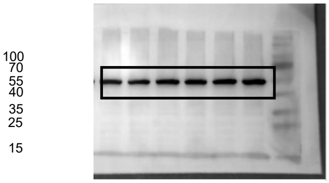

Supplemental Figure 11F Tubulin

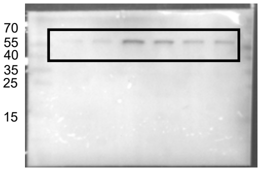

Supplemental Figure 11I ZBP1

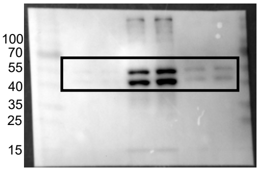

Supplemental Figure 11I p-JNK

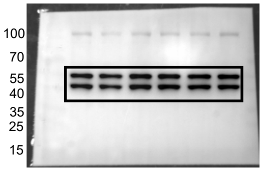

Supplemental Figure 11I JNK

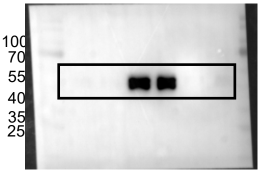

Supplemental Figure 11I p-c-Jun (S63)

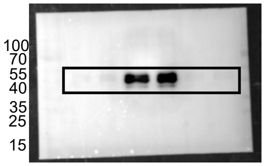

Supplemental Figure 11I p-c-Jun (S73)

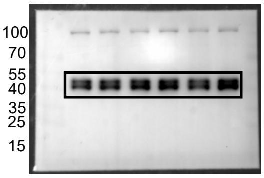

Supplemental Figure 11I c-Jun

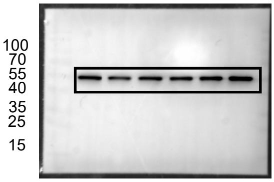

Supplemental Figure 11I Tubulin

Supplemental Figure 12

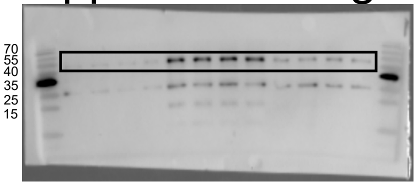

Supplemental Figure 12B ZBP1

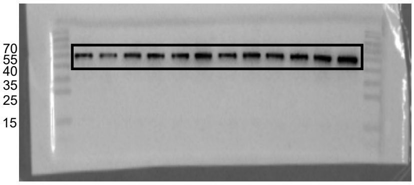

Supplemental Figure 12B Tubulin

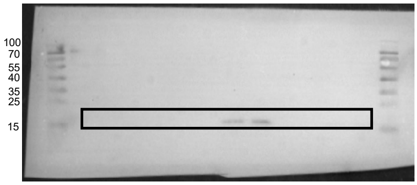

Supplemental Figure 12E CC3

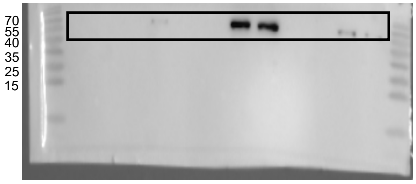

Supplemental Figure 12B p-RIPK1

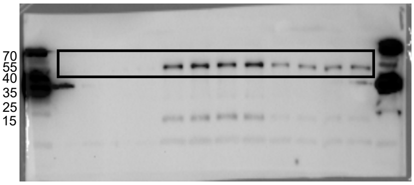

Supplemental Figure 12E ZBP1

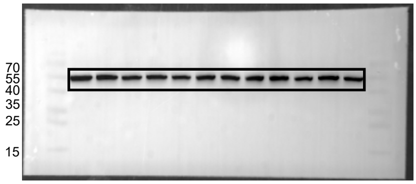

Supplemental Figure 12E Tubulin

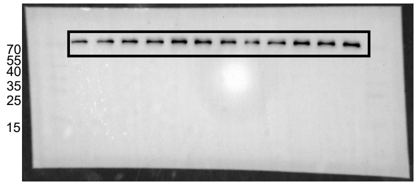

Supplemental Figure 12B RIPK1

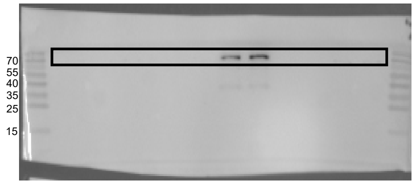

Supplemental Figure 12E p-RIPK1

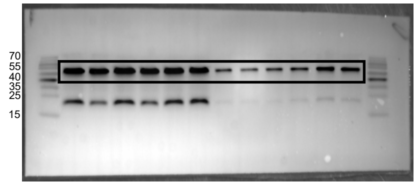

Supplemental Figure 12H ZBP1

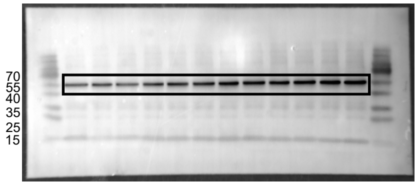

Supplemental Figure 12B Caspase 8

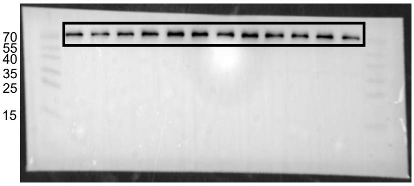

Supplemental Figure 12E RIPK1

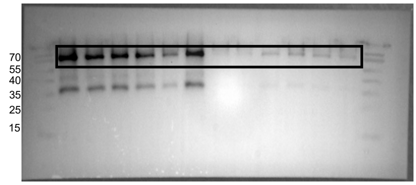

Supplemental Figure 12H p-RIPK1

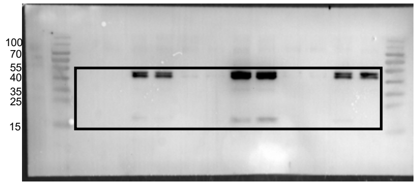

Supplemental Figure 12B CC8

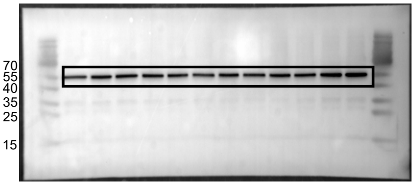

Supplemental Figure 12E Caspase 8

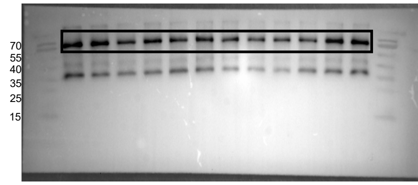

Supplemental Figure 12H RIPK1

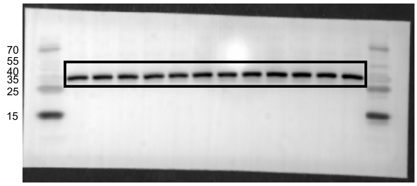

Supplemental Figure 12B Caspase 3

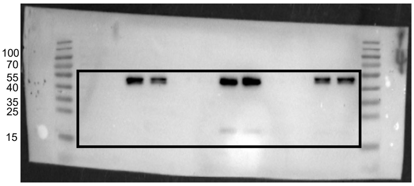

Supplemental Figure 12E CC8

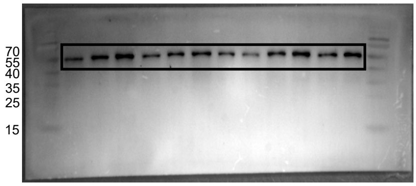

Supplemental Figure 12H Caspase 8

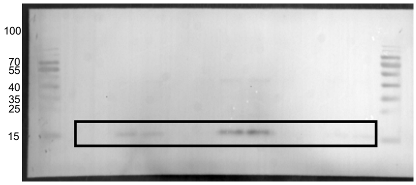

Supplemental Figure 12B CC3

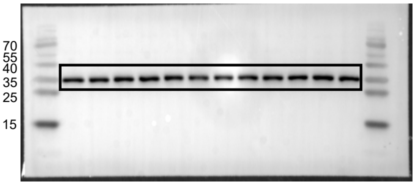

Supplemental Figure 12E Caspase 3

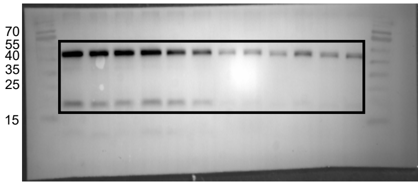

Supplemental Figure 12H CC8

Supplemental Figure 12

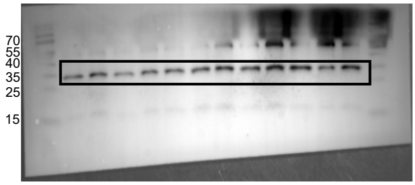

Supplemental Figure 12H Caspase 3

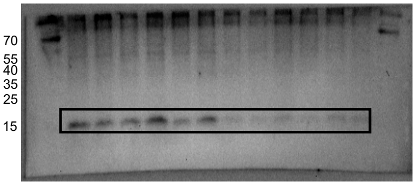

Supplemental Figure 12H CC3

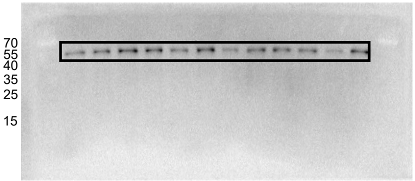

Supplemental Figure 12H Tubulin

Supplemental Figure 13

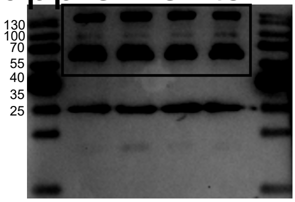

Supplemental Figure 13G ZBP1 (Non reducing)

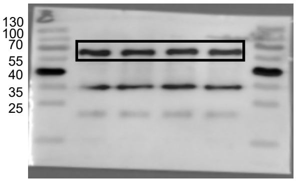

Supplemental Figure 13G ZBP1 (Reducing)

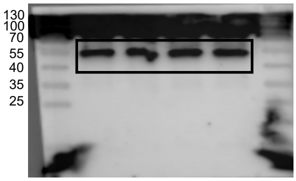

Supplemental Figure 13G Tubulin
